# Ti-superoxide catalyzed oxidative amidation of aldehydes with saccharin as nitrogen source: synthesis of primary amides†

**DOI:** 10.1039/c9ra10413e

**Published:** 2020-01-02

**Authors:** Rohit B. Kamble, Kishor D. Mane, Bapurao D. Rupanawar, Pranjal Korekar, A. Sudalai, Gurunath Suryavanshi

**Affiliations:** Chemical Engineering and Process Development Division, CSIR-National Chemical Laboratory Pune Maharashtra India-411 008 gm.suryavanshi@ncl.res.in; Academy of Scientific and Innovative Research Ghaziabad UP India-201002; Department of Chemistry, MES Abasaheb Garware College Pune India-411004

## Abstract

A new heterogeneous catalytic system (Ti-superoxide/saccharin/TBHP) has been developed that efficiently catalyzes oxidative amidation of aldehydes to produce various primary amides. The protocol employs saccharin as amine source and was found to tolerate a wide range of substrates with different functional groups. Moderate to excellent yields, catalyst reusability and operational simplicity are the main highlights. A possible mechanism and the role of the catalyst in oxidative amidation have also been discussed.

## Abstract 2

Heterogeneous catalytic system using Ti-superoxide and saccharin with TBHP has been developed which catalyzes oxidative amidation of aldehyde to produce primary amides. It tolerates a wide range of substrates with different functional groups.

## Graphical abstract

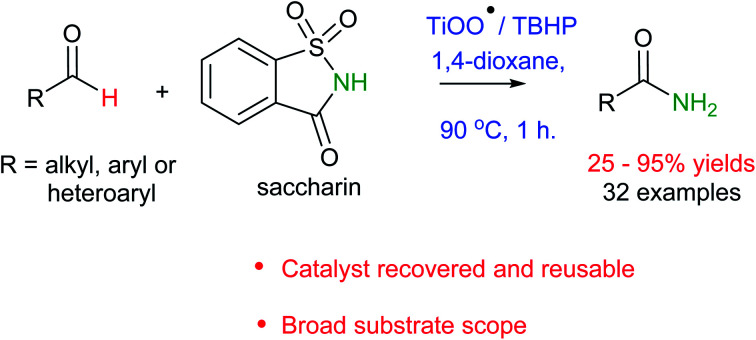

## Introduction

**Fig. 1:**
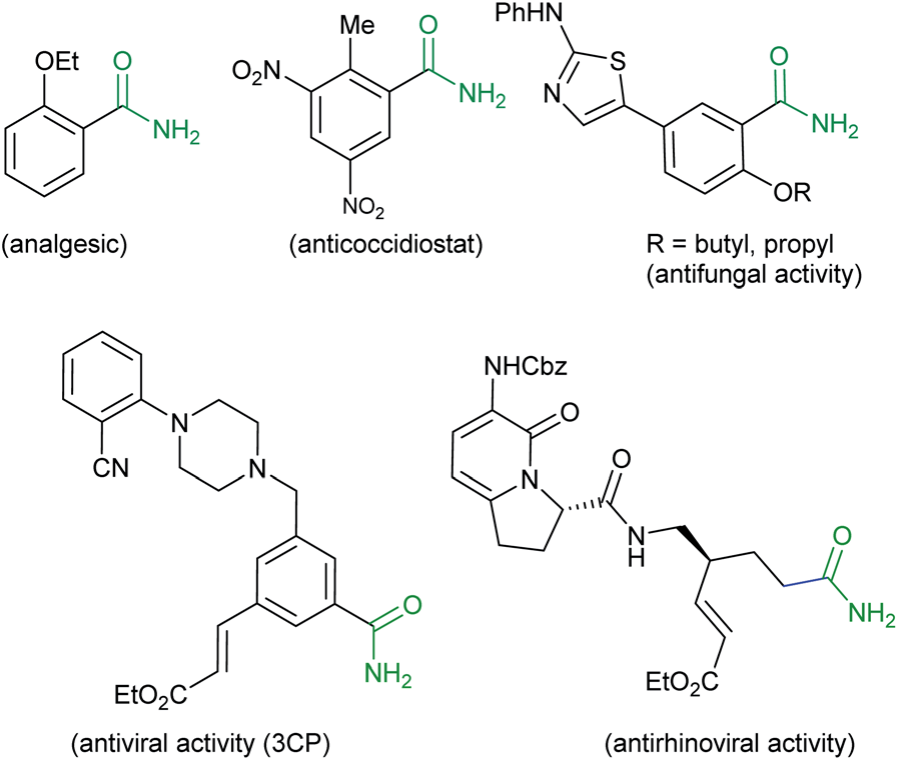
Some biologically important primary amides.

The amide bond constituting the structural backbone of proteins and peptides is abundantly found in natural products, pharmaceuticals, polymers and agrochemicals.^1^ In particular, primary amides (RCONH_2_) play an important role in organic synthesis as building blocks exhibiting a wide range of industrial applications and pharmacological interests^2^ (Fig. 1).

**Scheme 1:**
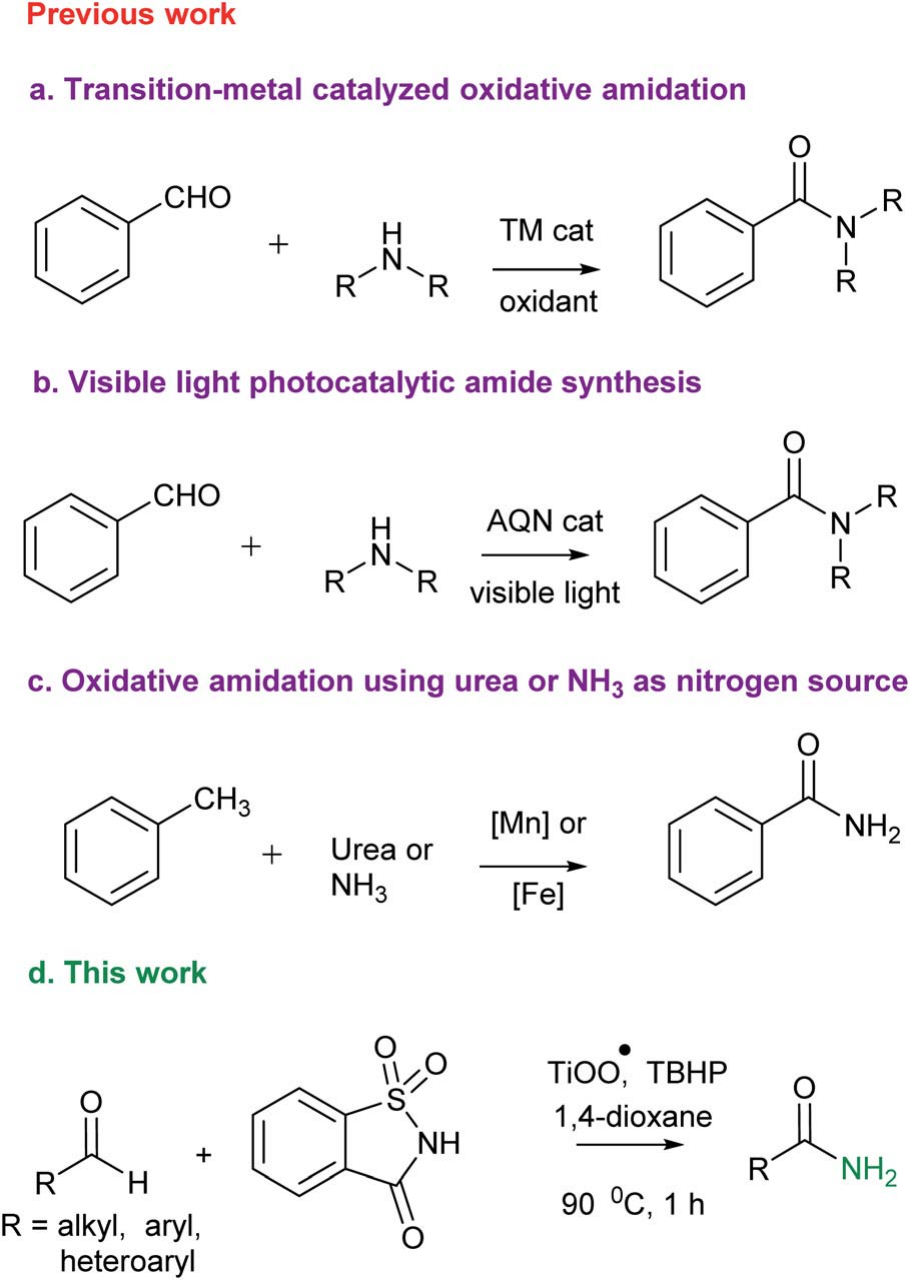
Primary amide synthesis *via* direct oxidative amidation of aldehydes or methyl arenes.

Traditionally, amide synthesis has been achieved by the reaction of an amine with an activated carboxylic acid derivative, that often employs coupling reagents.^3^ Subsequently, several alternate strategies^4^ emerged for amide formation that include: (i) the Staudinger reaction; (ii) the Schmidt reaction; (iii) the Beckmann rearrangement; (iv) hydroamination of alkynes; (v) dehydrogenative amidation of alcohols; (vi) hydroamino carbonylation of alkenes; (vii) iodonium promoted nitroalkene amine coupling reaction; (viii) transamidation of primary amides; *etc.* In this context, oxidative amidation of aldehydes with amine salts is synthetically preferred and has been achieved with a variety of reagent systems^5^ (*e.g.* I_2_, NBS, MnO_2_, 3,3′,5,5′-tetra-*tert*-butyldiphenoquinone and TBHP as oxidant, *N*-heterocyclic carbene, transition metals such as Pd, Rh, Ru, Ni, Cu/Ag, Fe. Au, Pt and lanthanides). It may also be noted that several researchers have developed catalyst-free methods using TBHP and H_2_O_2_ as oxidants.^6^ Quite recently, visible light was utilized to trigger a photoredox catalytic oxidative amidation of aldehydes.^7^ This reaction, however, relied on phenazinium salt, rose bengal or anthraquinone-based organophotocatalyst and air as the oxidant. Also, oxidative amidation of methylarenes catalyzed by Mn or Fe in combination with NH_3_ or urea as amine source and oxidants has been reported^8^ for amide synthesis (Scheme 1). The existing methods utilize homogeneous, rare and costly transition metals as catalyst. Also, these homogeneous reaction mixtures did not allow recyclability of used metals. To the best of our knowledge, metal catalyzed direct oxidative amidation of aldehydes under heterogeneous conditions has not explored. In this strategy, we wish to report Ti-superoxide catalyzed oxidative amidation of aldehydes and catalyst reused for more than three catalytic cycles (Scheme 1).

Saccharin (2) is an artificial sweetener used in the production of various foods and pharmaceutical products. It is also used in the preparation of disubstituted amines from halides *via* nucleophilic substitution followed by Gabriel synthesis.^9^

Sometime ago, we have reported an elegant synthesis and catalytic applications of exceptionally stable titanium superoxide for C–O, C–N and C–Br bond forming reactions.^10^ Keeping this in mind, it was of interest to explore the cross dehydrogenative coupling between benzaldehyde and saccharin under Ti-superoxide catalysis in the presence of TBHP as oxidant to produce *N*-benzoylsaccharin (8). Surprisingly, the reaction underwent oxidative amidation to produce benzamide (56%). Thus, in seeking to develop a general condition for amide synthesis, we proposed that saccharin (2) could serve as nitrogen source. In this paper, we wish to report, for the first time, that titanium superoxide efficiently catalyzes oxidative amidation of aldehydes, under truly heterogeneous conditions, to produce primary amides (3) in excellent yields employing saccharin (2) as amine source and TBHP as oxidant (Scheme 1).

Table 1 shows the results of optimization studies of oxidative amidation of anisaldehyde with saccharin as amine source over Ti-superoxide using TBHP as oxidant.

**Table 1:** Optimization of oxidative amidation of anisaldehyde with saccharin as amine source over Ti-superoxide^a^

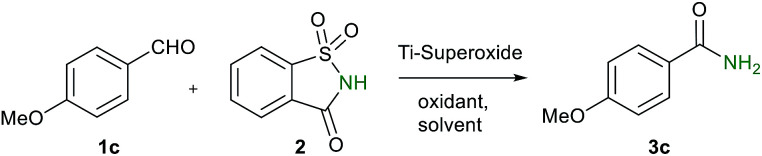						
No.	Cat^b^ (wt%)	Oxidant (equiv.)	Solvent	*T* (°C)	*T* (h)	Yield^c^ (%)
1	10	TBHP (1)^d^	DCE^e^	25	12	N R
2	10	TBHP (2)	DCE	90	12	19 (22)^f^
3	10	TBHP (3)	1,4-Dioxane	90	12	41 (22)^g^
4	20	TBHP (3)	1,4-Dioxane	90	12	65 (71)^h^, (83)^i^
5	20	TBHP (3)	1,4-Dioxane	90	1	95 (51)^j^
6	20	DTBP (3)^k^ or K_2_S_2_O_8_ (3)	1,4-Dioxane	90	1	N R
7	20	30% H_2_O_2_ (3)	1,4-Dioxane	90	1	11

a Reaction conditions: anisaldehyde (1 mmol), saccharin (1.2 mmol), solvent (4 mL).

b Titanium superoxide.

c Isolated yield.

d 5−6 M TBHP in hexane was used.

e 1,2-Dichloroethane.

f 3 equiv. of TBHP was used.

g Temperature was 110 °C.

h Time was 8 h.

i Time was 4 h.

j 2 equiv. of TBHP was used.

k Di-*tert*-butylperoxide.

When they were combined in equimolar amounts in 1,2-dichloroethane with Ti-superoxide (10 wt%) as catalyst at RT, no reaction took place. However, with increase in TBHP concentration (2–3 equiv.) and temperature at 90 °C, amide 3c was indeed obtained in low yields (19–22%). With change of solvent to 1,4-dioxane, considerable improvement in yield of 3c was achieved (41%). Further, increase of temperature to 110 °C, however, had a deleterious effect on yield (22%) (entry 3). When the catalyst concentration was increased to 20 wt%, yield of 3c was increased to 65%. Interestingly, a substantial improvement in yield was observed from 65 to 83% as the reaction time was decreased from 12 h to 4 h. Finally, a dramatic improvement in yield (95%) was realized with the reduction in time to 1 h. Further, reduction in TBHP concentration to 2 equiv. resulted in lowered yield of amide 3c (51%) (entry 5). Unfortunately, other solvents such as CH_3_CN, DMSO, DMF and THF were found to be unsuitable for the reaction. Also, several oxidants such as DTBP, K_2_S_2_O_8_ and H_2_O_2_ and other Ti catalysts (titanium silicalite-1 and TiO_2_) were found to be less favoured for oxidative amidation. It may be noted that the reaction failed to proceed with other amine sources such as ammonia or its salts (Cl^−^, OAc^−^ or NO_3_^−^) as well.

To determine the scope and limitations of this reaction, a wide range of aldehydes were reacted under the optimized reaction conditions (Table 2). In general, good to excellent yields of primary amides were obtained in most cases. For instance, aromatic aldehydes, bearing electron–donating and electron withdrawing groups in different ring positions, gave the desired products (3a–w) in good to excellent yields (27–95%), indicating that the reaction is not sensitive to electronic effects. Thus, various functional groups with potential synthetic applications are well-suited for this reaction, although substrates having sensitive NH_2_ and OH groups gave a diminished yield (27–36%). Interestingly, phenyl acetaldehydes possessing a variety of substituents with different electronic effect (Br, OH, OMe and Cl) gave the desired primary amides (3x–z & 3aa-3ab) in high yields (36–86%). Aliphatic (C_8_- and C_10_-), heteroaryl (2-thienyl and 2-pyridyl), and naphthyl aldehydes were tolerated as well, thus providing the desired amides (3ae, 3af & 3i) in good yields (25–78%). Nevertheless, it should be noted that unsaturated aldehydes such as cinnamaldehyde and acrolein are less favoured substrates under the oxidative amidation condition.

**Table 2:** Substrate scope for the oxidative amidation of aldehydes^a^

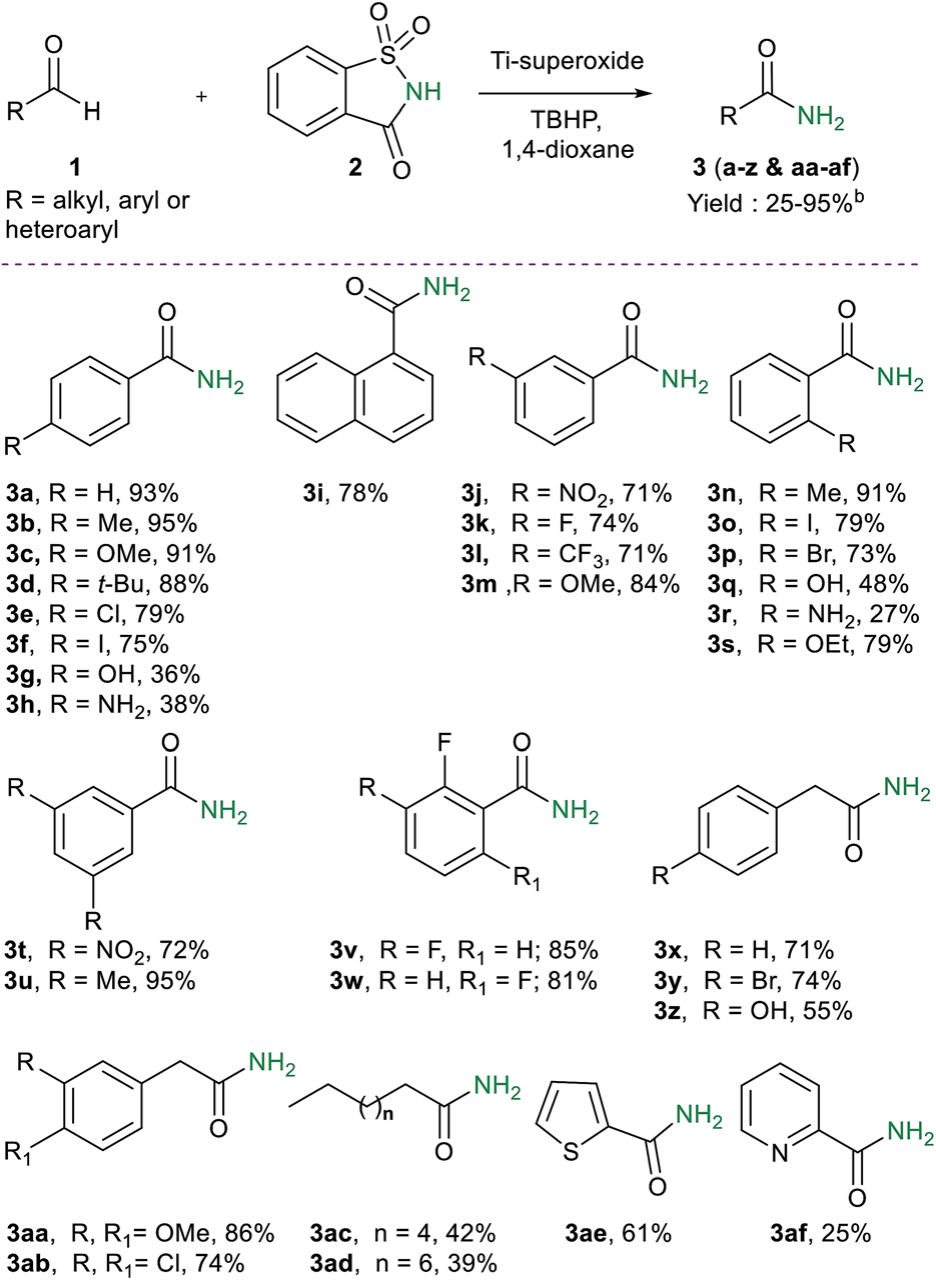

a Reaction conditions: aldehyde (1 mmol), saccharin (1.2 mmol), 5–6 M TBHP in hexane (3 mmol), Ti-superoxide (20 wt%), 1,4-dioxane (4 mL), 90 °C, 1 h.

b Isolated yield.

**Scheme 2:**
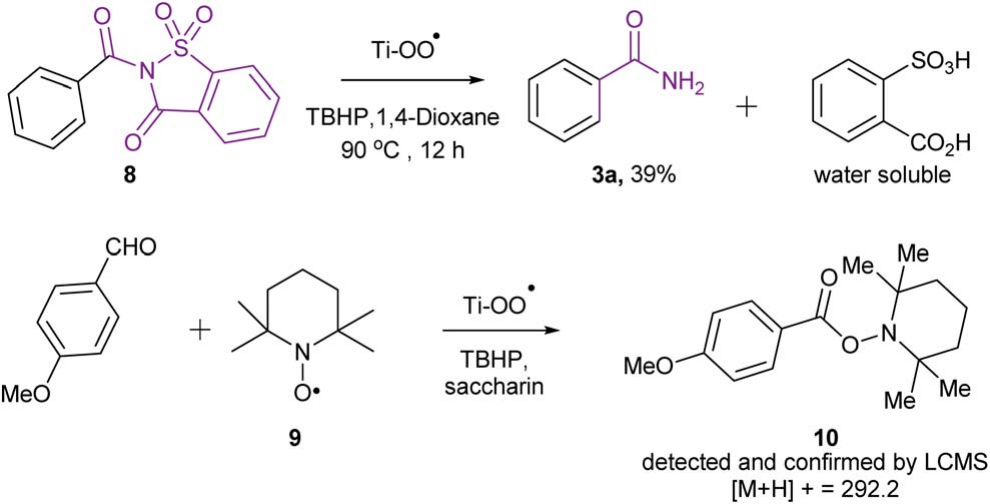
Mechanistic studies to establish the involvement of radical pathway.

**Scheme 3:**
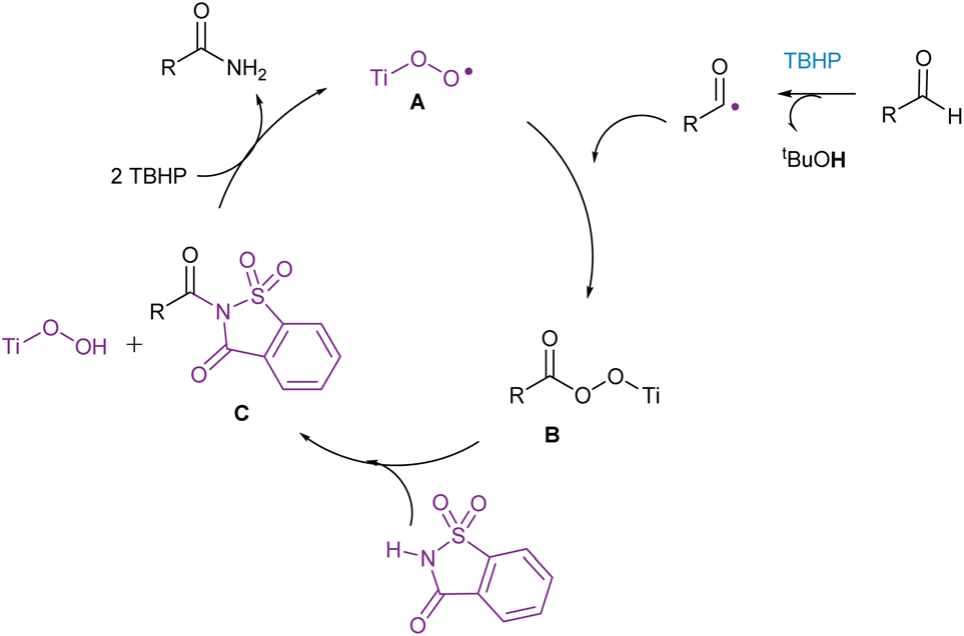
Catalytic cycle for the oxidative amination of aldehydes.

In order to get an insight into the mechanism of this reaction, we have conducted the following two experiments (Scheme 2). When *N*-benzoyl saccharin 8 was subjected to the optimized reaction conditions, benzamide (3a) was indeed isolated in 39% yield, confirming the involvement of 8 as the key intermediate. Also, when oxidative amidation of anisaldehyde was carried out in the presence of radical scavenger TEMPO (1.1 equiv.), the corresponding TEMPO adduct 10 was detected and confirmed by LCMS, thus establishing the formation of benzoyl radical that underwent radical coupling in the reaction. On the basis of the above experiments and literature precedence,^11^ a plausible catalytic cycle is proposed in Scheme 3. Initially, combination of acyl radical, generated from aldehyde on oxidation with TBHP, in the presence of Ti catalyst A produces Ti peroxo species B. Subsequently, B undergoes displacement with saccharin to produce *N*-acylsaccharin C along with TiOOH. Finally, 2 equiv. of TBHP are utilized: (i) to regenerate Ti catalyst A; (ii) to form amides from intermediate C*via* oxidative hydrolysis.

**Scheme 4:**
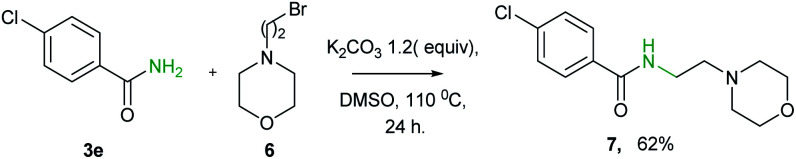
Synthesis of moclobemide on 5 g scale.

This methodology is amply demonstrated in the synthesis of drugs namely ethenzamide 3s and moclobemide 7. Scheme 4 shows the single step synthesis of moclobemide, a reversible inhibitor of monoamine oxidase A*via N*-alkylation of 3e with 6.

**Fig. 2:**
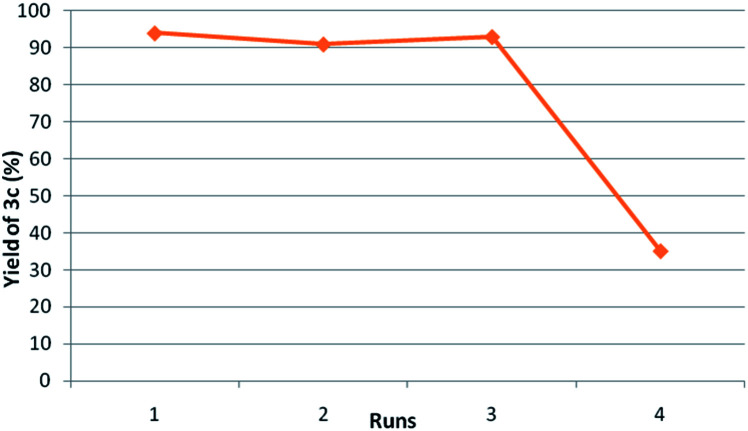
Reusability studies of Ti catalyst. ^*a*^Reaction conditions: anisaldehyde (2 mmol), saccharin (2.4 mmol), TBHP (6 mmol), 1,4-dioxane, 90 °C, 1 h; ^*b*^isolated yiled.

Fig. 2 shows the results on reusability studies. Ti-superoxide catalyst was readily recovered quantitatively by simple filtration and reused again at least for 3 cycles without the loss of catalytic activity (runs 1–3). The catalyst performed under truly heterogeneous manner as no leaching of Ti was observed in the aqueous part.

## Conclusions

In conclusion, we have described here a simple, convenient and environment-friendly protocol for primary amide synthesis directly from aldehydes using Ti-superoxide as a mild and cheap catalyst and saccharin as amine source using TBHP as oxidant. The presented strategy has several advantages that include: (i) Ti catalyst is recyclable; (ii) good functional group compatibility; (iii) wide range of substrate scope; (iv) mild reaction conditions; (v) no additives and can be easily scaled up; (vi) saccharin as cheaply available amine source. We envisage that this new catalytic method would be used as an alternative to other existing methods for the primary amide synthesis.

## Conflicts of interest

There are no conflicts to declare.

## Supplementary Material

RA-010-C9RA10413E-s001
