# Supplementary material for: Ti-superoxide catalyzed oxidative amidation of aldehydes with saccharin as nitrogen source: synthesis of primary amides
Source: RSC Adv. 2020 Jan 2;10(2):724–8. doi: 10.1039/c9ra10413e (PMC9047451; doi:10.1039/c9ra10413e)
Supplement: RA-010-C9RA10413E-s001 [file RA-010-C9RA10413E-s001.pdf]

Electronic Supplementary Material (ESI) for RSC Advances.
This journal is © The Royal Society of Chemistry 2019

Electronic Supplementary Information (ESI)

Ti-superoxide catalysed oxidative amidation of aldehydes with saccharin as nitrogen source: synthesis of primary amides

Rohit B. Kamble [a,b,ł] Kishor D. Mane,[a,b,ł] Bapurao D. Rupanawar,[a,b] Pranjal Korekar,[a,c] A. sudalai [a,b] and Gurunath Suryavanshi*[a,b]

[a]*Chemical Engineering and Process Development Division, CSIR-National Chemical Laboratory, Dr. Homi Bhabha Road, Pune-411 008.*

[b]*Academy of Scientific and Innovative Research, Ghaziabad 201 002, India.*

[c]*Department Of Chemistry, MES Abasaheb Garware College, Pune, India-411 004.*

†*These authors contribute equally*

*Corresponding author: Tel.: +91 20 25902547; Fax: +91 20 25902676;

E-mail:gm.suryavanshi@ncl.res.in

Table of Contents

Sr.No.	Description	Page No.
1	General information	S3
2	General procedure for the Preparation of the titanium superoxide catalyst	S2
3	General procedure for the synthesis of compound 3a-az &3aa-3af	S2
4	General Procedure for the Gram Scale Synthesis of Moclobemide (7)	S2-S3
5	NMR spectral data	S4-S15
6	^{1}H and ^{13}C NMR spectrums	S16-S50
7	LC-MS spectrums of compound (7)& (10)	S51- S52
8	^{19}F NMR spectrums of 3k,3l,3v,3w.	S52- S54

General information:

Solvents were purified and dried using standard procedures before use. All air- and moisture-sensitive reactions were carried out in flame-dried glassware under a positive pressure of dry argon using standard techniques. All reagents and solvents were obtained from commercial suppliers and Aldrich, Merck Millipore, Alfa Aesar and Avra Synthesis. Aldehydes were purified either by distillation or washing with $NaHCO_3$. For moisture-sensitive reactions, 1, 4-Dioxane were dried using a standard solvent purification system. Technical solvents for column chromatography were used after simple distillation. The reactions were monitored by TLC visualized by UV (254 nm). The purification was done using column chromatography on silica (Merck, 100−200 mesh) with the indicated eluent mixtures (v/v) Nuclear magnetic resonance spectra were recorded at room temperature on Bruker AV-200, AV-400, and AV-500 spectrometers in appropriate solvents using TMS as an internal standard or the solvent signals as secondary standards, and the chemical shifts are shown in δ scales. Coupling constants (J) are given in hertz (Hz), and the classical abbreviations are used to describe the signal multiplicities. Deuterated solvent such as $CDCl_3$, DMSO were used as internal standard. The septet at 2.50ppm in ^{1}H NMR and 39.51 ppm in ^{13}C NMR corresponds to Hydrogen and carbon of DMSO-D_6. The peaks at 7.27 ppm in ^{1}H NMR and 77.00 ppm in ^{13}C NMR corresponds to proton and carbon of $CDCl_3$. Apparent multiplets, which occur as a result of coupling constant equality between magnetically non-equivalent protons, are marked as virtual (virt). The following abbreviations for single multiplicities were used: br = broad, s = singlet, d = doublet, t = triplet, q = quartet, m = multiplet. High-resolution mass spectra (HRMS) for all new compounds were recorded on an ESI^+ method and Orbitrap mass analyzer (Thermo Scientific Q-Exactive, Accela 1250 pump).

Preparation of the titanium superoxide catalyst:

To a stirred solution of 50% aq. H_2O_2 (3 g, 0.0875 mmol) was added titanium tetraisopropoxide ($Ti(OiPr)_4$ (2.51 g, 0.0875 mmol) in anhydrous methanol (30 mL) for 30 min under a nitrogen atmosphere with continuous stirring at room temp for 2 h. The yellow colour solid formed was filtered on a sintered funnel, washed thoroughly with anhydrous methanol, and dried under reduced pressure (3 mm Hg) at 25 ºC for 1 h to give titanium superoxide 96% yield.

General experimental procedure for the preparation of benzamides:

To a 25 mL oven dried round-bottom flask were added benzaldehydes (1 equiv.), Saccharin (1.2 equiv.) and titanium superoxide as catalyst (10 wt.%) in dry 1,4-Dioxane (4mL) was added TBHP in decane (3 equiv.) in a drop wise manner. The round-bottom flask was equipped with a condenser, and the resulting reaction mixture was refluxed to 90˚C for 1h. The progress of the reaction was monitored by TLC. Upon completion of the reaction, the solvent was evaporated under reduced pressure then reaction mixture was filtered through a sintered funnel using $NaHCO_3$ aqueous solution and EtOAc as an eluent. Then the organic layer was extracted with EtOAc, dried over anhydrous Na_2SO_4, and evaporated under reduced pressure. The crude product was purified by column chromatography over silica (100-200 mesh) using petroleum ether/ethyl acetate (70:30 v/v) as an eluent to give the corresponding amides.

General procedure for the gram scale synthesis of Moclobemide (7) :

4-chlorobenzamide (5 g, 1equiv.), K_2CO_3 (6.67 g 1.5 equiv.), 4-(2-bromoethyl) morpholine (7.41 g 1.2 equiv.), and dry DMSO (25 mL) were placed in a two-neck round bottom flask. the mixture was heated at 110 ˚C for 24 h under nitrogen atmosphere, then cooled, diluted with cold aq NH_4Cl solution and extracted with EtOAc (3*30 mL). The organic layers were combined, washed with brine, dried over anhyd. Na_2SO_4, and then concentrated. Further the product was purified by column chromatography.

NMR Spectral Data

Benzamide (3a)

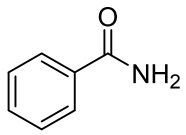

yield: 104 mg (93%); White solid; mp:130-132 °C; **^{1}H NMR** (400 MHz, DMSO-d_6): δ 7.38 - 7.55 (m, 4H), 7.88 - 7.98 (m, 2H), 8.07 (br.s.,1H); **^{13}C NMR** (101 MHz, *DMSO*-d_6): δ 128.0, 128.7, 131.7, 134.7, 168.6. **HRMS** (ESI) calculated $[M+H]^+$ for C_7H_8ON: 122.0527; found: 122.0528.

4-Methylbenzamide (3b)

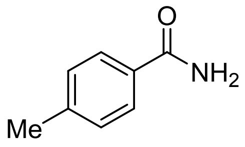

yield: 106 mg (95%); white solid; mp: 158-160°C; **^{1}H NMR** (400 MHz, *DMSO*-d_6) : δ 2.33 (s, 3H), 7.23 (d, J = 7.9 Hz, 2H), 7.31 (br. s., 1H), 7.79 (d, J = 7.9 Hz, 2H), 7.92 (br. s., 1H); **^{13}C NMR** (101 MHz, *DMSO*-d_6): δ 21.4 , 128.0, 129.2, 131.9, 141.5, 168.3; **HRMS** (ESI) calculated $[M+H]^+$ for $C_8H_{10}ON$: 136.0684; found: 136.0684.

4-Methoxybenzamide (3c)

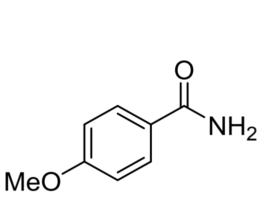

Yield: 101 mg (91%); White solid; mp: 166-170°C; **^{1}H NMR** (400 MHz, *DMSO*-d_6): δ 3.80 (br. s., 3H), 6.96 (br. s., 2H), 7.21 (br. s., 1H), 7.85 (br. s., 3H); **^{13}C NMR** (101 MHz, *DMSO*-d_6): δ 55.5, 113.6, 126.7, 129.6, 161.8, 167.7, and 167.7; **HRMS** (ESI) calculated $[M+H]^+$ for

$C_8H_{10}O_2N$: 152.0706; found: 152.0706.

4-(Tert-butyl)benzamide (3d)

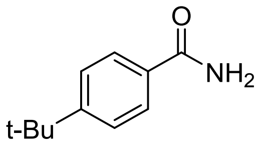

yield: 96 mg (88%); white solid; mp: 172-174°C; **^{1}H NMR** (400 MHz, *DMSO-d_6*): δ 1.26 (br. s., 9H), 7.32 (br. s., 1H), 7.44 (d, *J* = 8.2 Hz, 2H), 7.81 (t, *J* = 6.6 Hz, 2H), 7.95 (br. s., 1H); **^{13}C NMR** (101 MHz, *DMSO-d_6*): δ 31.0, 34.6, 125.0, 127.4, 131.6, 154.1, 168; **HRMS** (ESI) calculated $[M+H]^+$ for $C_1H_{16}ON$: 178.1226; found: 178.1227.

4-Chlorobenzamide(3e)

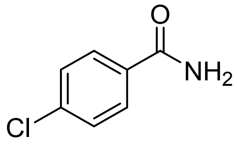

yield: 87 mg (79%); White solid; mp: 178-180°C; **^{1}H NMR** (400 MHz, *DMSO-d_6*): δ 7.38 - 7.64 (m, 3H), 7.89 (d, *J* = 7.9 Hz, 2H), 8.06 (br. s., 1H); **^{13}C NMR** (101 MHz, *DMSO-d_6*): δ 128.6, 129.7, 133.3, 136.4, 167.2; **HRMS** (ESI) calculated $[M+H]^+$ for C_7H_7ONCl: 156.0137; found: 156.0139.

4-Iodobenzamide(3f)

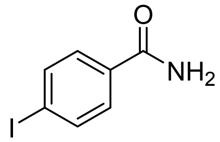

yield: 79 mg (75%); White solid; mp: 214-218°C; **^{1}H NMR** (400 MHz, *DMSO-d_6*): δ 7.48 (br. s., 1H), 7.66 (d, *J* = 6.9 Hz, 2H), 7.75 - 7.92 (m, 2H), 8.07 (br. s., 1H); **^{13}C NMR** (101 MHz, *DMSO-d_6*) : δ 99.5, 130.0, 134.2, 137.6, 167.9; **HRMS** (ESI) calculated $[M+H]^+$ for

C_7H_7ONI: 247.9567; found: 247.9569

4-Hydroxybenzamide(3g)

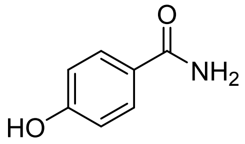

yield: 42 mg (36%); off white solid; mp: 158-160°C; **^{1}H NMR** (400 MHz, *DMSO-d_6*): δ 6.81 (d, *J* = 8.5 Hz, 2H), 7.17 (br. s., 1H), 7.77 (d, *J* = 8.5 Hz, 3H), 10.00 (s, 1H); **^{13}C NMR** (101 MHz, *DMSO-d_6*) : δ 115.6, 125.7, 130.3, 161.0, 168.8; **HRMS** (ESI) calculated $[M+H]^+$ for $C_7H_8O_2N$: 138.0550; found: 138.0550.

4-Aminobenzamide(3h)

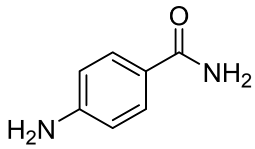

yield: 41 mg (38%); white solid; mp: 182-184°C; **^{1}H NMR** (400 MHz, *DMSO-d_6*) : δ 5.66 (br. s., 2H), 6.63 (d, *J* = 7.9 Hz, 2H), 7.15 (br. s., 1H), 7.73 (d, *J* = 7.3 Hz, 3H); **^{13}C NMR** (101 MHz, *DMSO-d_6*) : δ 113.0, 121.1, 129.6, 152.0, 169.1; **HRMS** (ESI) calculated $[M+H]^+$ for $C_7H_9ON_2$: 137.0709; found: 137.0711.

1-Naphthamide(3i)

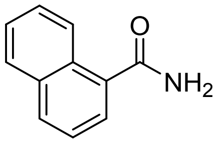

yield: 91 mg (78%); brown solid; mp: 208−210 °C; **^{1}H NMR** (400 MHz, *DMSO-d_6*): δ 7.47 - 7.75 (m, 5H), 7.90 - 8.13 (m, 3H), 8.27 - 8.43 (m, 1H); **^{13}C NMR** (101 MHz, *DMSO-d_6*) : δ 125.3, 125.5, 126.0, 126.5, 127.0, 128.6, 130.1, 130.2, 133.6, 135.1, 171.0; **HRMS** (ESI) calculated $[M+H]^+$ for $C_{11}H_{10}ON$: 172.0757; found:

172.0757.

3-Nitrobenzamide(3j)

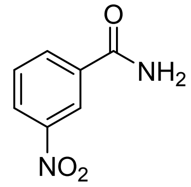

yield: 78 mg (71%); white solid; mp: 140-142°C; **^{1}H NMR** (400 MHz, *DMSO-d$_6$*): δ 7.68 - 7.83 (m, 2H), 8.35 (d, *J* = 7.3 Hz, 2H), 8.30 (d, *J* = 7.9 Hz, 2H), 8.68 (s, 1H). **^{13}C NMR** (101 MHz, *DMSO-d$_6$*): δ 122.3, 125.9, 130.1, 133.9, 135.8, 147.8, 165.9; **HRMS** (ESI) calculated $[M+H]^+$ for $C_7H_7O_3N_2$: 167.0451; found: 167.0452.

3-Fluorobenzamide(3k)

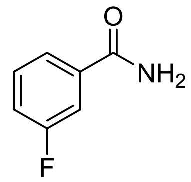

yield: 83 mg (74%); white solid; mp: 128-130°C; **^{1}H NMR** (400 MHz, *DMSO-d$_6$*): δ 7.31 (br. s., 1H), 7.45 (br. s., 1H), 7.52 (br. s., 1H), 7.60 - 7.74 (m, 2H), 8.06 (br. s., 1H); **^{13}C NMR** (101 MHz, *DMSO-d$_6$*): δ 114.6, 118.4, 124.0, 130.8, 137.1, 163.7, 167.1.

3-(Trifluoromethyl)benzamide(3l)

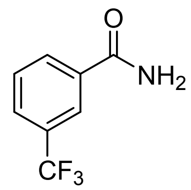

yield: 77 mg (71%); white solid; mp: 182-186°C; **^{1}H NMR** (400 MHz, *DMSO-d$_6$*): δ 7.55 - 7.79 (m, 2H), 7.87 (d, *J* = 7.3 Hz, 1H), 8.14 - 8.44 (m, 3H); **^{13}C NMR** (101 MHz, *DMSO-d$_6$*) : δ 123.1, 124.6, 125.8, 128.3, 129, 130.0, 132.0, 135.6, 166.9; **HRMS** (ESI) calculated $[M+H]^+$ for $C_8H_7ONF_3$: 190.0447; found: 190.0447.

3-Methoxybenzamide(3m)

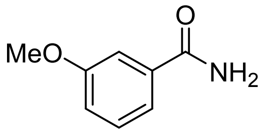

yield: 94 mg (84%); white solid; mp: 182-186°C; **^{1}H NMR** (400 MHz, *DMSO-d_6*): δ 3.79 (s, 3H), 6.92 - 7.20 (m, 1H), 7.29 - 7.58 (m, 4H), 7.99 (br. s., 1H); **^{13}C NMR** (101 MHz, *DMSO-d_6*): δ 55.5, 112.9, 117.3, 120.0, 129.6, 136.0, 159.4, 168.0; **HRMS** (ESI) calculated $[M+H]^+$ for $C_8H_{10}O_2N$: 152.0706; found: 152.0706.

2-Methylbenzamide(3n)

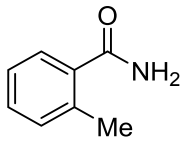

yield: 102 mg (91%); white solid; mp:142-146°C; **^{1}H NMR** (400 MHz, *DMSO-d_6*): δ 2.36 (s, 3H), 7.16 - 7.25 (m, 2H), 7.28 - 7.40 (m, 3H), 7.70 (br. s., 1H); **^{13}C NMR** (101 MHz, *DMSO-d_6*): δ 20.0, 125.9, 127.4, 129.6, 130.9, 135.6, 137.5, 171.5; **HRMS** (ESI) calculated $[M+H]^+$ for $C_8H_{10}ON$: 136.0684; found: 136.0684

2-Iodobenzamide(3o)

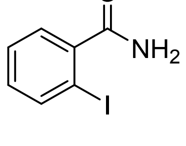

yield: 84 mg (79%); white solid; mp: 180-182°C; **^{1}H NMR** (400 MHz, *DMSO-d_6*): δ 7.15 (td, *J* = 7.6, 1.6 Hz, 1H), 7.35 (dd, *J* = 7.6, 1.6 Hz, 1H), 7.43 (t, *J* = 7.4 Hz, 1H), 7.52 (br. s., 1H), 7.77 - 7.94 (m, 2H); **^{13}C NMR** (101 MHz, *DMSO-d_6*): δ 93.6, 128.3, 128.4, 131.1, 139.6, 143.6, 171.2; **HRMS** (ESI) calculated $[M+H]^+$ for

C_7H_7ONI: 247.9567; found: 247.9566

2-Bromobenzamide(3p)

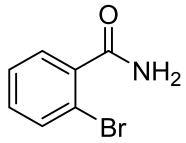

yield: 79 mg (73%); white solid; mp:156-158°C; **^{1}H NMR** (400 MHz, *DMSO-d_6*): δ 7.34 (br. s., 1 H), 7.41 (br. s., 2 H), 7.53 - 7.72 (m, 2H), 7.88 (br. s., 1H); **^{13}C NMR** (101 MHz, *DMSO-d_6*): δ 119.1, 128, 129, 131.1, 133.2, 139.8, 169.6

2-Hydroxybenzamide(3q)

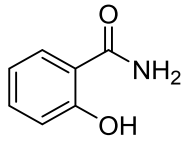

yield: 53 mg (48%); off white solid; mp:137-139°C; **^{1}H NMR** (400 MHz, *DMSO-d_6*): δ 6.76 - 6.96 (m, 2H), 7.29 - 7.44 (m, 1H), 7.78 - 8.04 (m, 2H), 8.44 (br. s., 1H); **^{13}C NMR** (101 MHz, *DMSO-d_6*): δ 115.1, 118.1, 119.0, 128.8, 134.7, 161.8, 172.9; **HRMS** (ESI) calculated $[M+H]^+$ for $C_7H_8O_2N$: 138.0550; found: 138.0550

2-Aminobenzamide(3r)

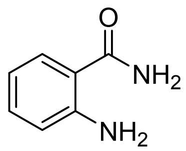

yield: 30 mg (27%); white solid; mp:111-113°C; **^{1}H NMR** (400 MHz, *DMSO-d_6*): δ 6.46 - 6.61 (m, 3H), 6.76 (d, J = 7.9 Hz, 1H), 7.09 - 7.33 (m, 2H), 7.62 (d, J = 7.9 Hz, 1H), 7.84 (br. s., 1H); **^{13}C NMR** (101 MHz, *DMSO-d_6*): δ 114.4, 115.4, 117.2, 129.5, 132.8 , 150.8 , 172.3; **HRMS** (ESI) calculated $[M+H]^+$ for $C_7H_9ON_2$:

137.0709; found: 137.0709

2-Ethoxybenzamide(3s)

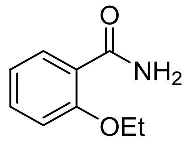

yield: 91 mg (79%); white solid; mp: 130-132 °C; **^{1}H NMR** (400 MHz, *DMSO-d_6*): δ 1.4 (t, *J* = 7.10 Hz, 3H), 4.1 (q, *J* = 6.87 Hz, 2H), 6.9 - 7.2 (m, 2H), 7.4 (td, *J* = 7.78, 1.83 Hz, 1H), 7.6 (br. s., 2H), 7.9 (dd, *J* = 7.79, 1.83 Hz, 1H); **^{13}C NMR** (101 MHz, *DMSO-d_6*): δ 14.5 ,64.3, 112.9, 120.5, 122.6, 131.0, 132.6, 156.6, 166.5; **HRMS** (ESI) calculated $[M+H]^+$ for $C_9H_{12}O_2N$: 166.0863; found: 167.0862.

3,5-Dinitrobenzamide(3t)

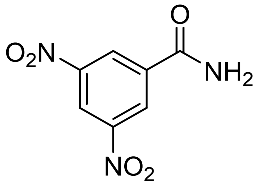

yield: 77 mg (72%); yellow solid; mp:180-182°C; **^{1}H NMR** (400 MHz, *DMSO-d_6*):7.99 (br. s., 1H), 8.67 (br. s., 1H), 8.85 - 8.99 (m, 2H), 9.04 (d, *J* = 1.8 Hz, 2H); **^{13}C NMR** (101 MHz, *DMSO-d_6*): δ 121.5, 128.4, 129.2, 137.7, 148.5, 148.8, 164.3; **HRMS** (ESI) calculated $[M+H]^+$ for $C_7H_6O_5N_3$: 212.0302; found: 212.1181.

3,5-Dimethylbenzamide(3u)

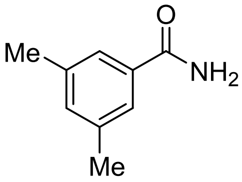

yield: 105 mg (95%); white solid; mp:134-136°C; **^{1}H NMR** (400 MHz, *DMSO-d_6*): δ 2.29 (s, 7H), 7.12 (s, 1H), 7.26 (br. s., 1H), 7.49 (s, 2H), 7.88 (br. s., 1H); **^{13}C NMR** (101 MHz, *DMSO-d_6*): δ 21.3, 125.7, 132.9, 134.7, 137.7,

168.7; **HRMS** (ESI) calculated $[M+H]^+$ for $C_9H_{12}ON$: 150.0913; found: 150.0913

2,3-Difluorobenzamide(3v)

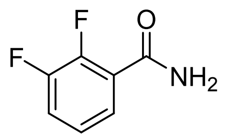

yield: 96 mg (85%); white solid; mp:118-120°C; **^{1}H NMR** (400 MHz, *DMSO-d_6*): δ 7.25 (br. s., 1H), 7.38 - 7.57 (m, 2H), 7.81 (br. s., 1H), 7.90 (br. s., 1H); **^{13}C NMR** (101 MHz, *DMSO-d_6*) : δ 119.4- 119.5 (1C *J* = 17 Hz), 125.0- 125.3 (1C *J* = 30 Hz), 126.6, 146.3 -146.4 (1C *J* = 12.5 Hz), 148.8 -148.9 (1C *J* = 13.5 Hz), 151.2- 151.4 (1C *J* = 13.5 Hz), 164.7; **HRMS** (ESI) calculated $[M+H]^+$ for $C_7H_6ONF_2$: 158.0412; found: 158.0413

2,6-Difluorobenzamide(3w)

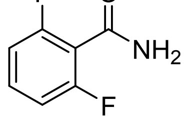

yield: 89 mg (81%); white solid; mp: 150-154°C; **^{1}H NMR** (400 MHz, *DMSO-d_6*): δ 7.14 (t, *J* = 8.0 Hz, 2H), 7.48 (quin, *J* = 7.6 Hz, 1H), 7.88 (br. s., 1H), 8.18 (br. s., 1H); **^{13}C NMR** (101 MHz, *DMSO-d_6*): δ 112.2-112.3 (1C *J* = 5 Hz), 112.4-112.4 (1C *J* = 5 Hz), 116.1, 116.3-116.5 (1C *J* = 23 Hz), 131.6, 131.7- 131.8 (1C *J* = 9.5 Hz), 157.9, 158.0 (1C *J* = 8.6 Hz), 160.4-160.5 (1C *J* = 8.6 Hz), 162.2; **HRMS** (ESI) calculated $[M+H]^+$ for $C_7H_6ONF_2$: 158.0412; found: 158.0413.

2-Phenylacetamide(3x)

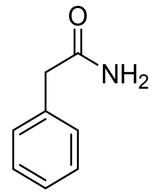

yield: 80 mg (71%); white solid; mp:154- 156°C; **^{1}H NMR** (400 MHz, *DMSO-d_6*): δ 3.48 (br. s., 2H), 6.94 (br. s., 1H), 7.21 - 7.26 (m, 1H), 7.28 (br. s., 5H), 7.52 (br. s., 1H); **^{13}C NMR** (101 MHz, *DMSO-d_6*) δ ppm 42.8, 126.8, 128.7, 129.6, 137.0, 172.9.

2-(4-Bromophenyl)acetamide(3y)

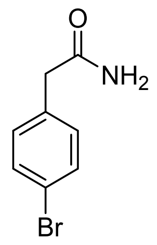

yield: 79 mg; (74%); yellow solid; mp: 194 - 196°C; **^{1}H NMR** (400 MHz, *DMSO-d_6*): δ 3.40 (br. s., 3H), 6.96 (br. s., 1H), 7.25 (br. s., 3H), 7.41 (br. s., 1H), 7.48 (br. s., 1H), 7.55 (br. s., 1H); **^{13}C NMR** (101 MHz, *DMSO-d_6*): δ 42.1, 121.8, 128.7, 129.6, 130.7, 132.3, 139.6, 172.2; **HRMS** (ESI) calculated $[M+H]^+$ for C_8H_9ONBr: 213.9862; found: 213.9868.

2-(4-Hydroxyphenyl)acetamide(3z)

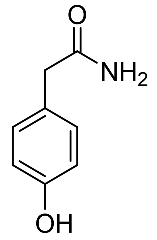

yield: 61 mg (55%); white solid; mp: 175- 177°C; **^{1}H NMR** (400 MHz, *DMSO-d_6*): δ 3.28 (br. s., 2H), 6.63 - 6.81 (m, 2H), 6.82 - 7.00 (m, 1H), 7.04 - 7.16 (m, 2H), 7.43 (br. s., 1H), 9.19 - 9.35 (m, 1H); **^{13}C NMR** (101 MHz, *DMSO-d_6*): δ 41.8, 115.4, 126.9, 130.3, 156.2, 173.6.

2-(3,4-Dimethoxyphenyl)acetamide(3aa)

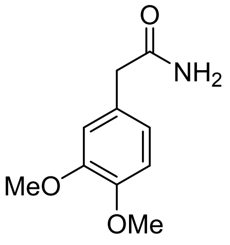

yield: 93 mg (86%); brown solid; mp: 140-144°C; **^{1}H NMR** (400 MHz, *DMSO-d$_6$*): δ 3.28 (s, 2H), 3.72 (s, 3H), 3.71 (s, 3H), 6.74 - 6.79 (m, 1H), 6.80 - 6.89 (m, 3H), 7.37 (br. s., 1H); **^{13}C NMR** (101 MHz, *DMSO-d$_6$*): δ 42.5, 56.1, 56.2, 112.4 , 113.6, 121.7, 129.6, 148.1, 149.1, 173.2; **HRMS** (ESI) calculated $[M+H]^+$ for $C_{10}H_{14}O_3N$: 196.0968; found: 196.0969

2-(3,4-Dichlorophenyl)acetamide(3ab)

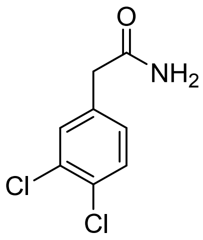

yield: 78 mg (74%); brown solid; **^{1}H NMR** (400 MHz, *DMSO-d$_6$*): δ 3.36 - 3.46 (m, 2H), 7.34 (br. s., 1H), 7.41 (br. s., 2H), 7.56 - 7.67 (m, 2H), 7.88 (br. s., 1H); **^{13}C NMR** (101 MHz, *DMSO-d$_6$*): δ 39, 118.7, 127.6, 128.6, 130.7, 132.8, 139.3, 169.2; **HRMS** (ESI) calculated $[M+H]^+$ for $C_8H_8ONCl_2$: 203.9977; found: 203.9980

Octanamide(3ac)

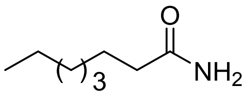

yield: 47 mg; (42%); white solid; mp: 107- 109°C; **^{1}H NMR** (400 MHz, $CDCl_3$): δ 0.82 - 0.95 (m, 3H), 1.30 (s, 4H), 1.27 (s, 5H), 1.56 - 1.70 (m, 2H), 2.21 (t, J = 7.6 Hz, 2 H), 5.54 (br. s., 1H), 5.86 (br. s., 1H); **^{13}C NMR** (101 MHz, $CDCl_3$): δ 14.0, 22.5, 25.5, 29.0, 29.1, 31.6, 35.9, 175.9.

Decanamide(3ad)

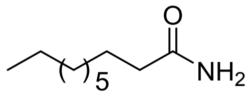

yield: 42 mg (39%); white solid; mp: 101- 103°C; **^{1}H NMR** (400 MHz, *DMSO-d_6*): δ 0.85 (t, *J* = 6.4 Hz, 3H), 1.24 (s, 13H), 1.42 - 1.51 (m, 2H), 2.01 (t, *J* = 7.3 Hz, 2H), 6.66 (br. s., 1H), 7.21 (br. s., 1H); **^{13}C NMR** (101 MHz, *DMSO-d_6*): δ 14.0, 22.1, 25.1, 28.7, 28.7, 28.9, 29.0, 31.3, 35.1, 174.4; **HRMS** (ESI) calculated $[M+H]^+$ for $C_{10}H_{12}ON$: 172.1696; found: 172.1696.

Thiophene-2-carboxamide(3ae)

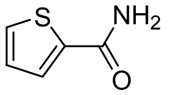

yield: 68 mg (61%); white solid; mp:181- 183°C; **^{1}H NMR** (400 MHz, *DMSO-d_6*): δ 7.12 (br. s., 1H), 7.39 (br. s., 1H), 7.75 (s, 1H), 7.71 (s, 1H), 7.99 (br. s., 1H); **^{13}C NMR** (101 MHz, *DMSO-d_6*): δ 128.4, 129.2, 131.4, 140.7, 163.5; **HRMS** (ESI) calculated $[M+H]^+$ for C_5H_6ONS: 128.0165; found: 128.0165.

2-Picolinamide (3f)

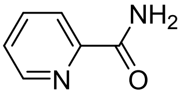

yield: 28 mg (25%); white solid; mp: 104- 108°C; **^{1}H NMR** (400 MHz, *DMSO-d_6*): δ 7.52 - 7.59 (m, 1H), 7.71 (br. s., 1H), 7.92 - 8.00 (m, 1H), 8.06 (d, *J* = 7.8 Hz, 1H), 8.17 (br. s., 1H), 8.61 (d, *J* = 2.7 Hz, 1H); **^{13}C NMR** (101 MHz, *DMSO-d_6*): δ 122.4, 126.9, 138.1, 148.9, 150.7 , 166.6; **HRMS** (ESI) calculated $[M+H]^+$ for $C_6H_7ON_2$:

123.0553; found: 123.0555

2-Benzoylbenzo[d]isothiazol-3(2H)-one-1,1-dioxide (8)

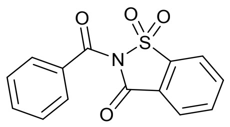

white solid**;** ^{1}**H NMR** (400 MHz, *DMSO-d*$_6$) δ ppm 7.3 - 7.5 (m, 2H), 7.6 - 7.6 (m, 1H), 7.6 - 7.8 (m, 1H), 7.8 - 8.1 (m, 5H), 8.2 (d, *J* = 7.33 Hz, 1H). ^{13}**C NMR** (101 MHz, *DMSO-d*$_6$): δ 121, 125, 127, 129, 131, 133, 135 , 136, 139, 161, 167.

Moclobemide (7)

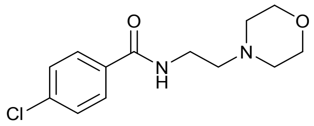

yield: 5.35 gm (62%); white solid; mp: 135- 137°C; ^{1}**H NMR** (500 MHz, $CDCl_3$): δ 2 (br. s., 4H), 3 (t, *J* = 5.91 Hz, 2H), 3 - 4 (m, 2H), 4 (t, *J* = 4.58 Hz, 4H), 7 (br. s., 1H), 7 (m, *J* = 8.77 Hz, 2H), 8 - 8 (m, 2H); ^{13}**C NMR** (126 MHz, $CDCl_3$) : δ 36, 53, 57, 67, 128,128, 128, 133, 137, 166.

Spectral Data

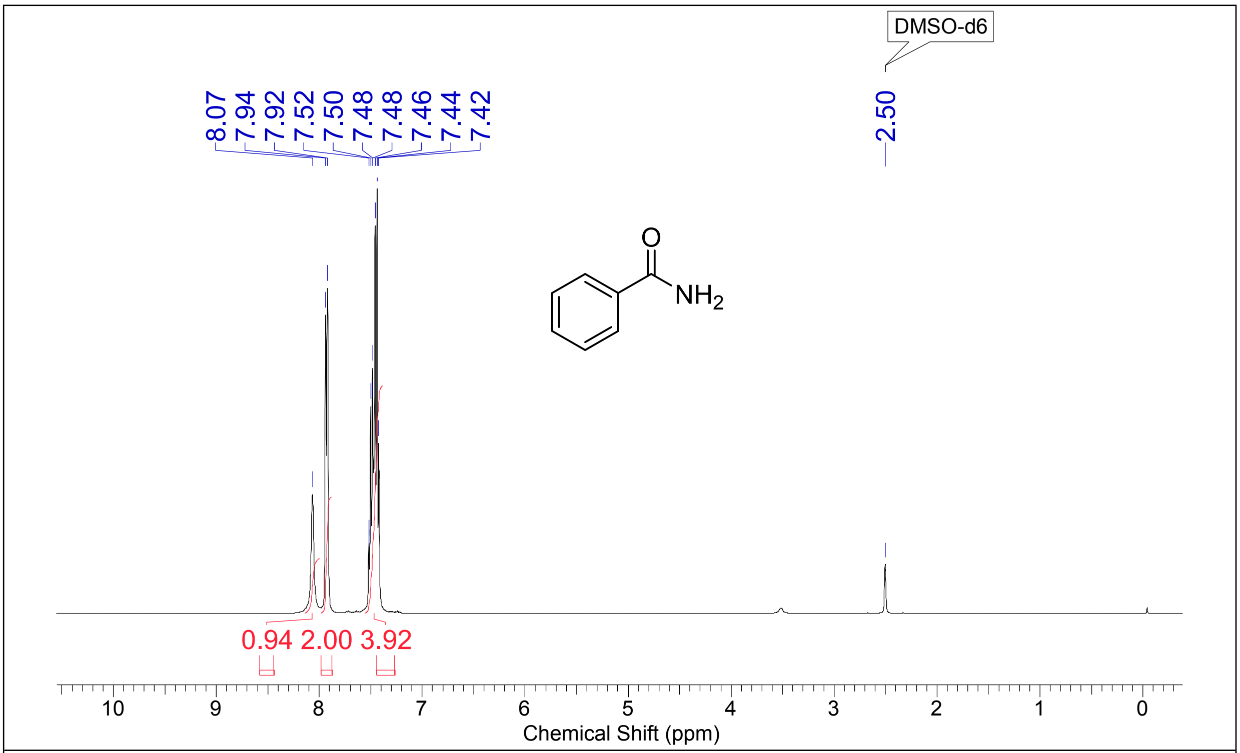

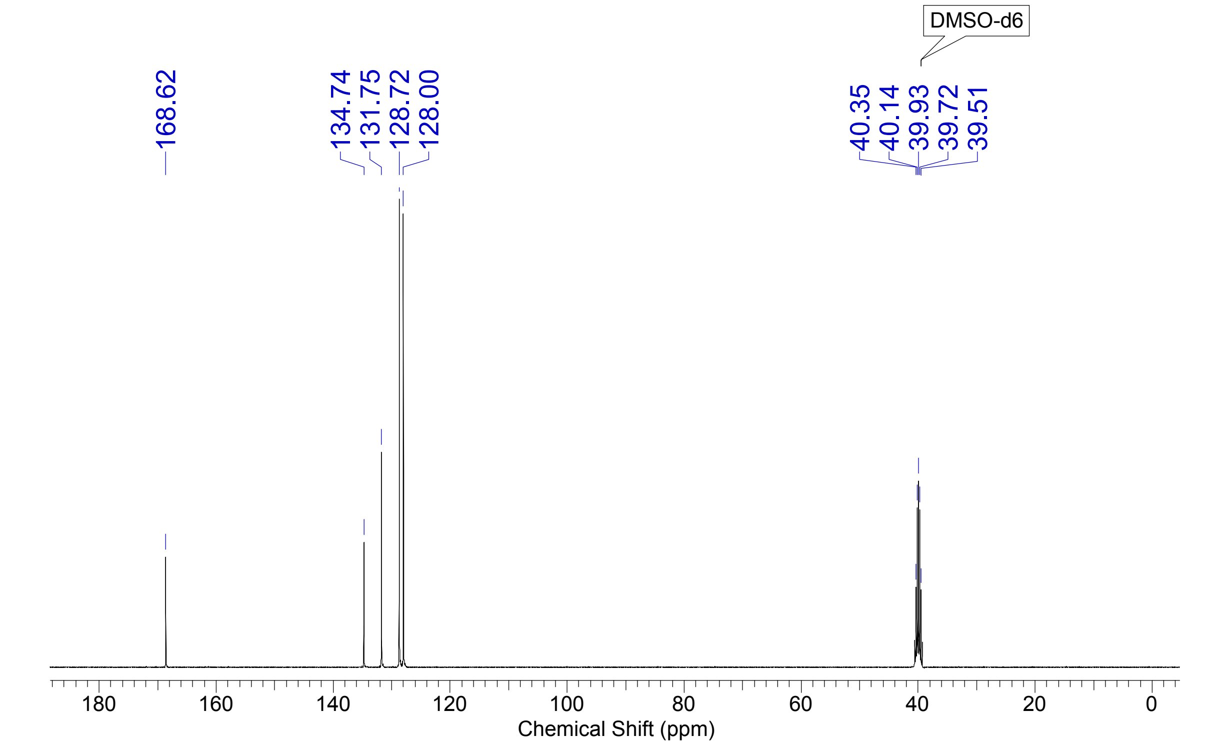

benzamide (3a)

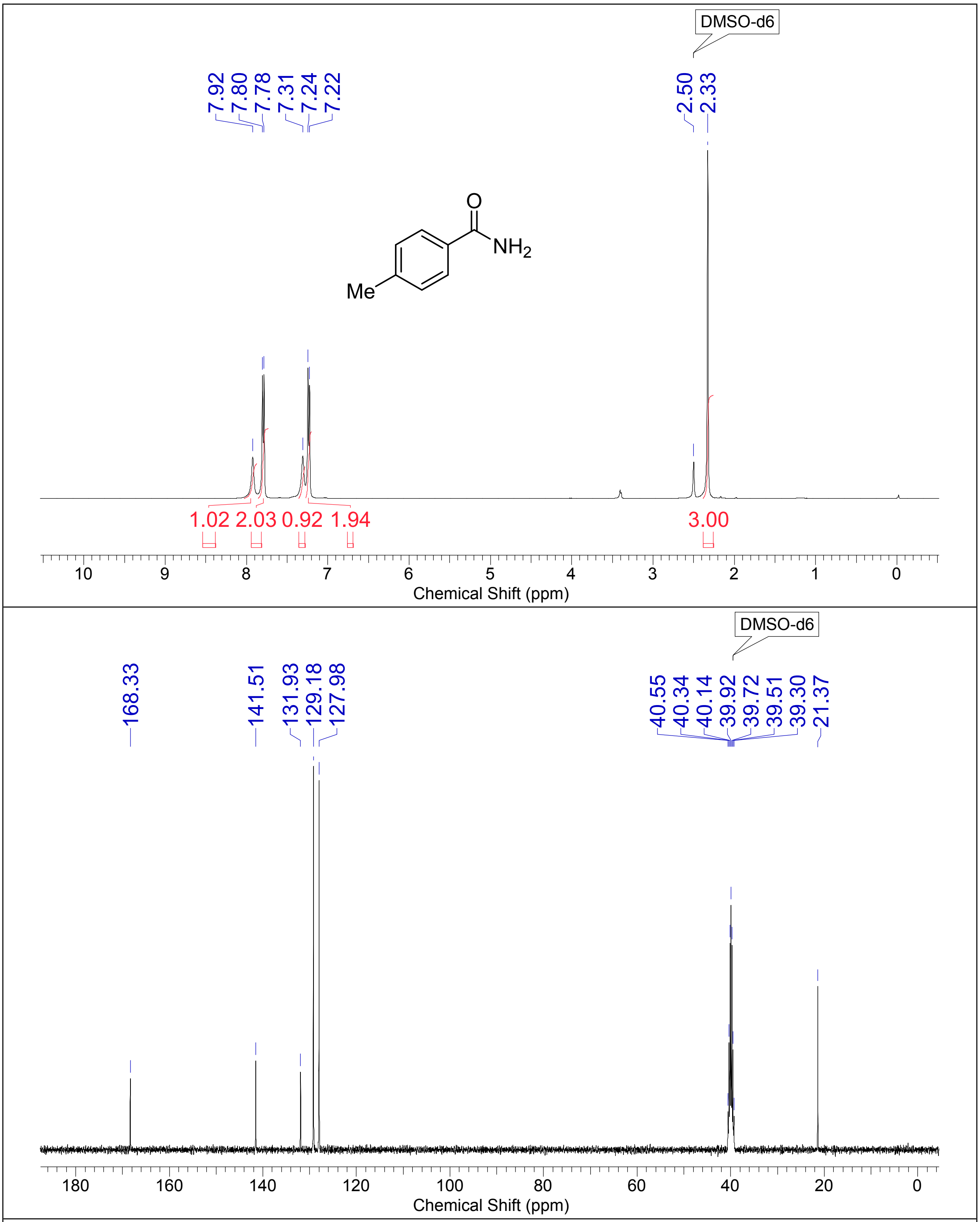

4-methylbenzamide(3b)

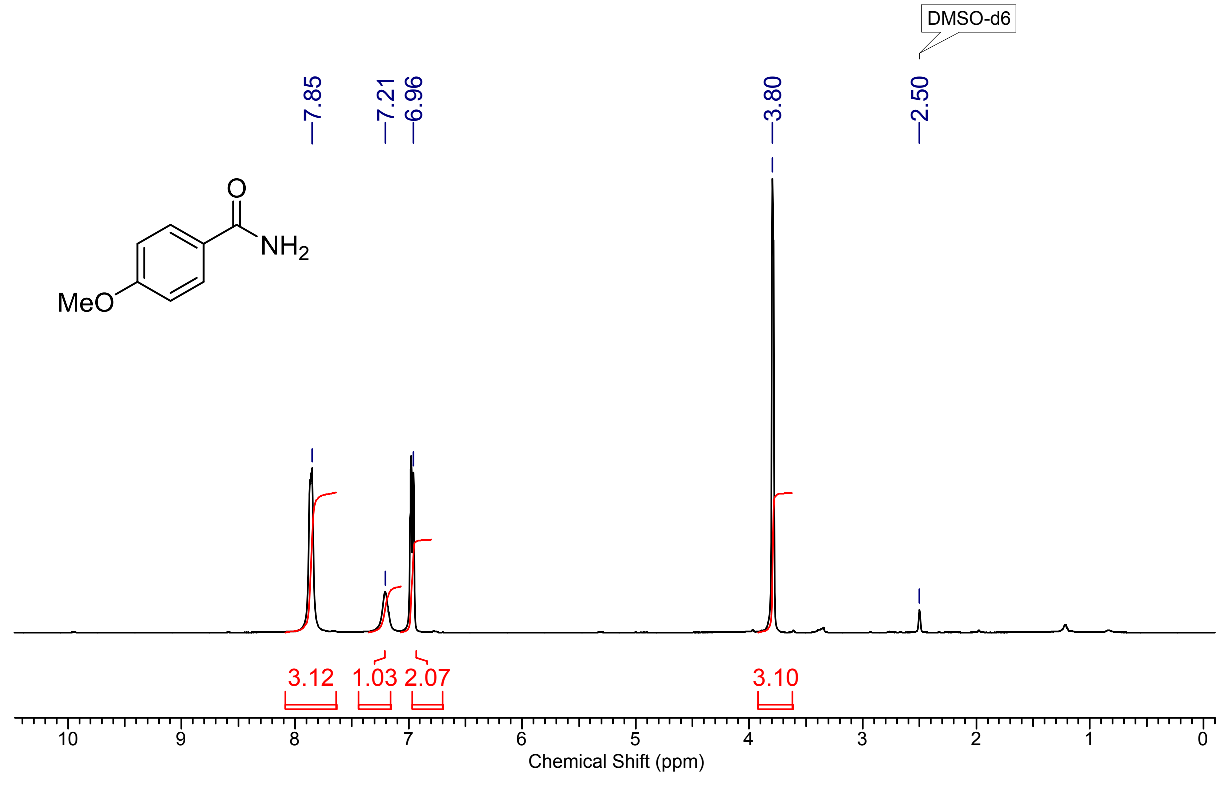

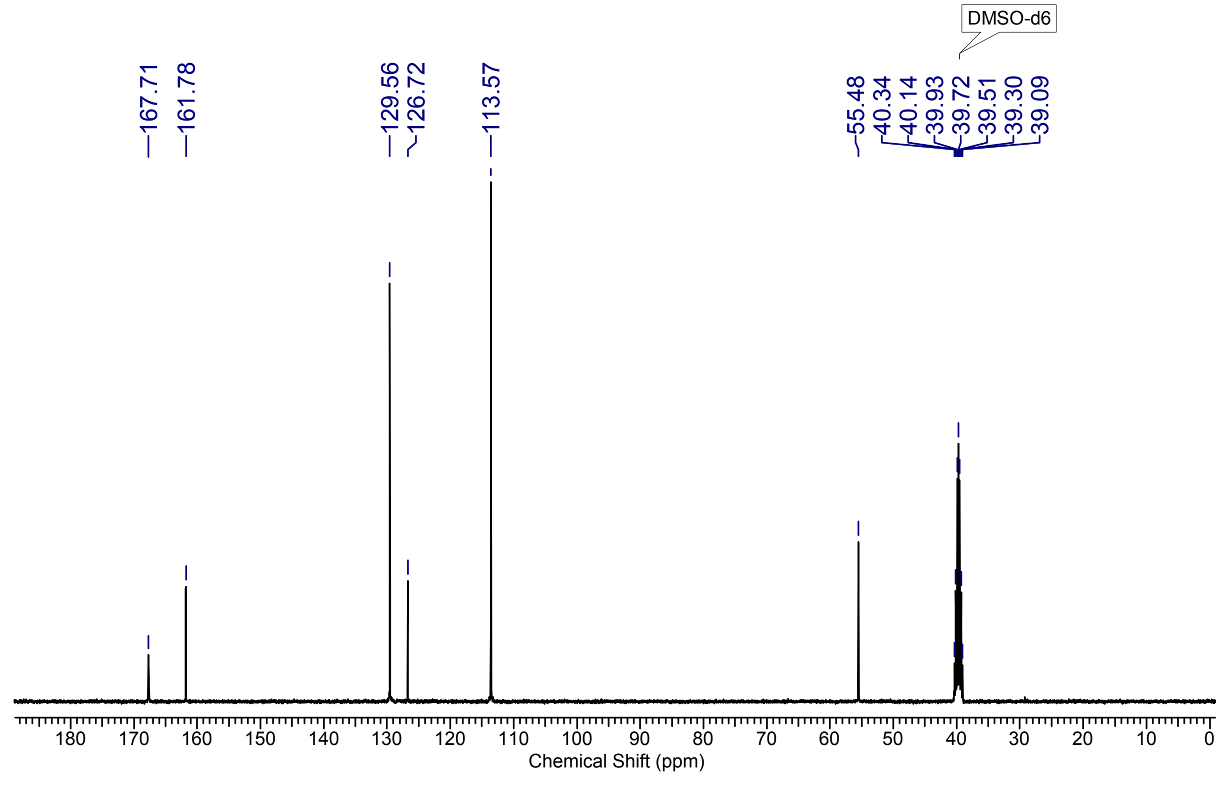

4-methoxybenzamide(3c)

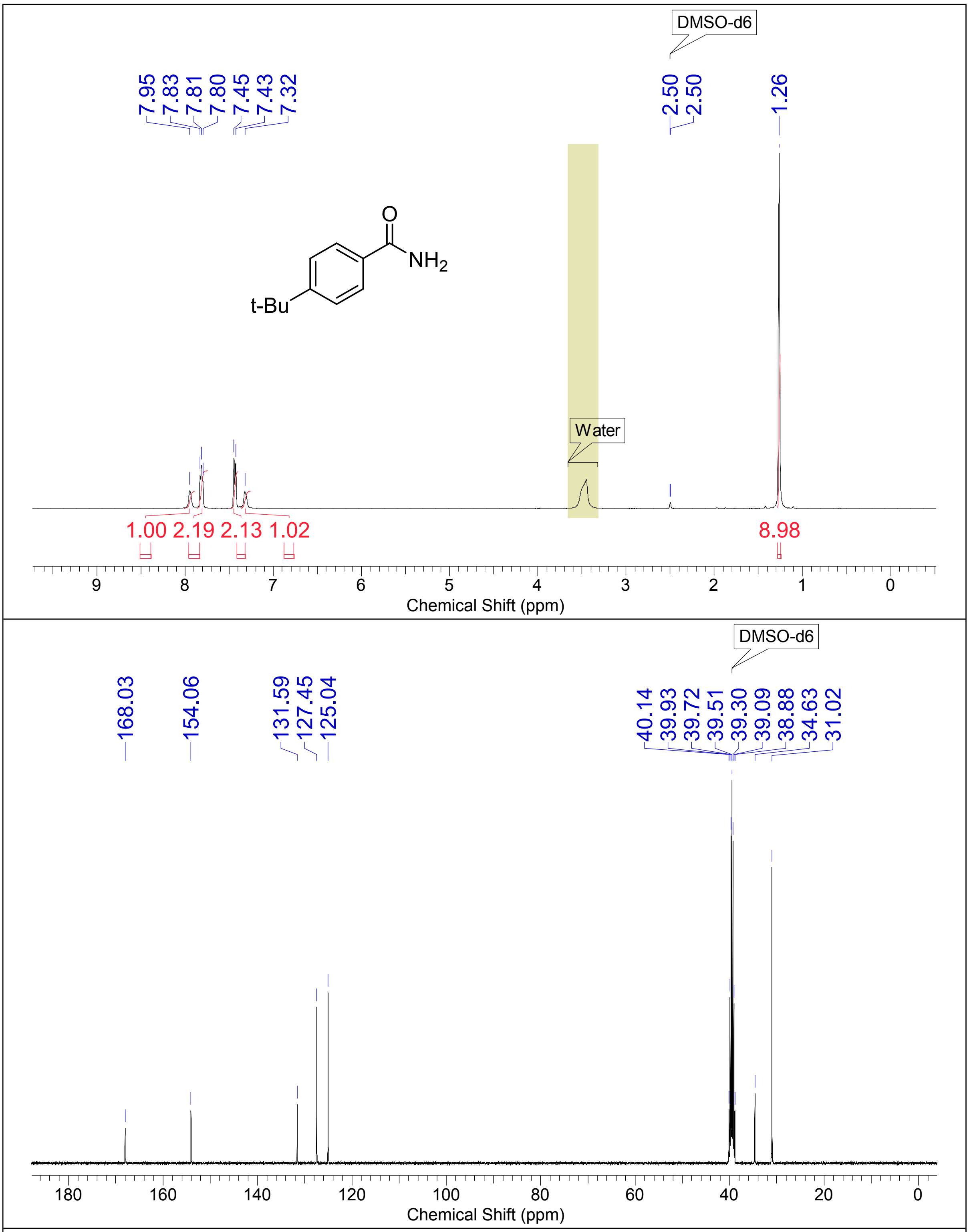

4-(tert-butyl)benzamide(3d)

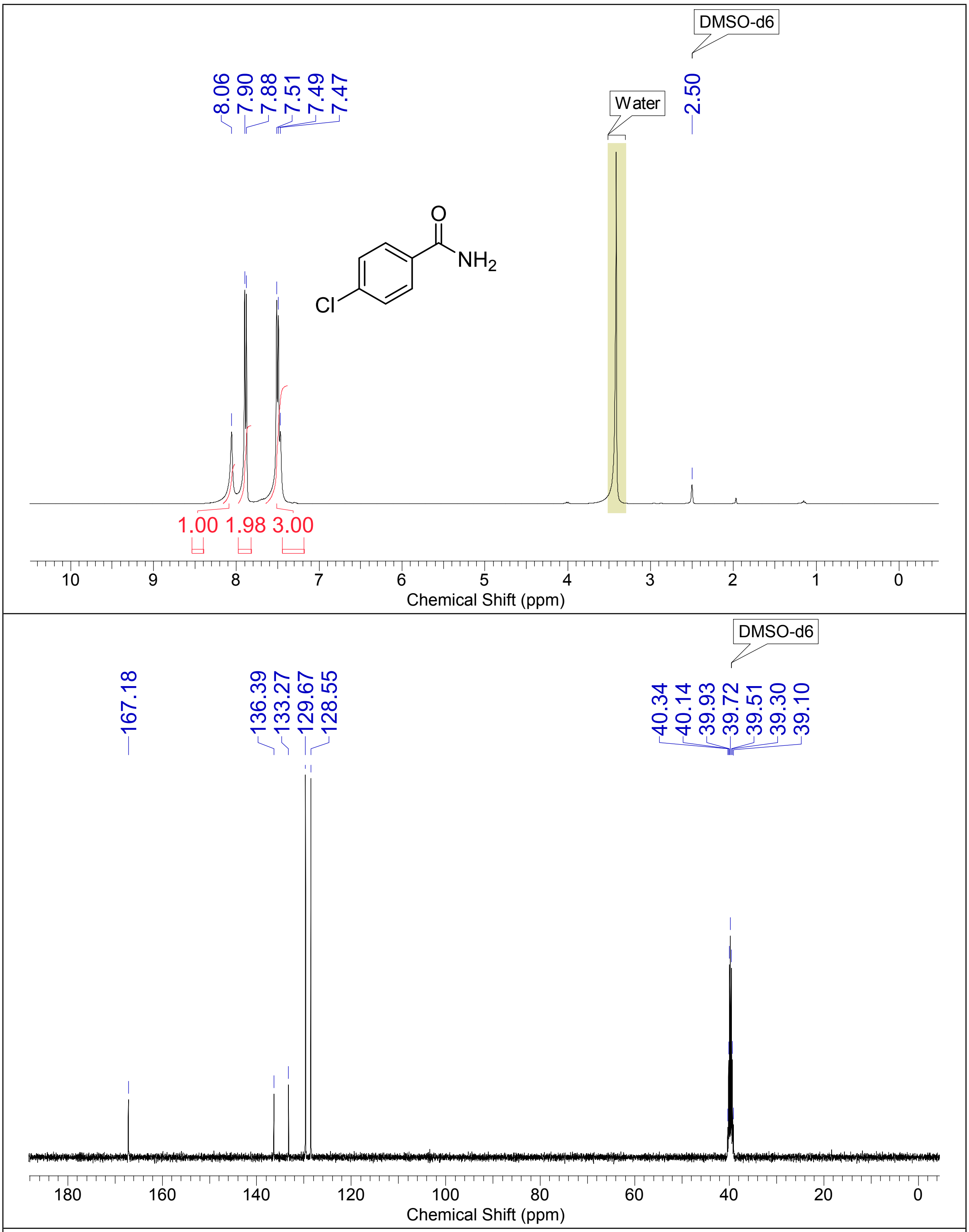

4-chlorobenzamide(3e)

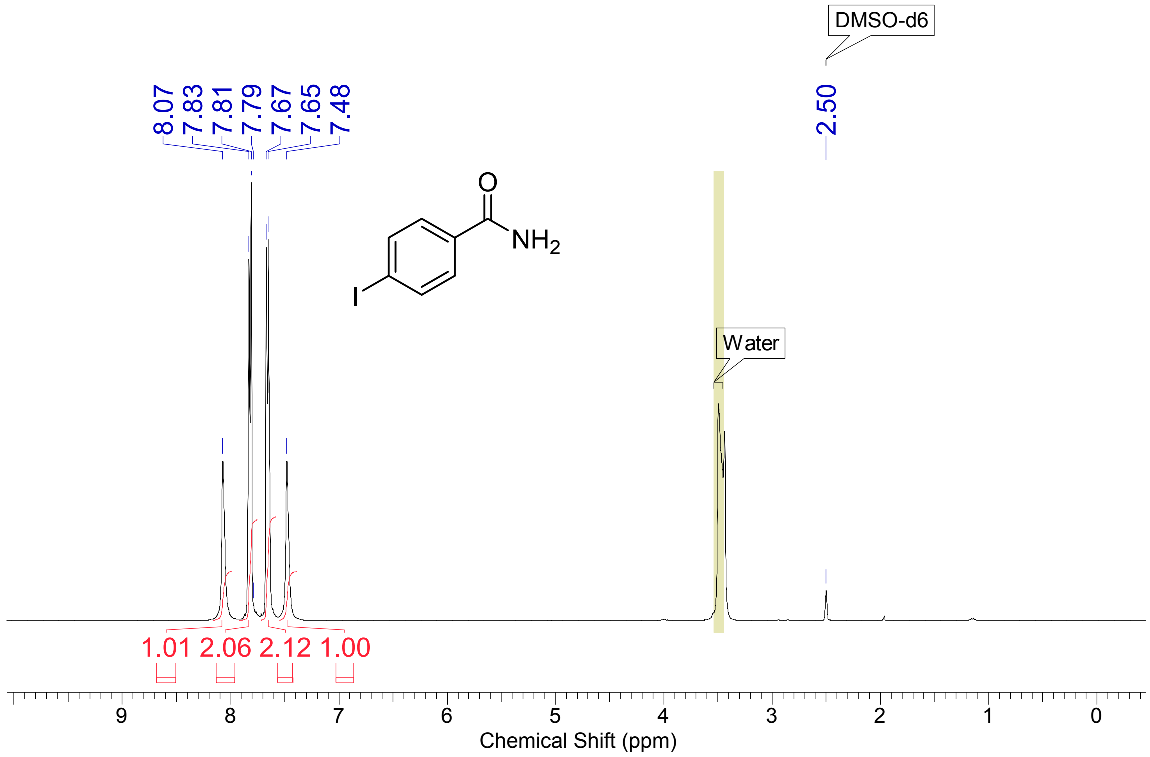

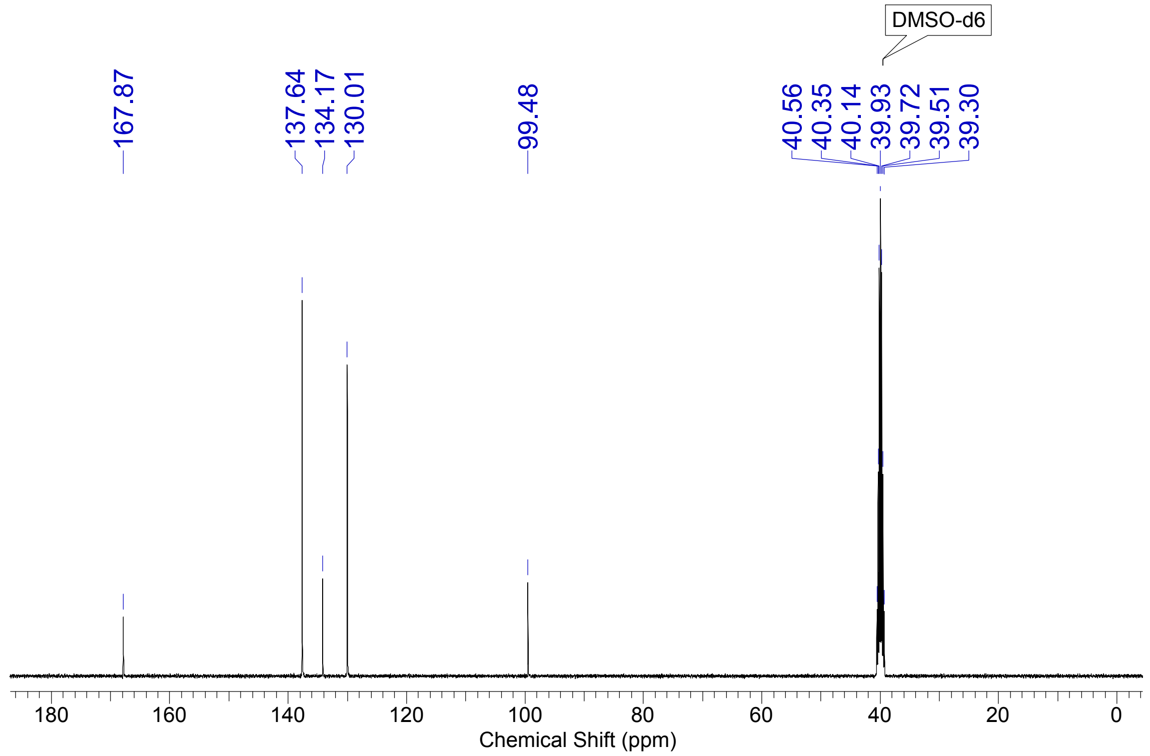

4-iodobenzamide(3f)

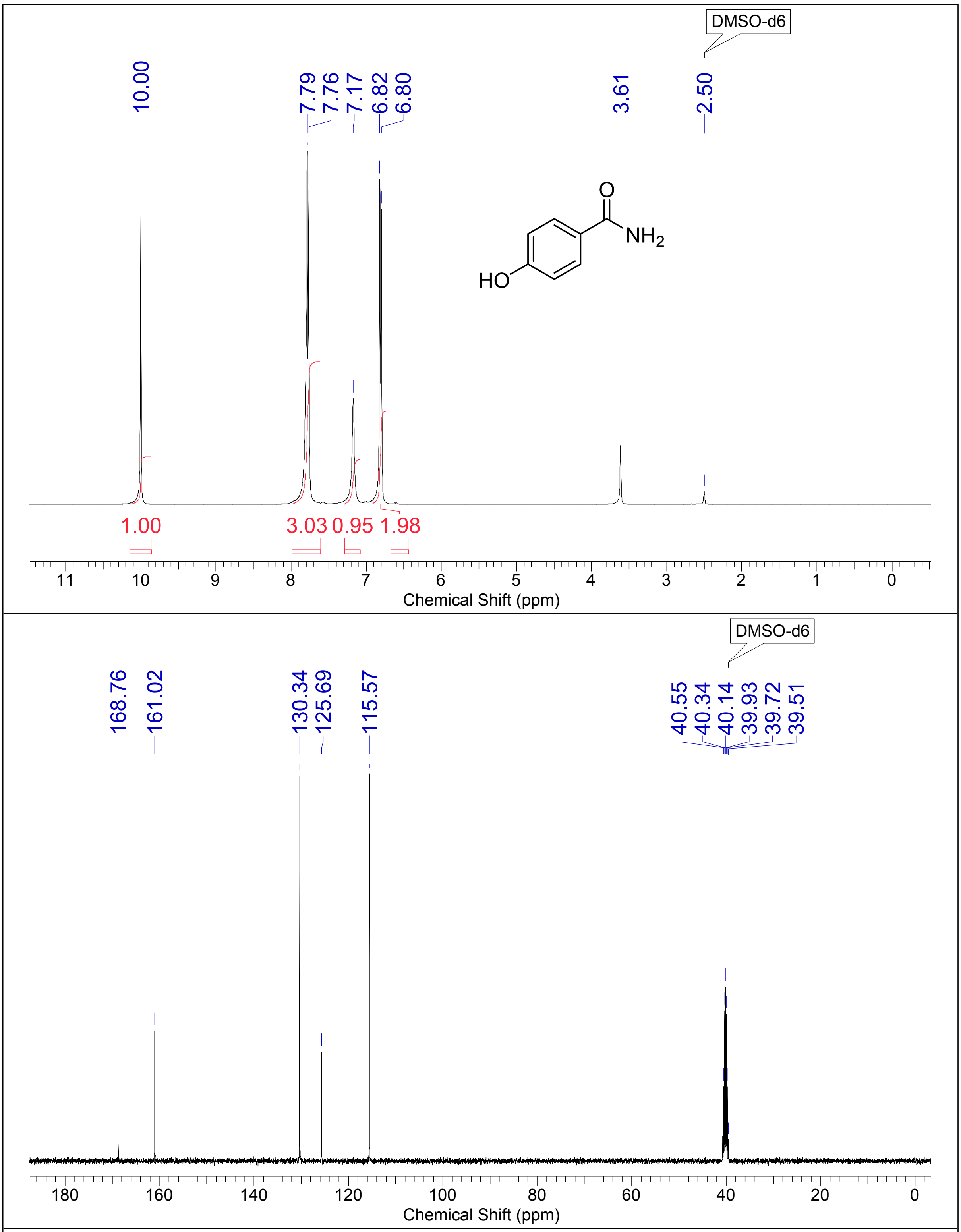

4-hydroxybenzamide(3g)

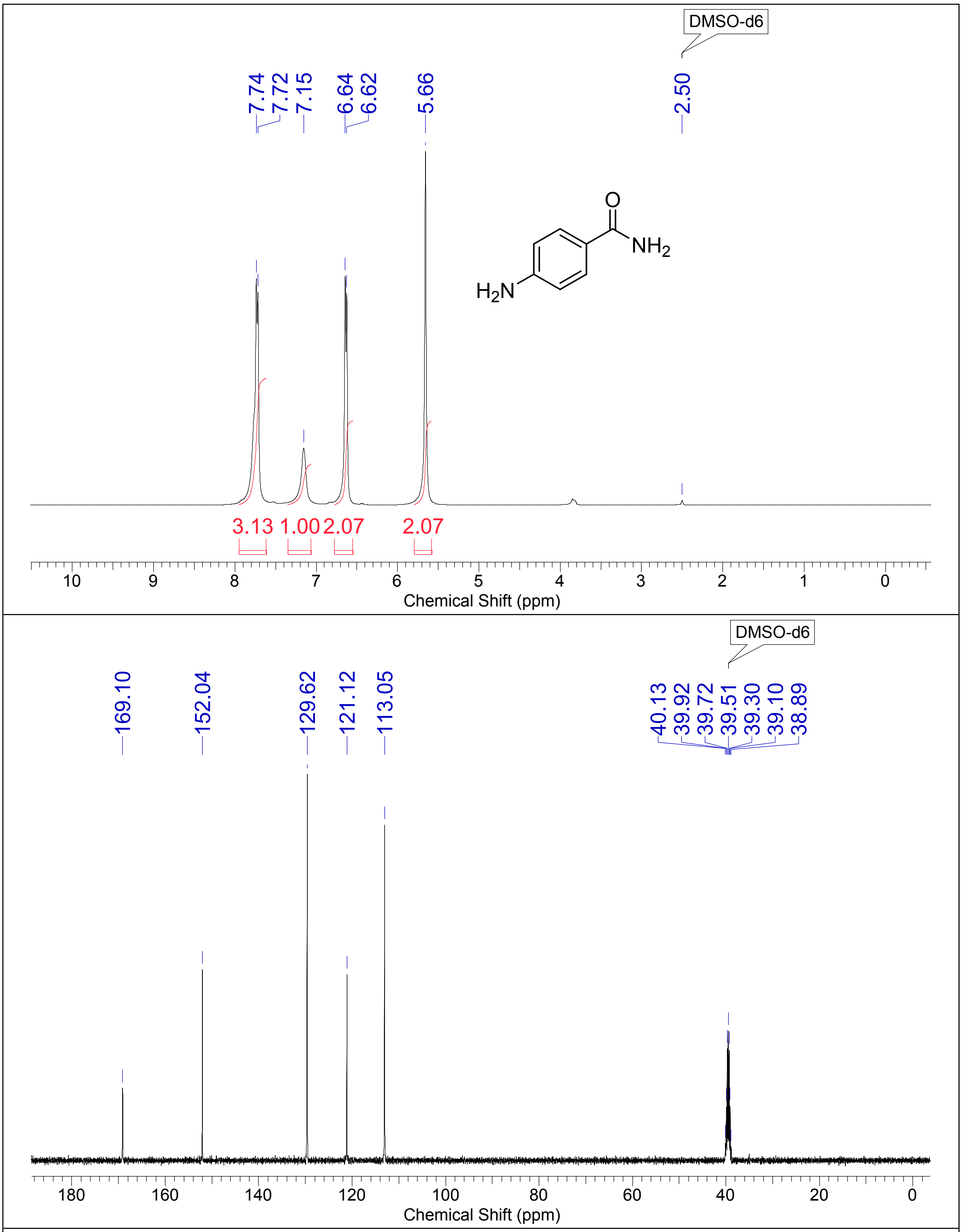

4-aminobenzamide(3h)

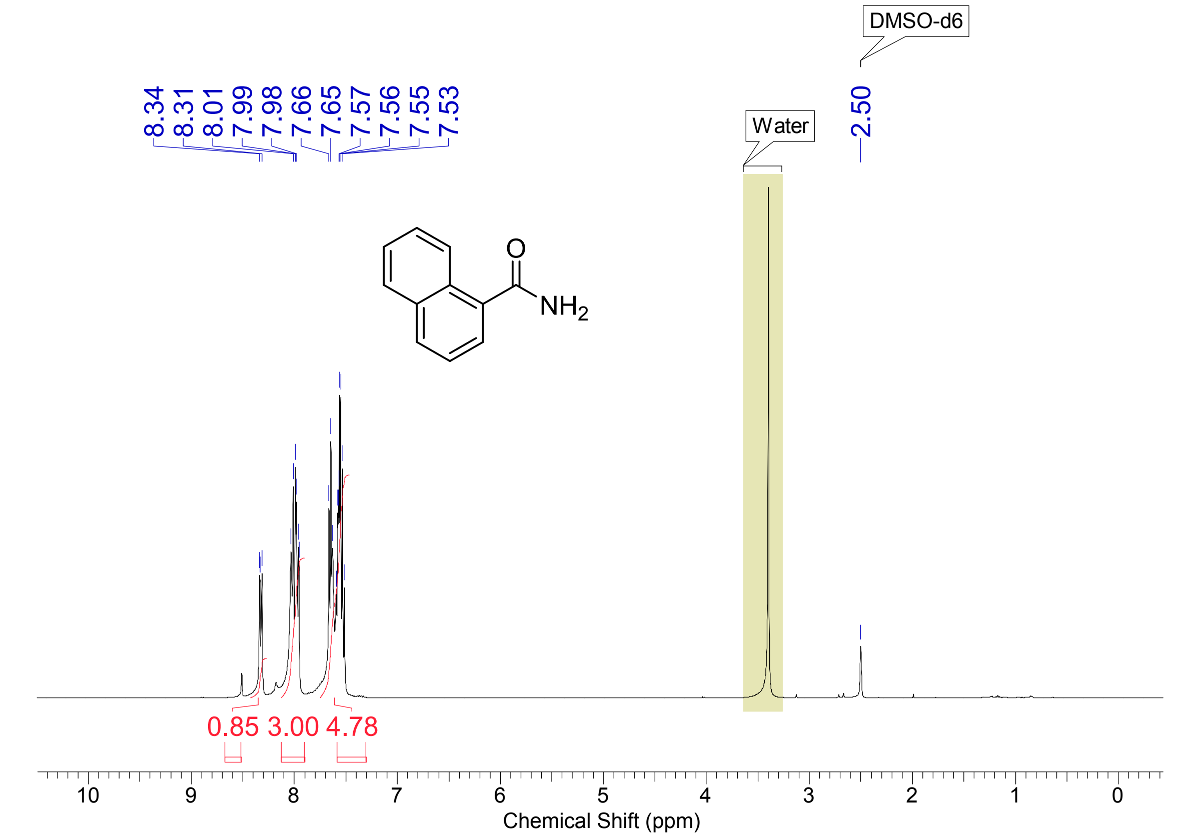

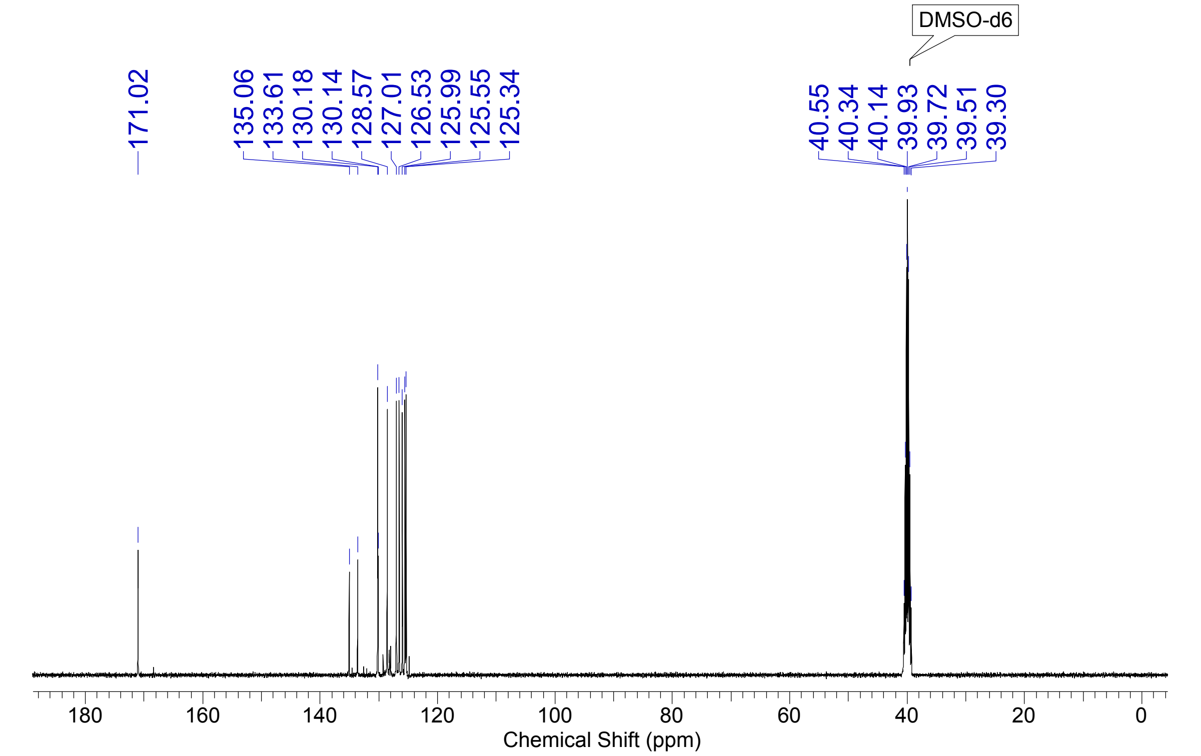

1-naphthamide(3i)

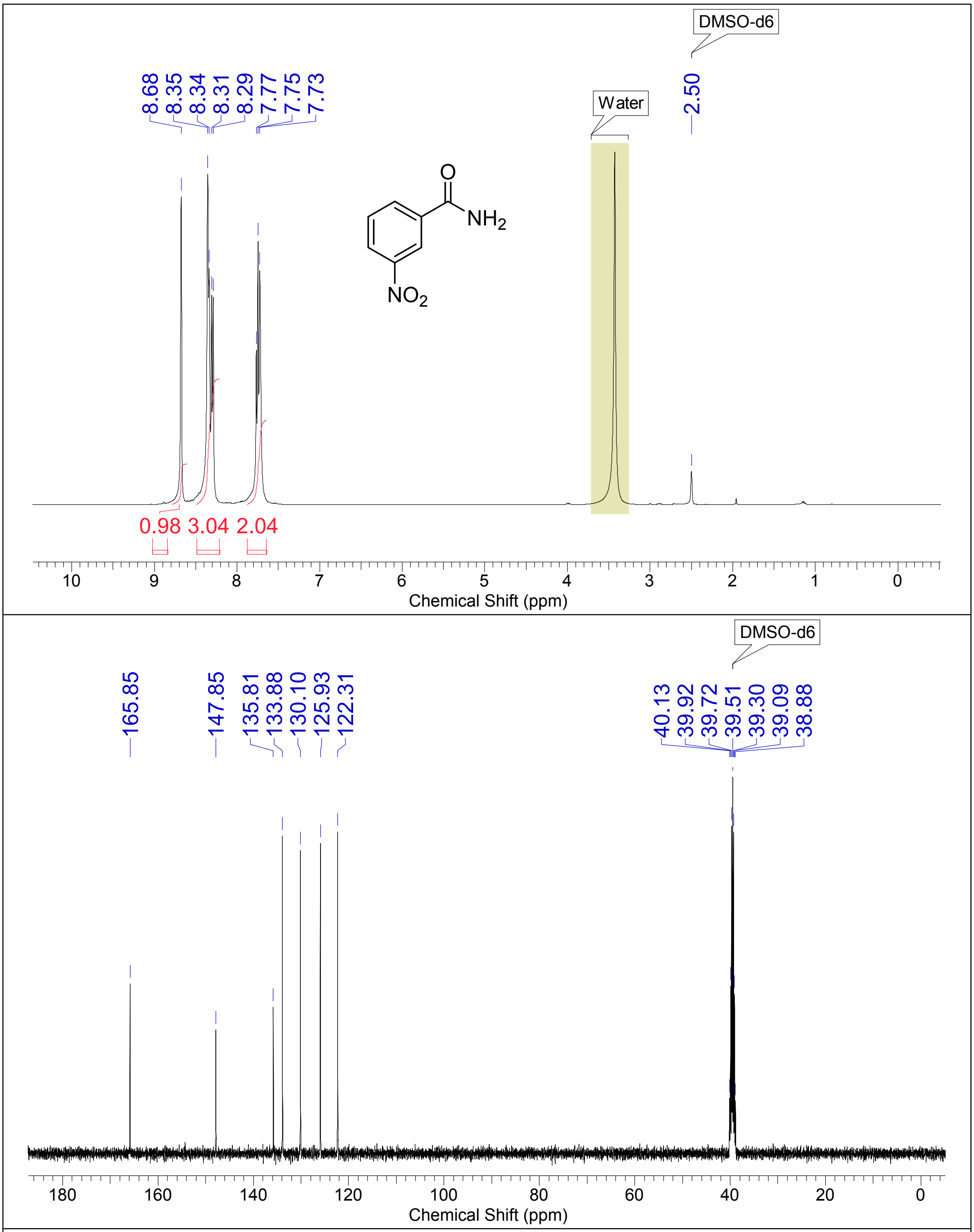

3-nitrobenzamide(3j)

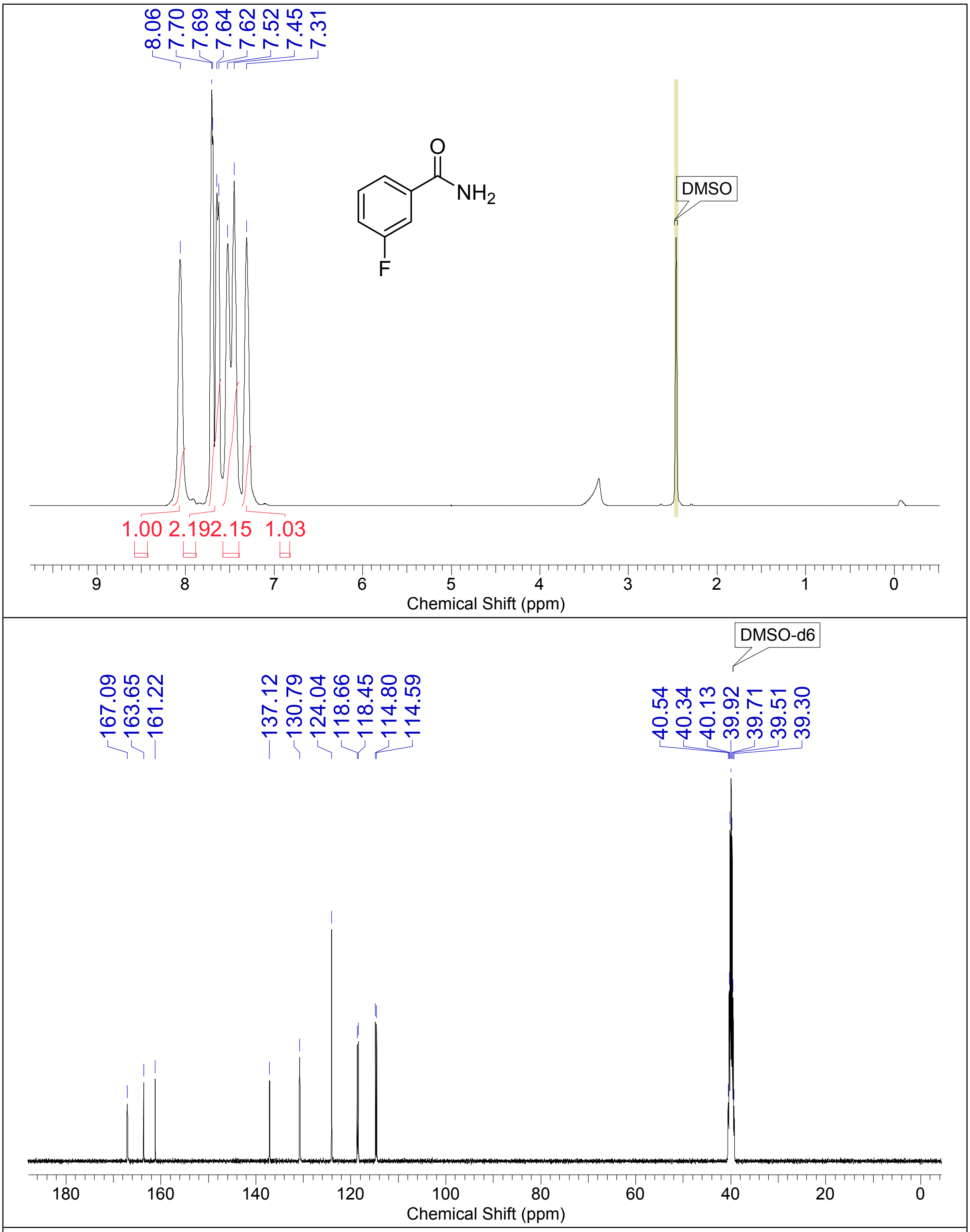

3-fluorobenzamide(3k)

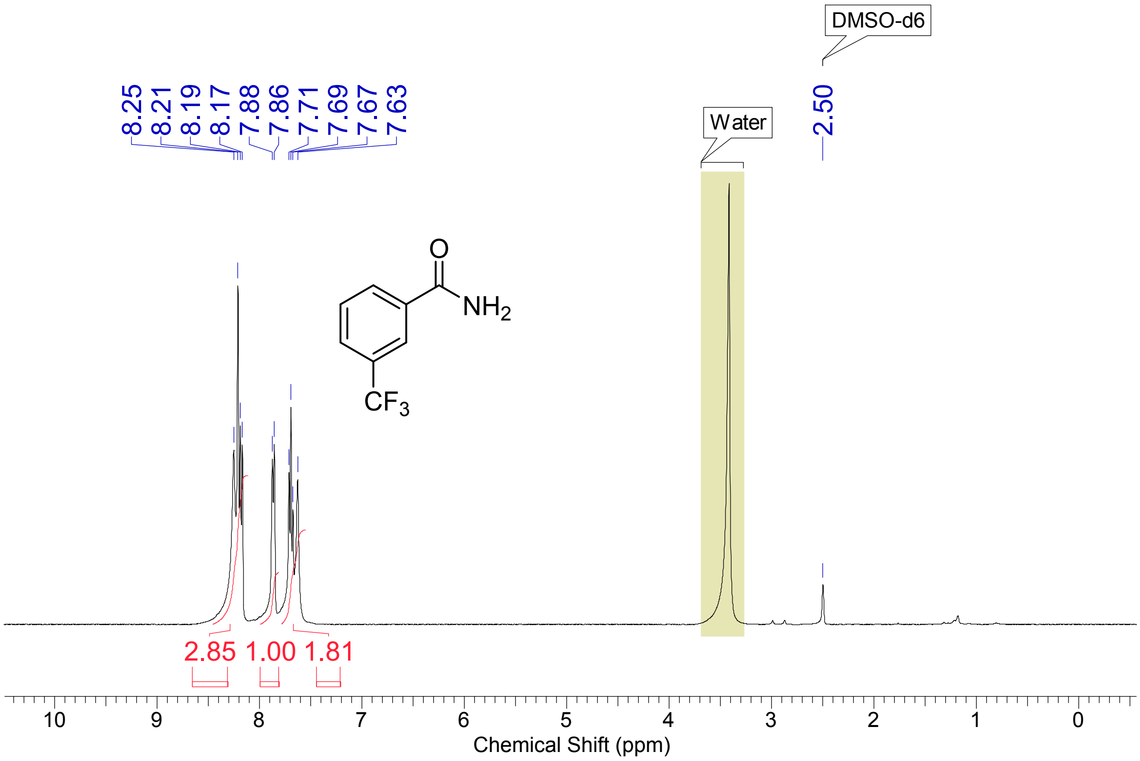

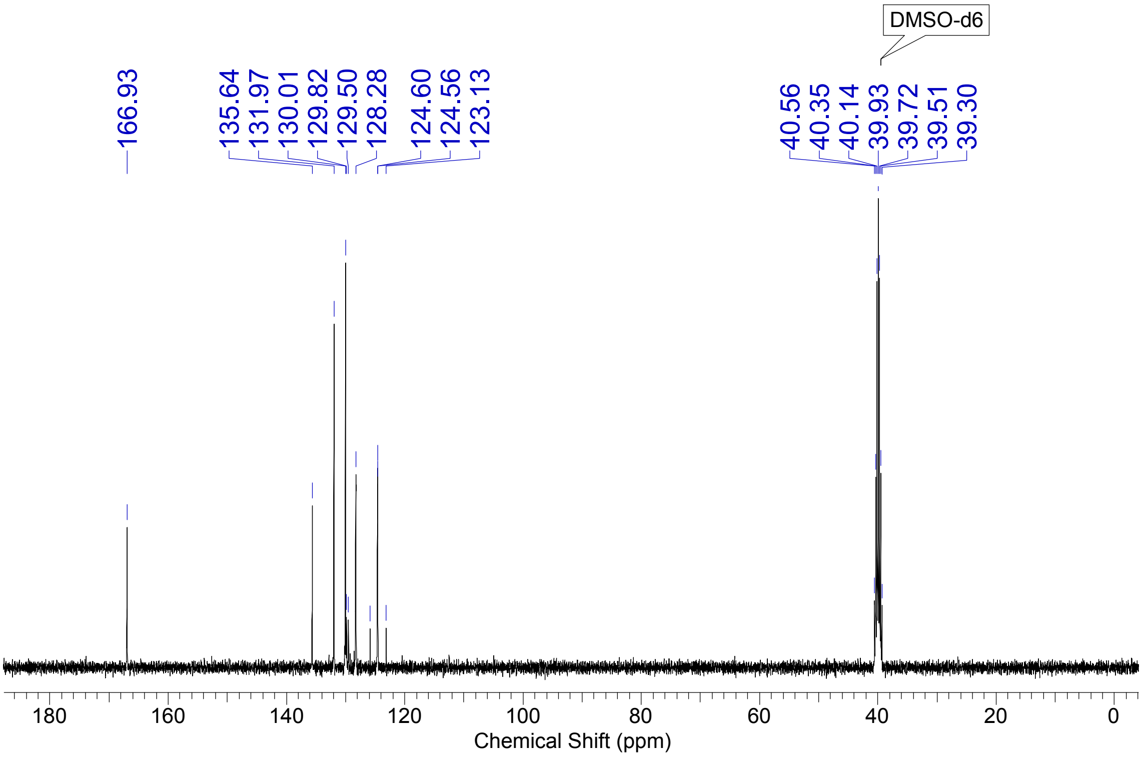

3-(trifluoromethyl)benzamide(3l)

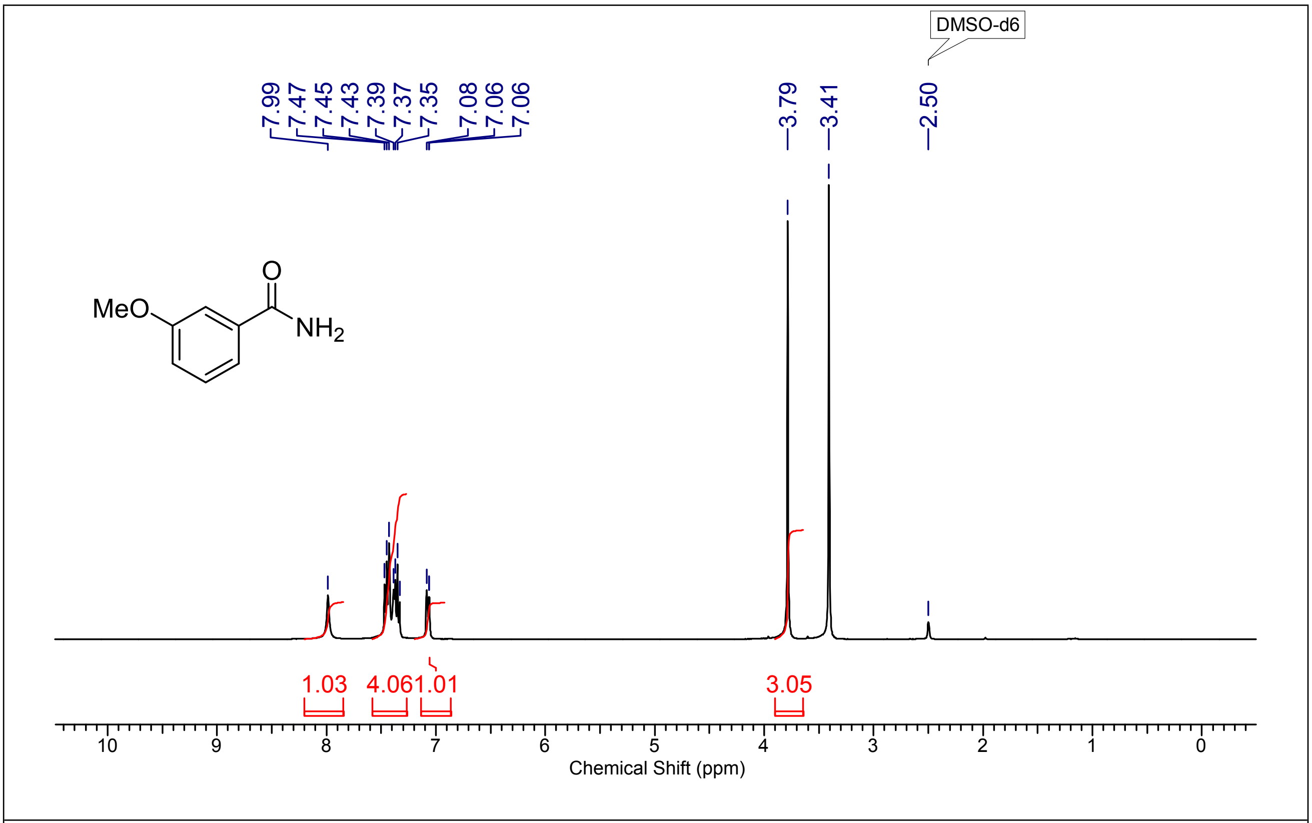

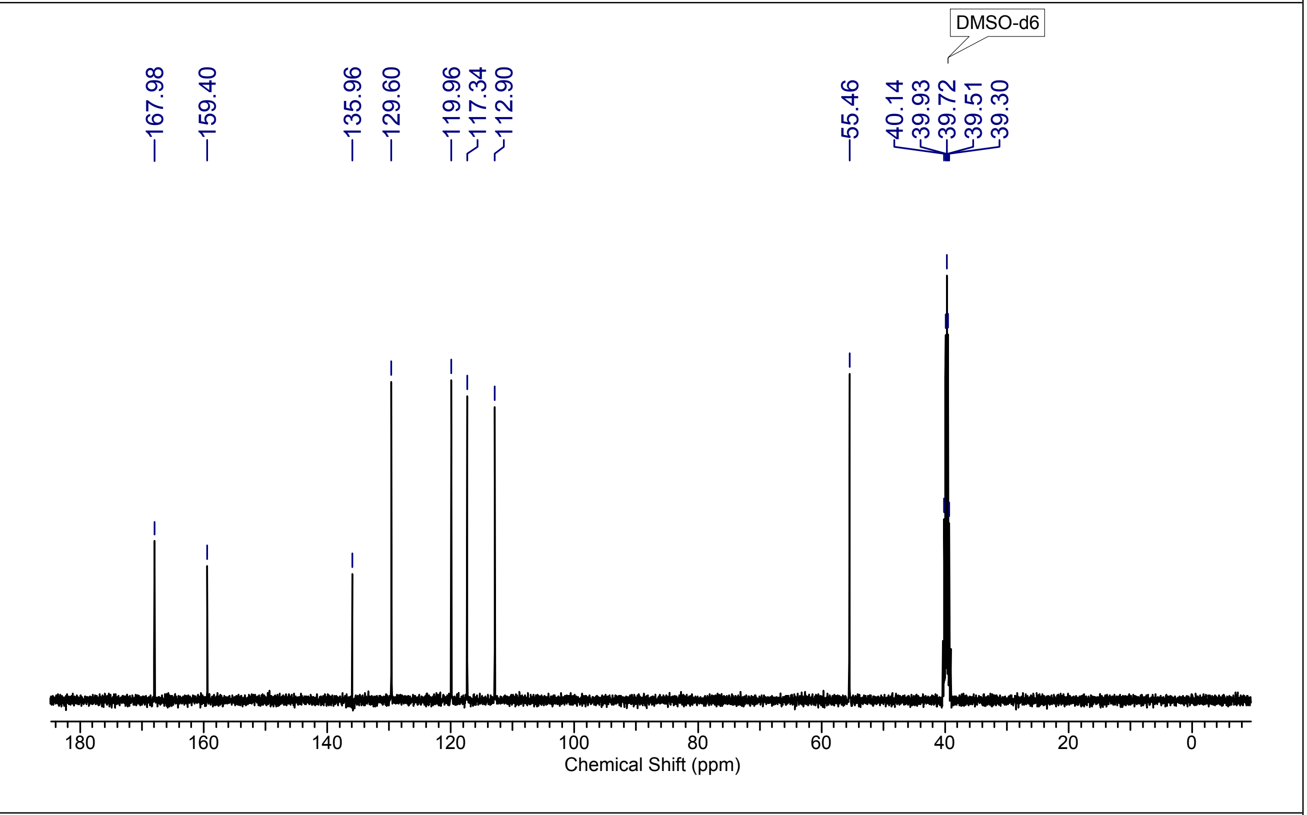

3-methoxybenzamide(3m)

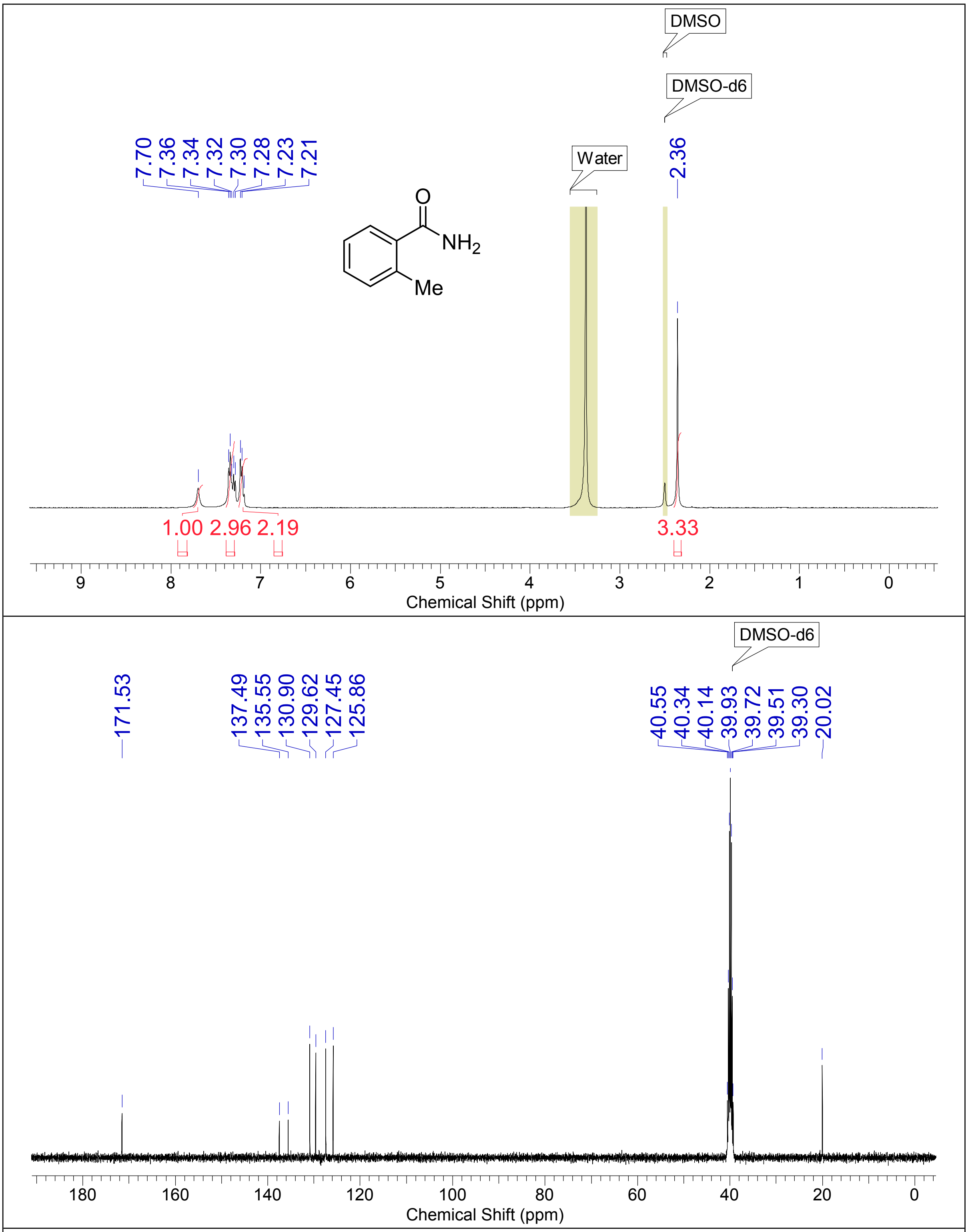

2-methylbenzamide(3n)

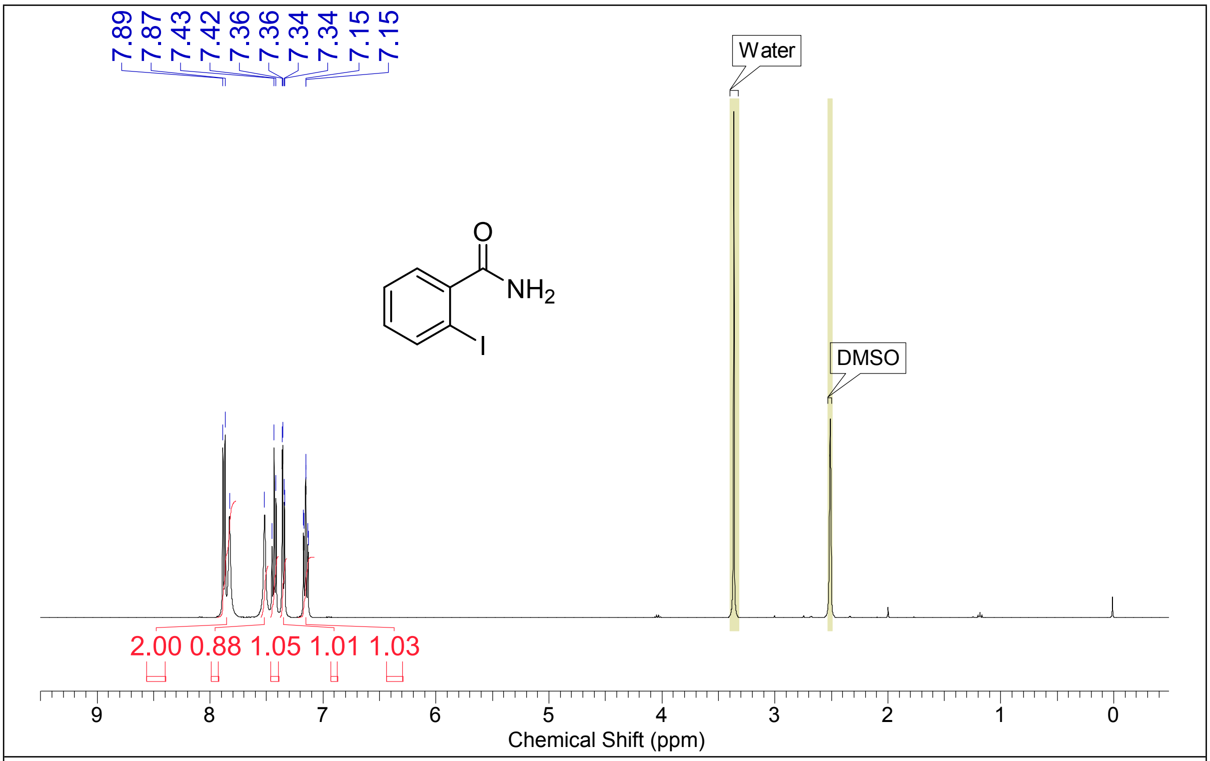

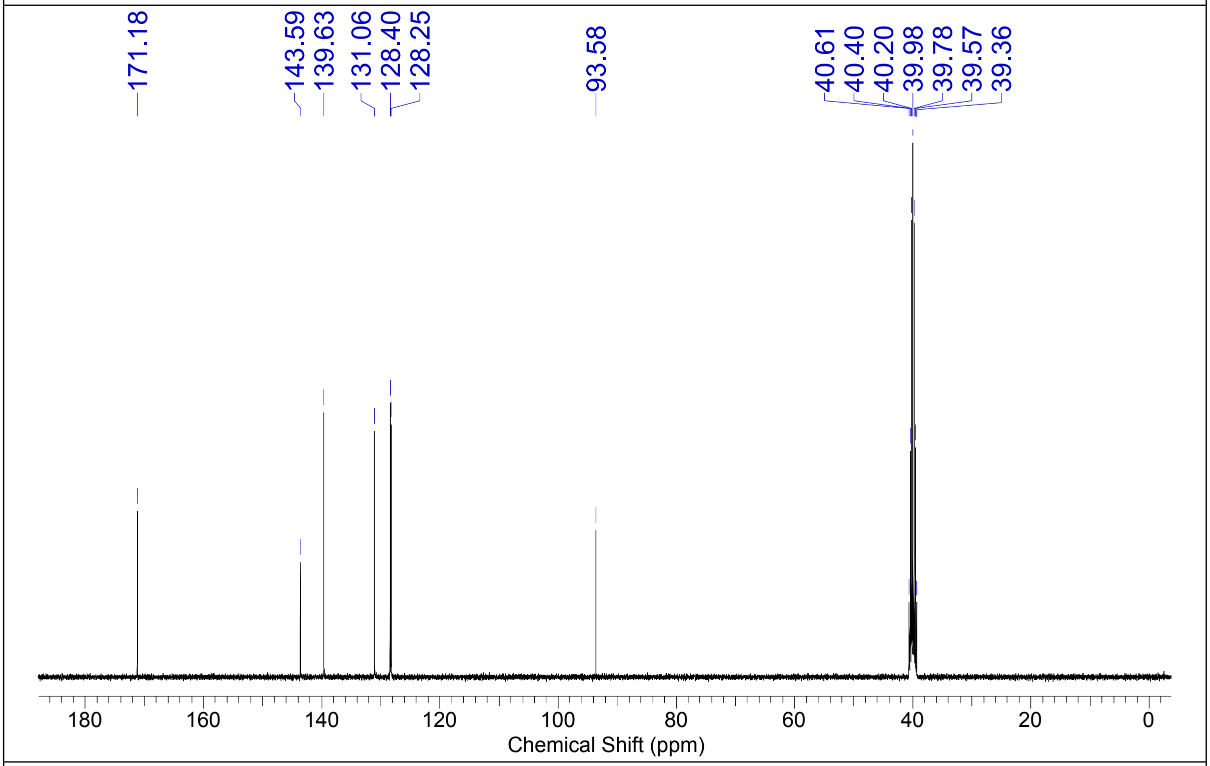

2-iodobenzamide(3o)

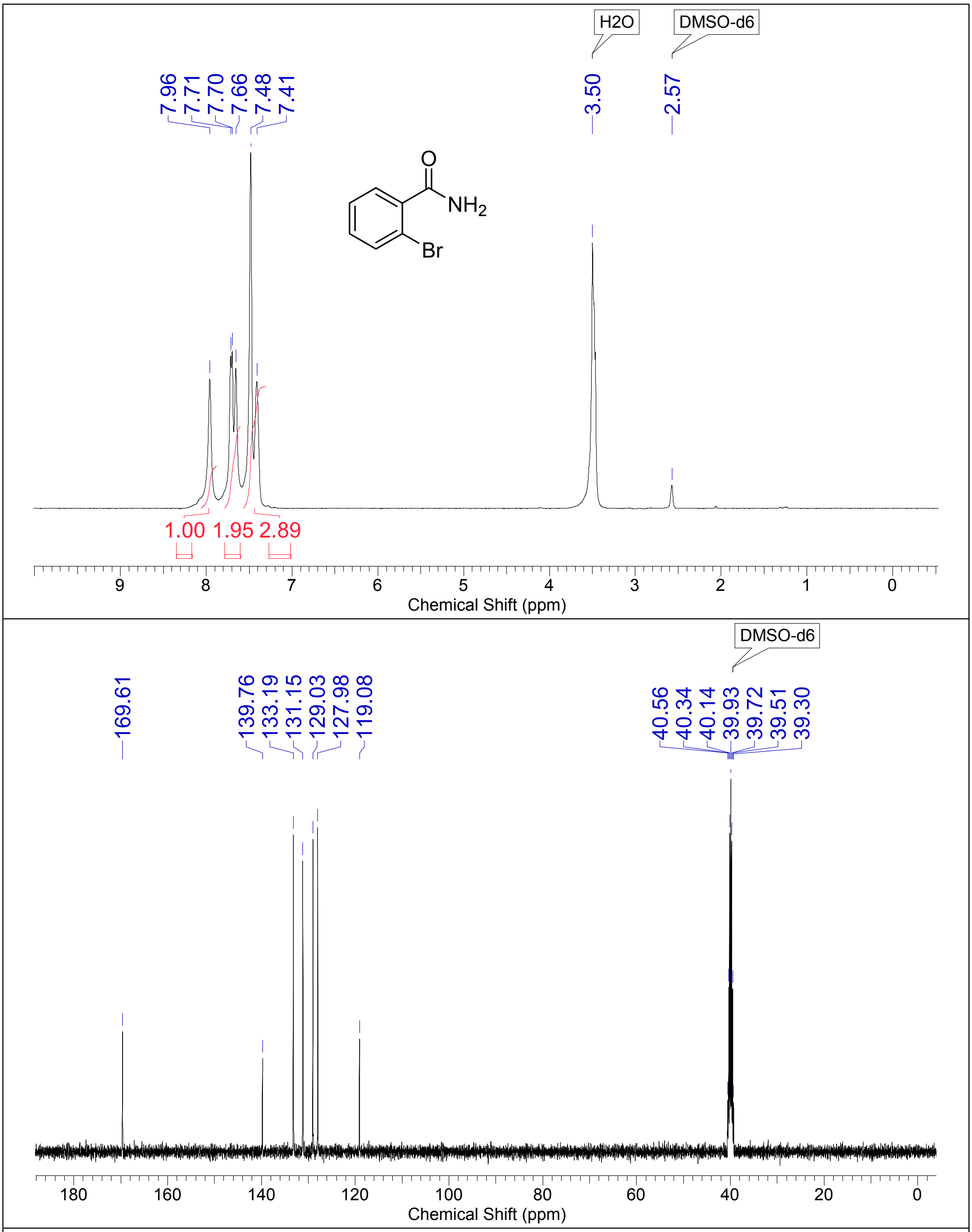

2-bromobenzamide(3p)

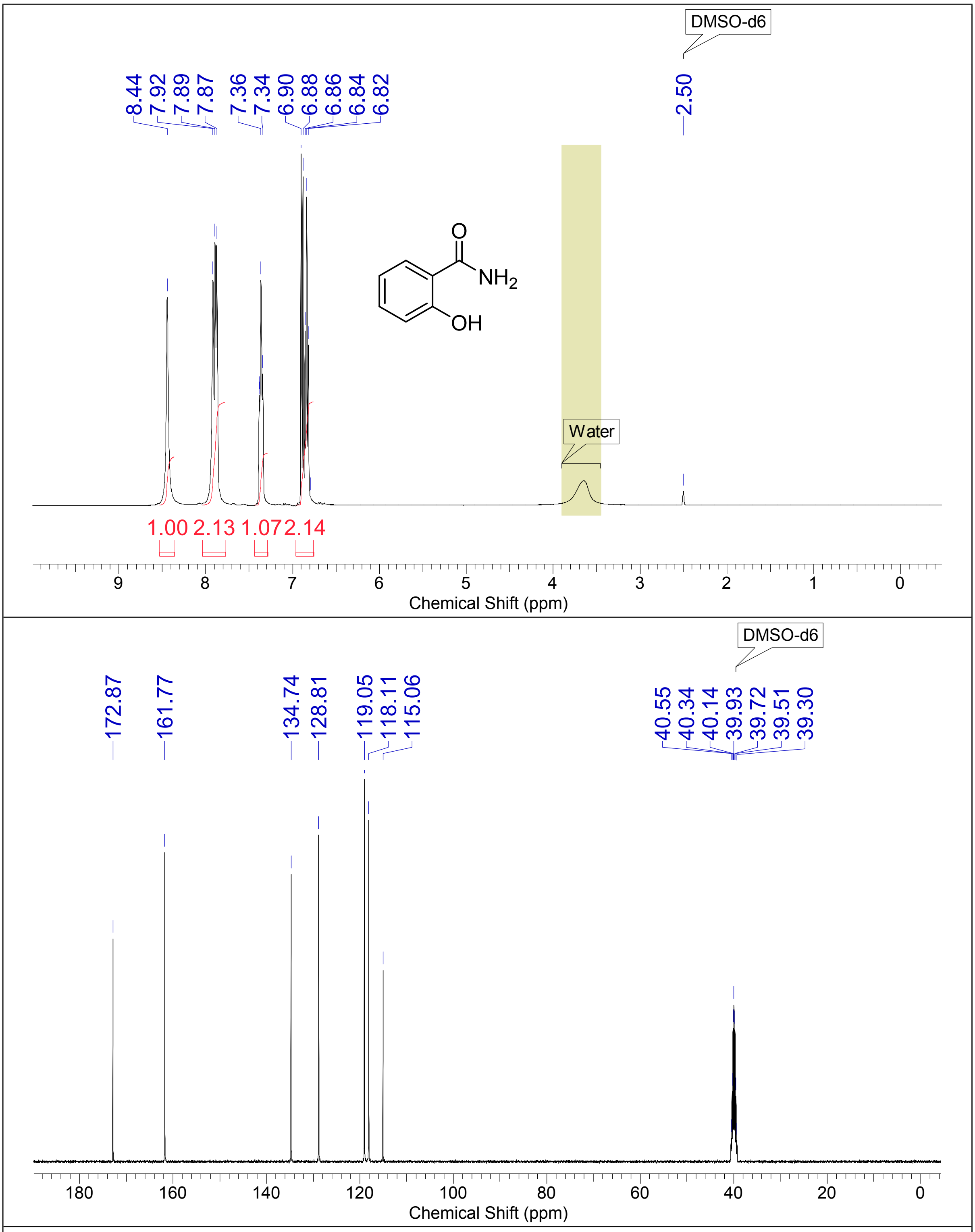

2-hydroxybenzamide(3q)

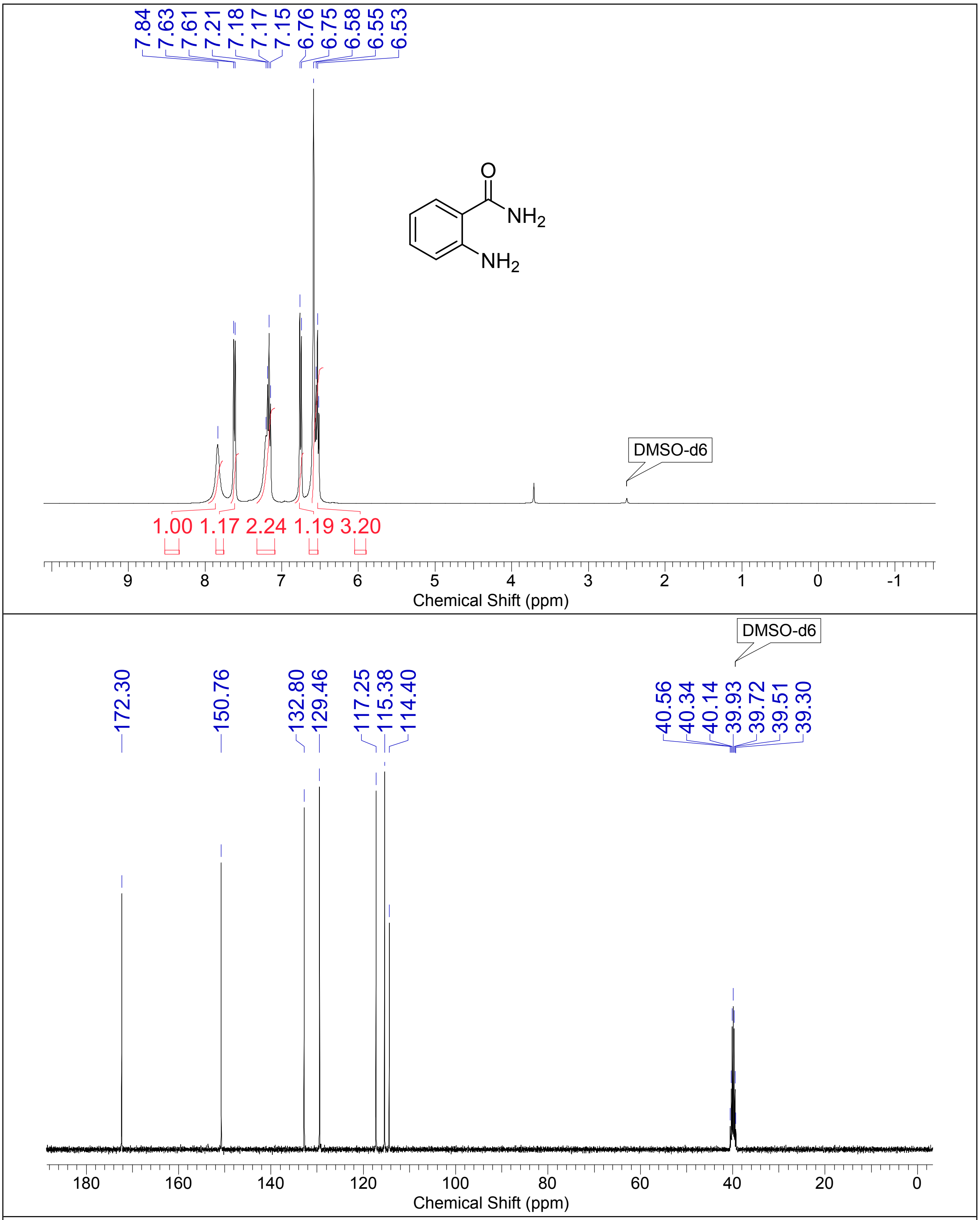

2-aminobenzamide(3r)

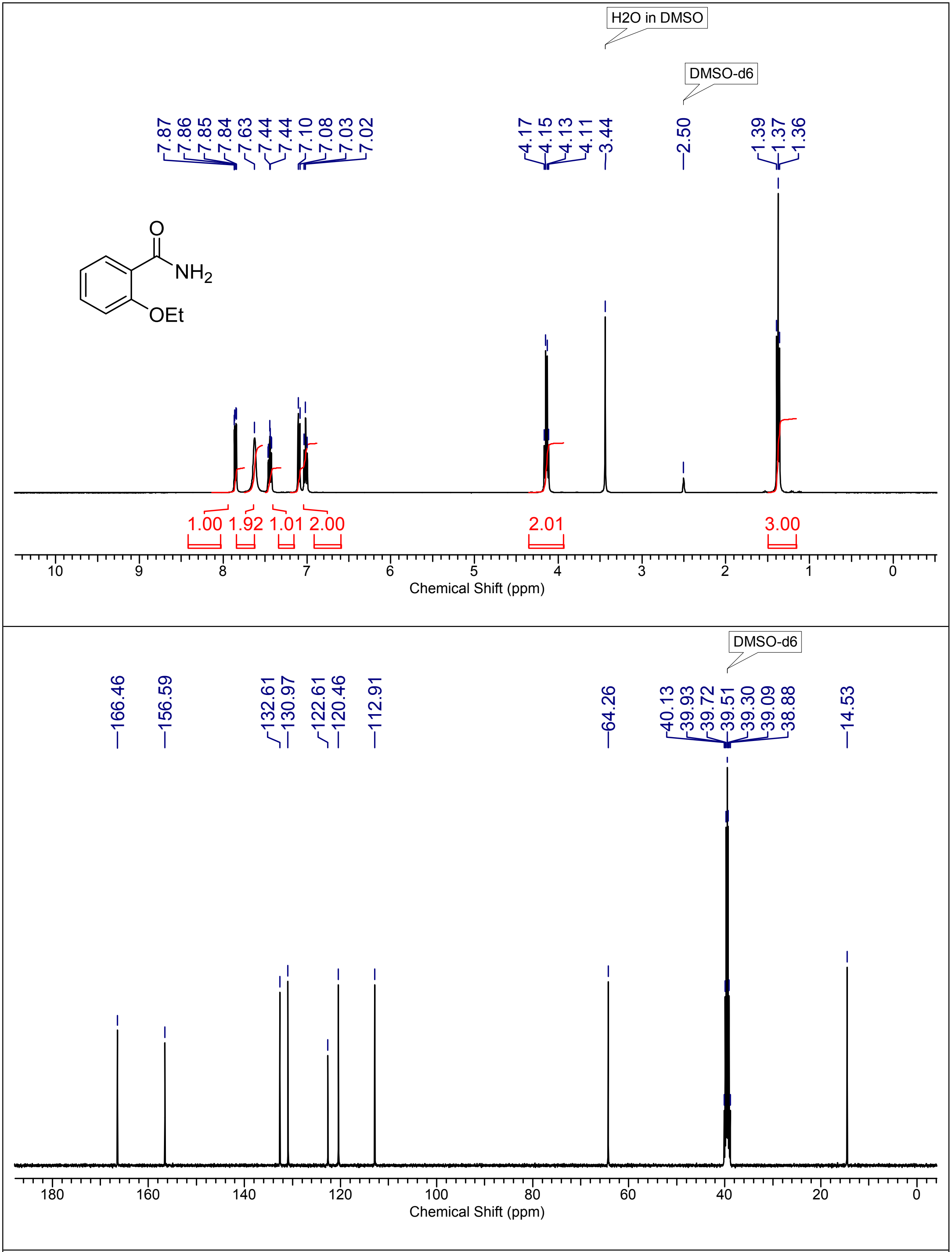

Ethnazamide(3s)

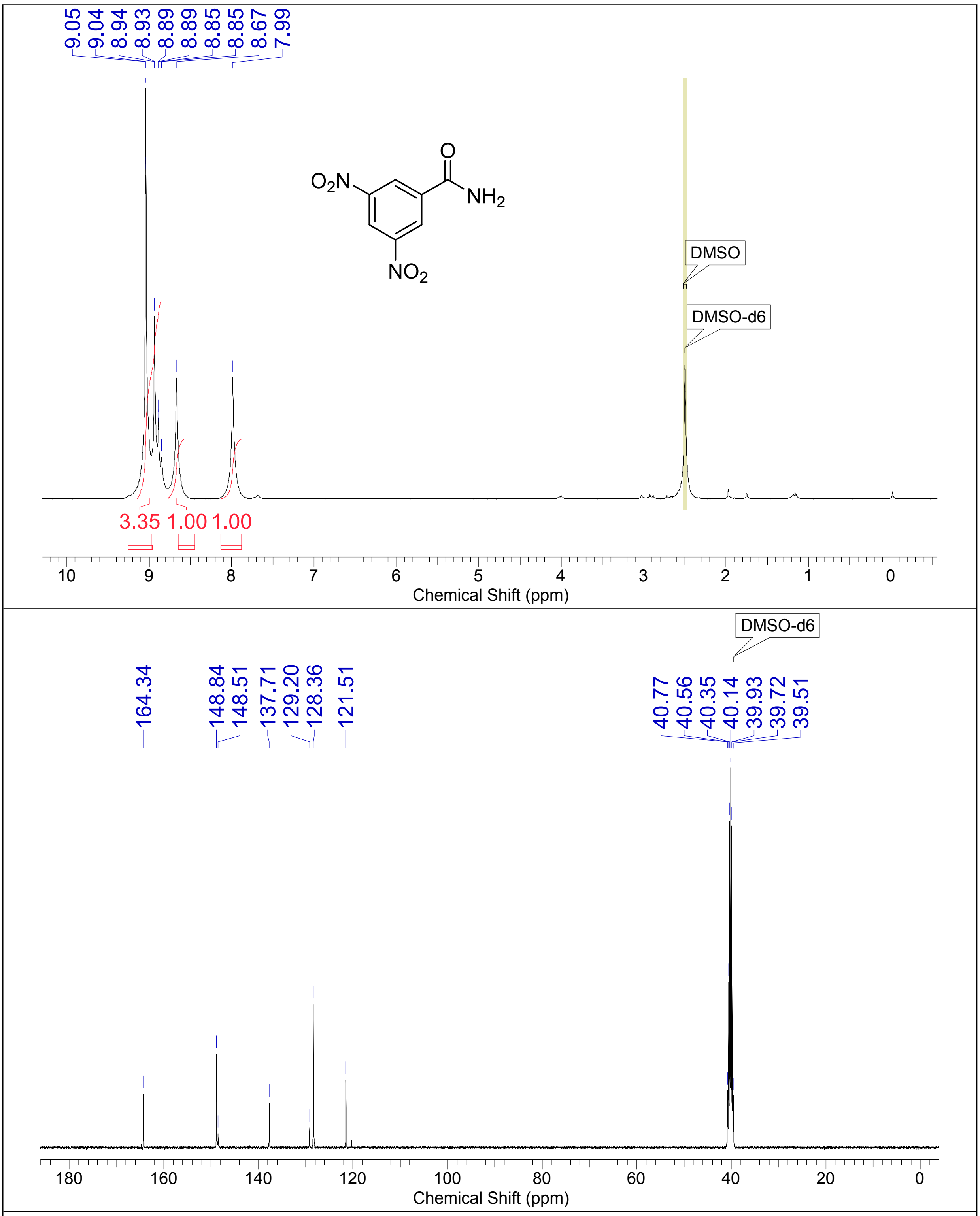

3,5-dinitrobenzamide(3t)

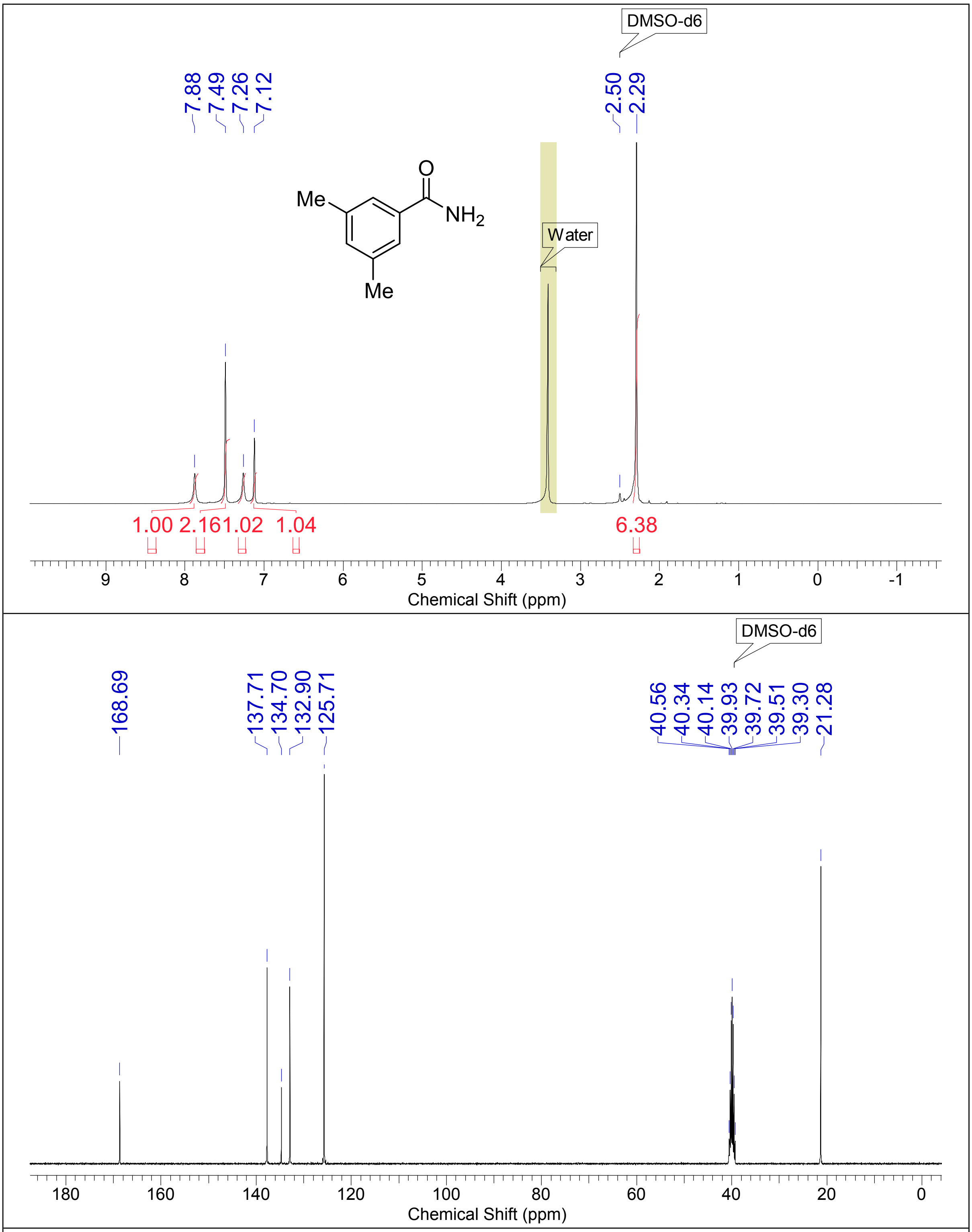

3,5-dimethylbenzamide(3u)

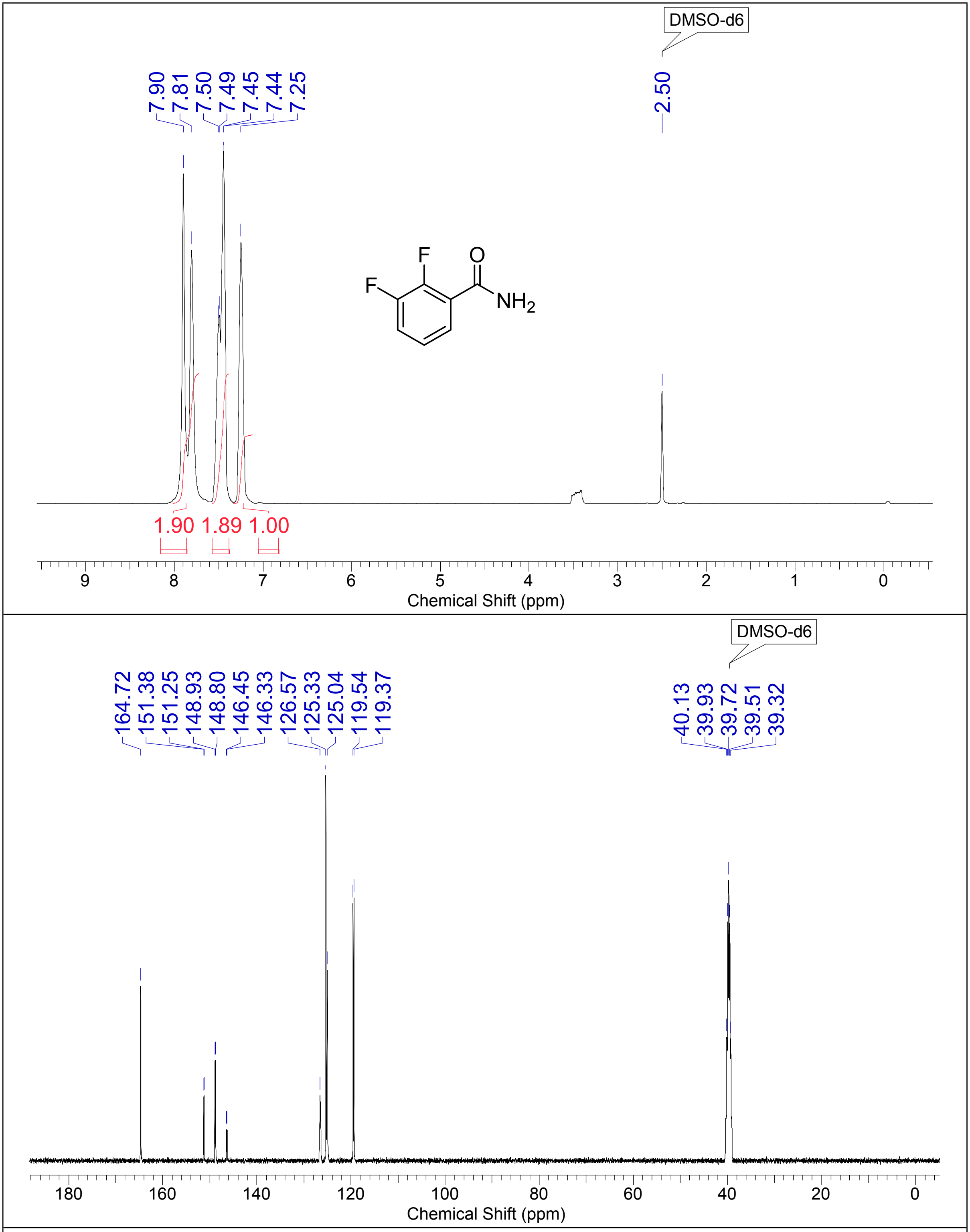

2,3-difluorobenzamide(3v)

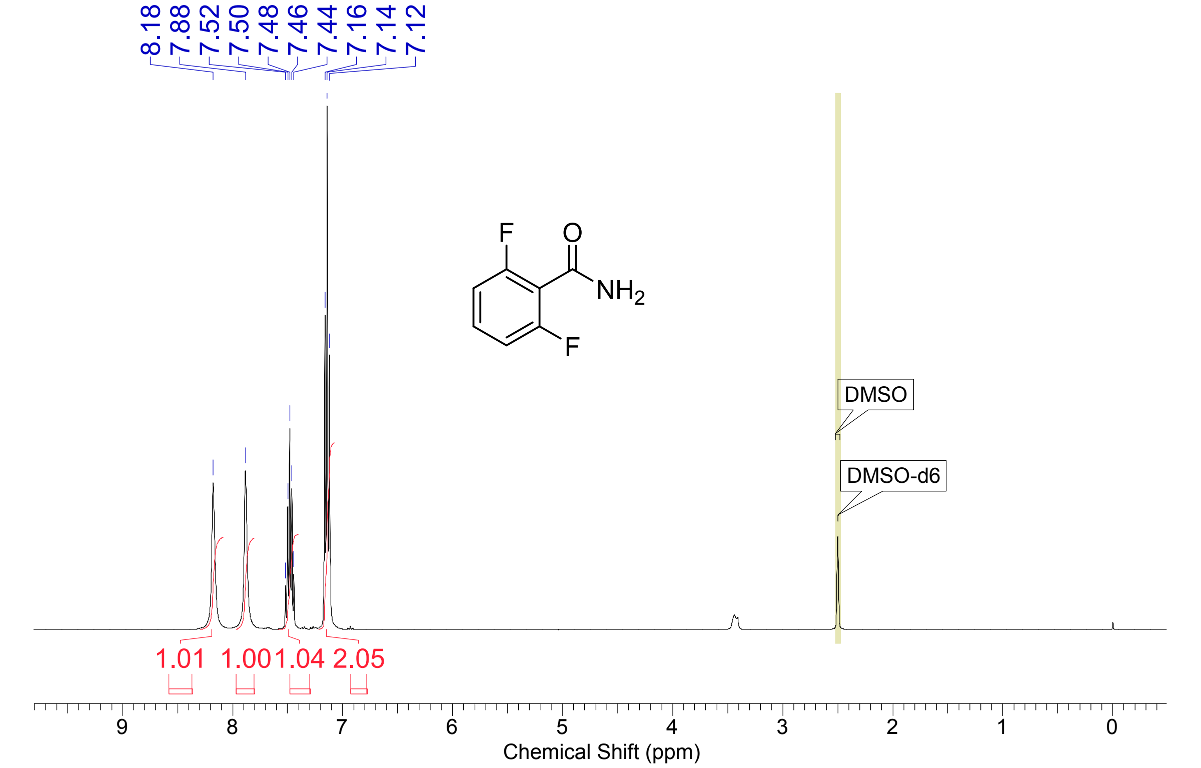

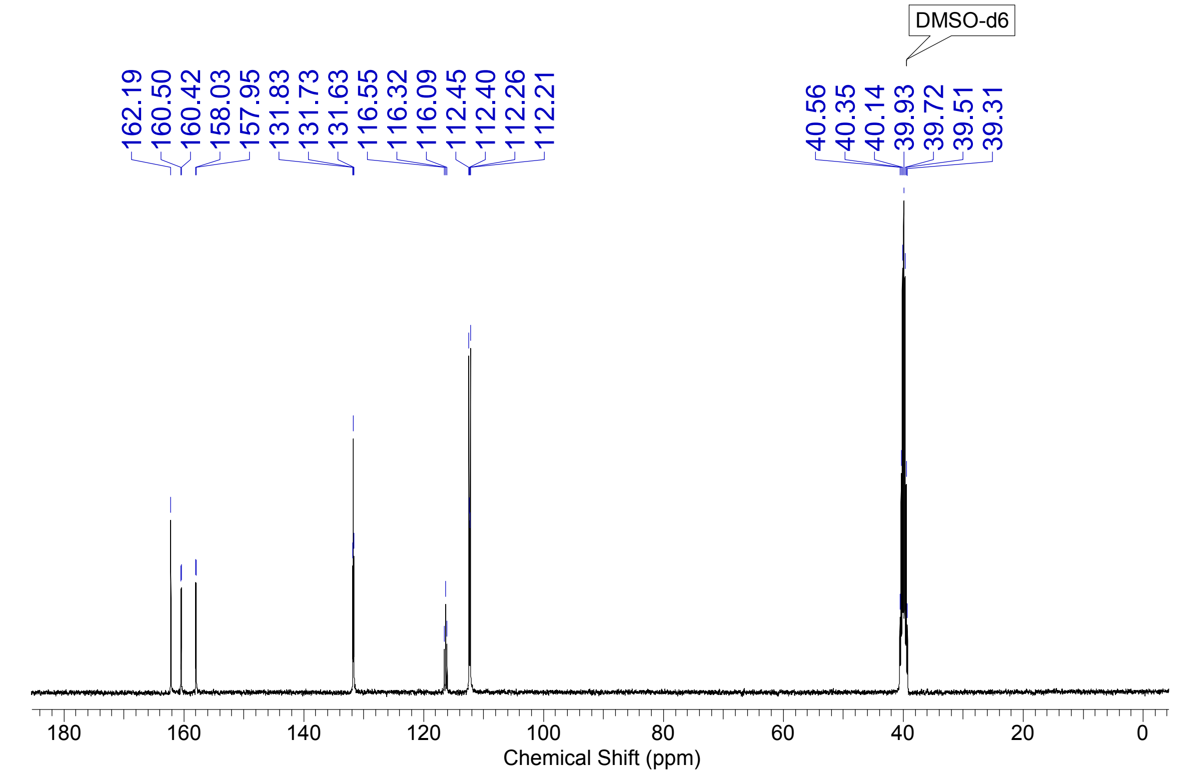

2,6-difluorobenzamide(3w)

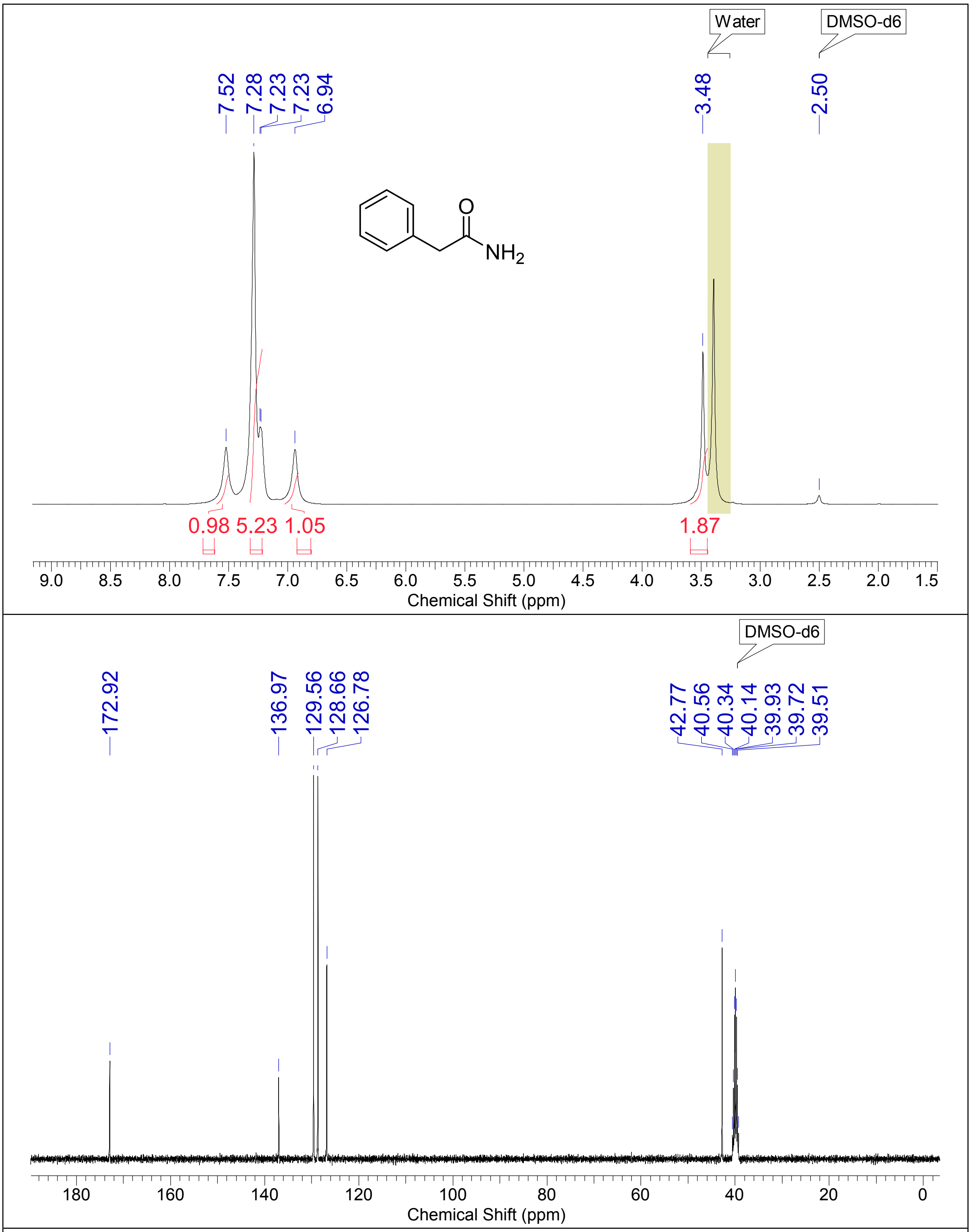

2-phenylacetamide(3x)

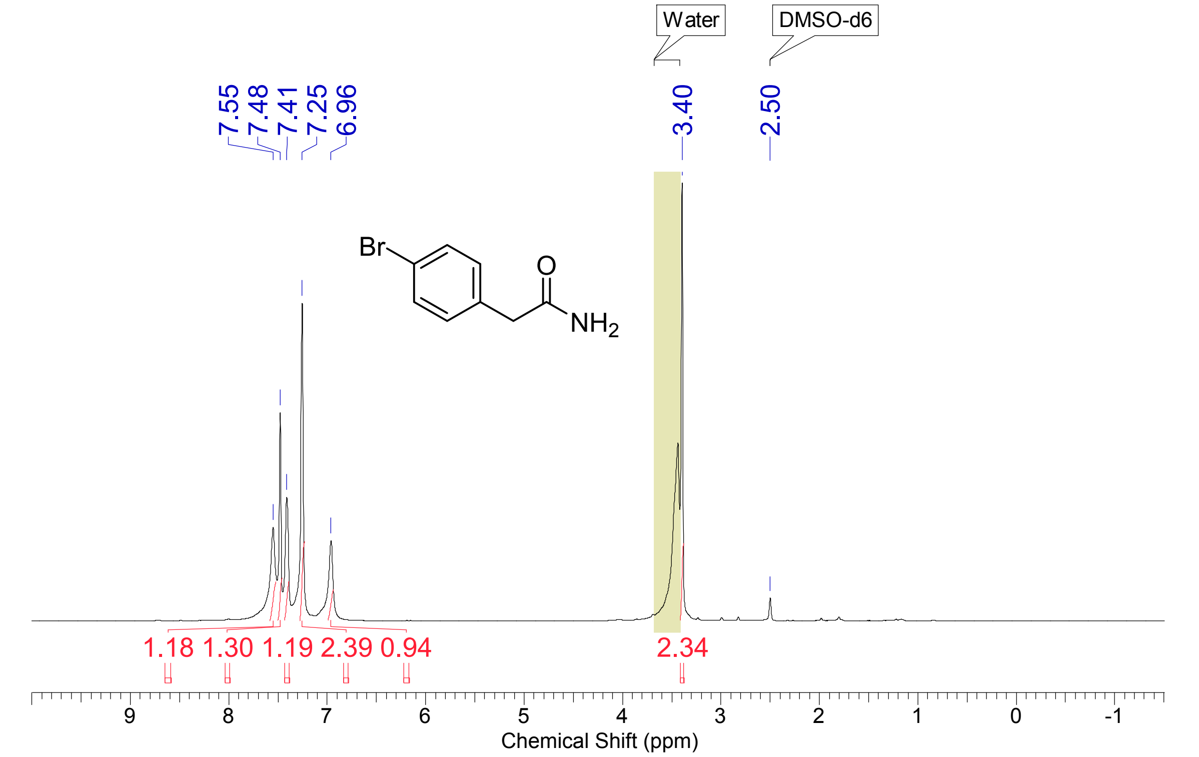

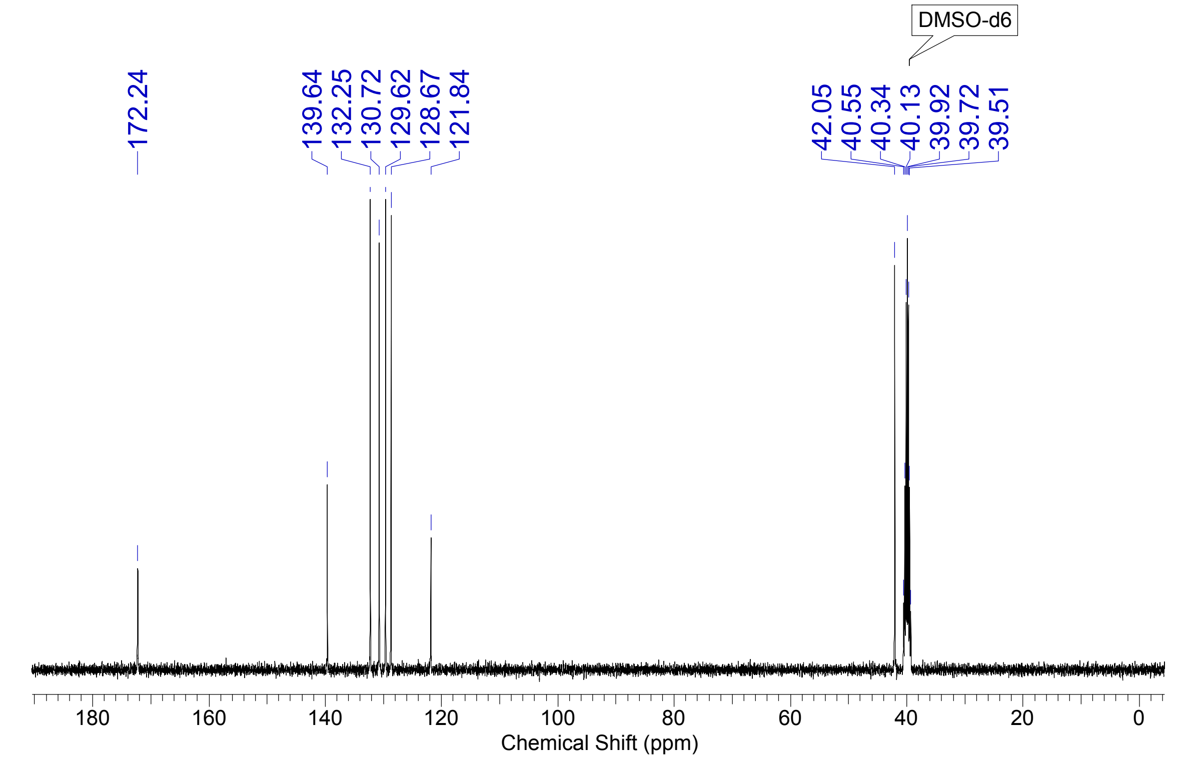

2-(4-bromophenyl)acetamide(3y)

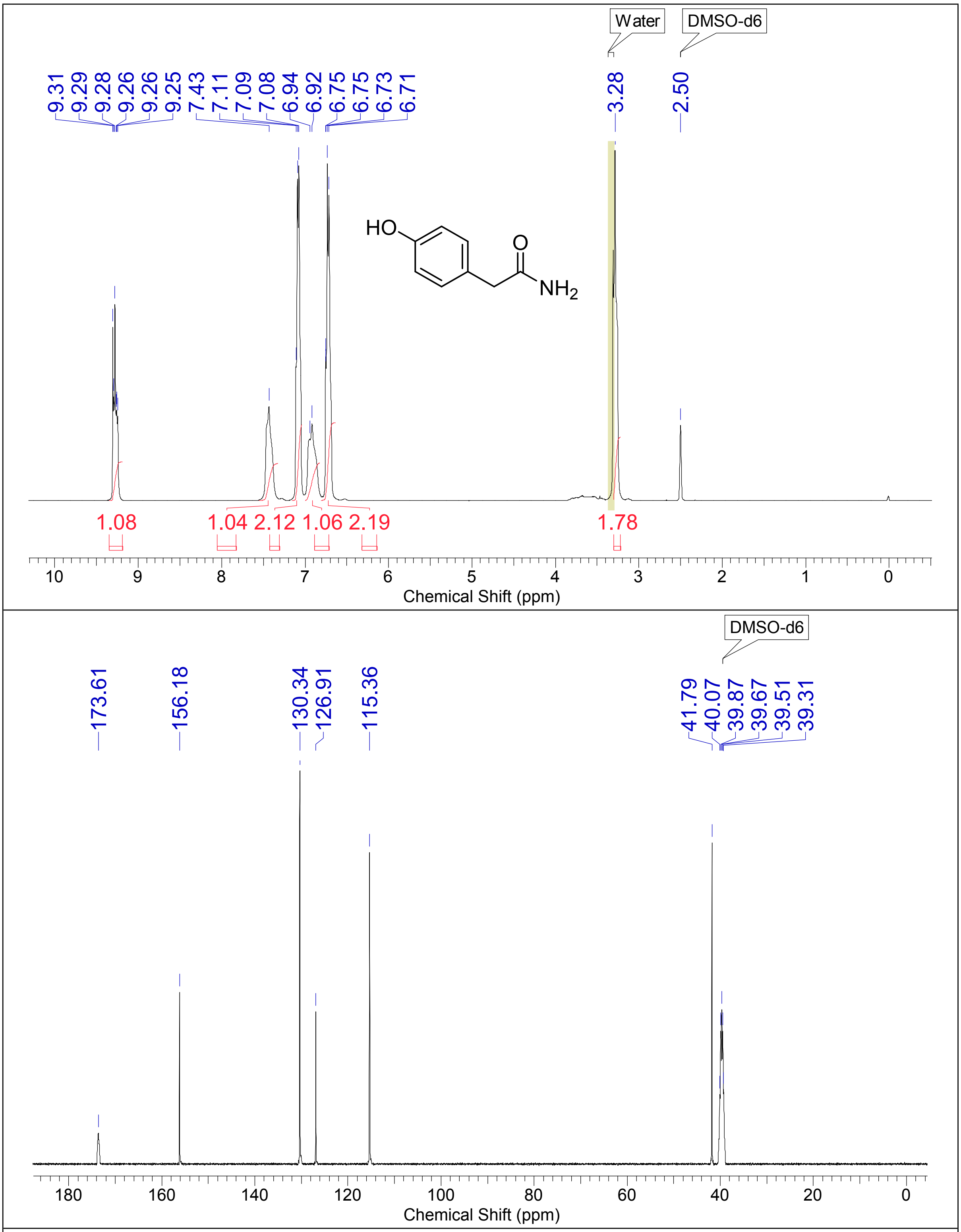

2-(4-hydroxyphenyl)acetamide(3z)

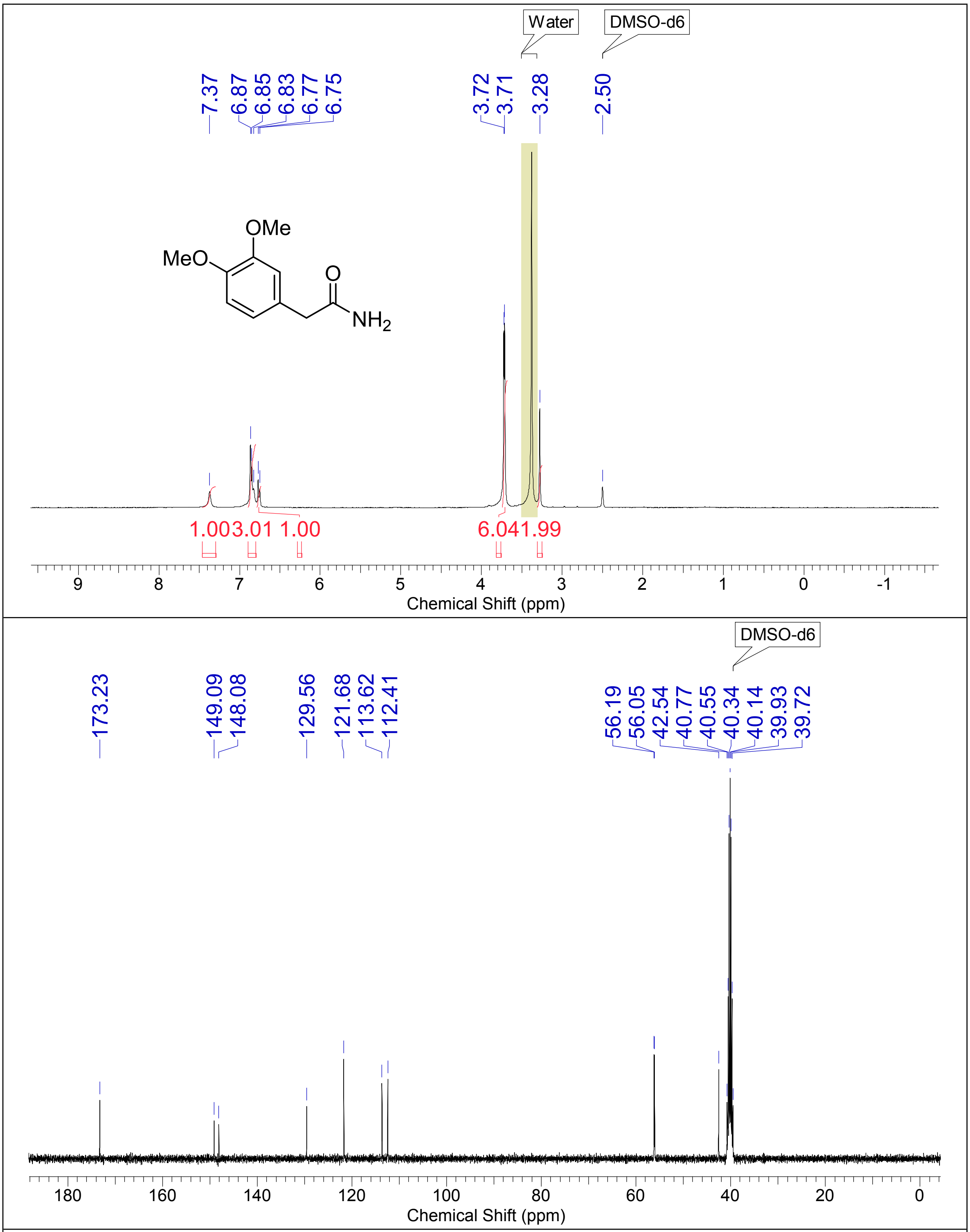

2-(3,4-dimethoxyphenyl)acetamide(3aa)

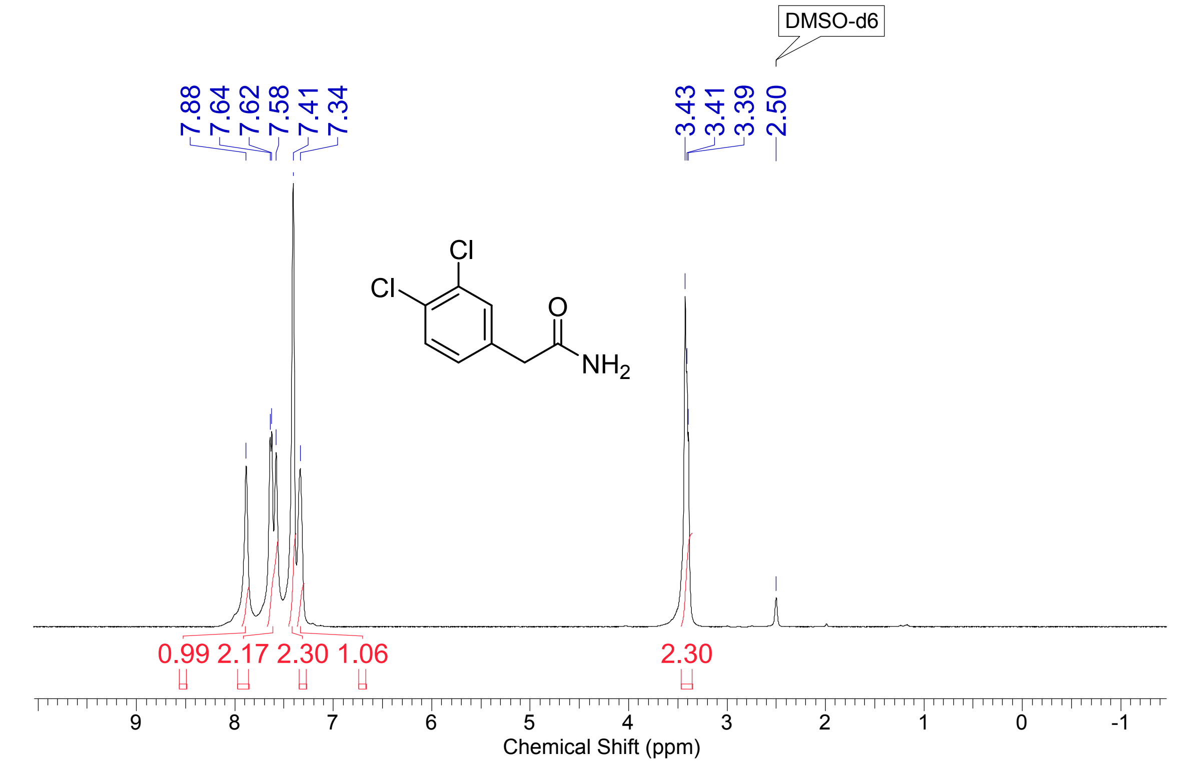

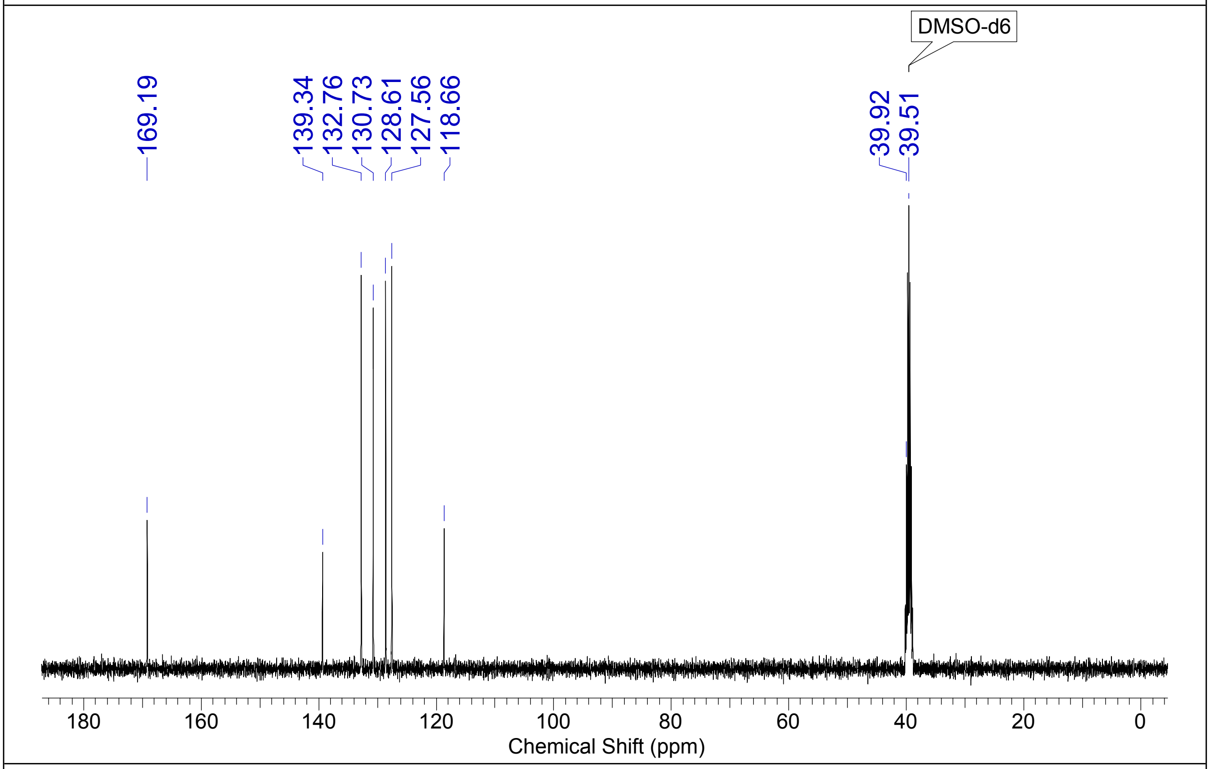

2-(3,4-dichlorophenyl)acetamide(3ab)

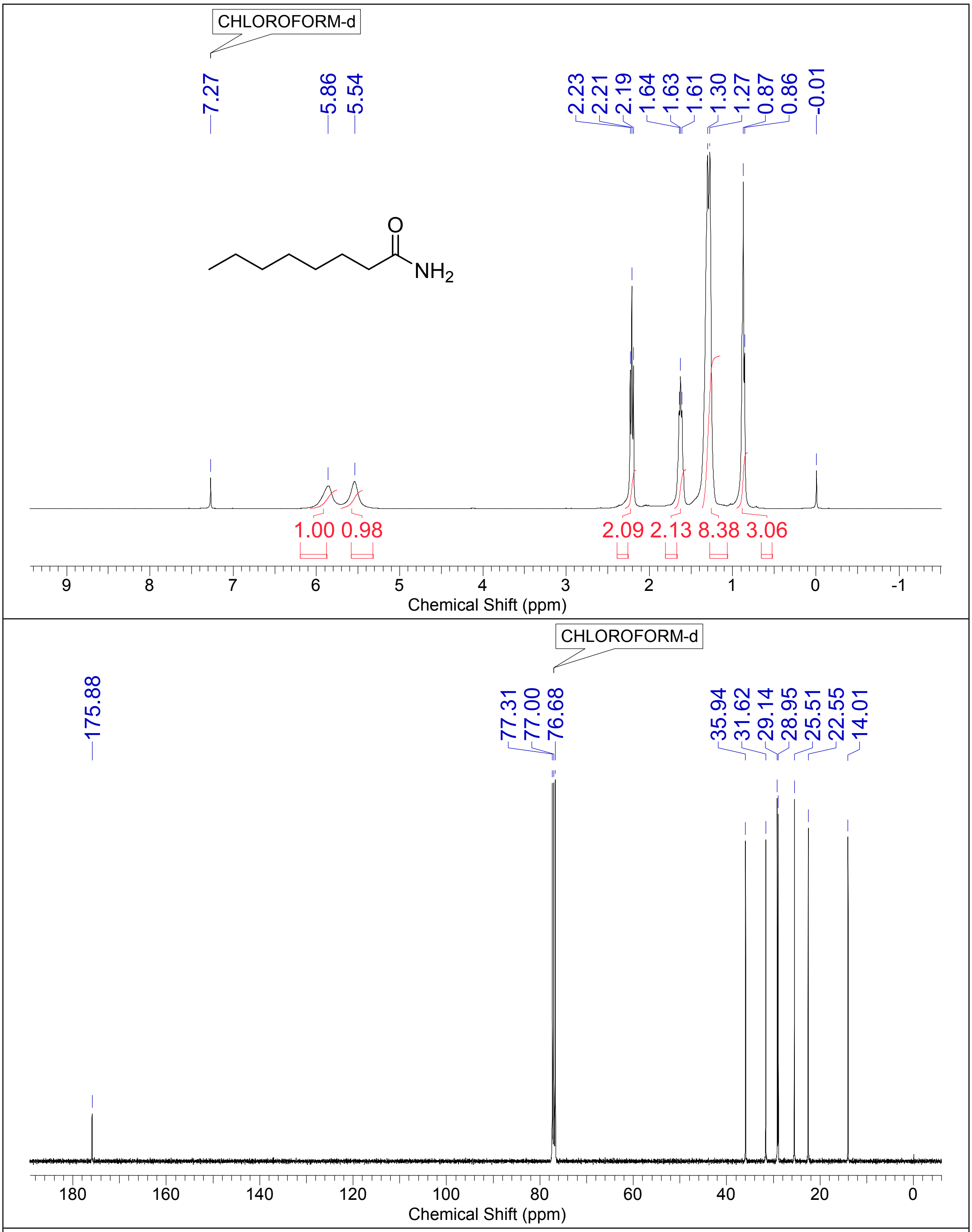

octanamide(3ac)

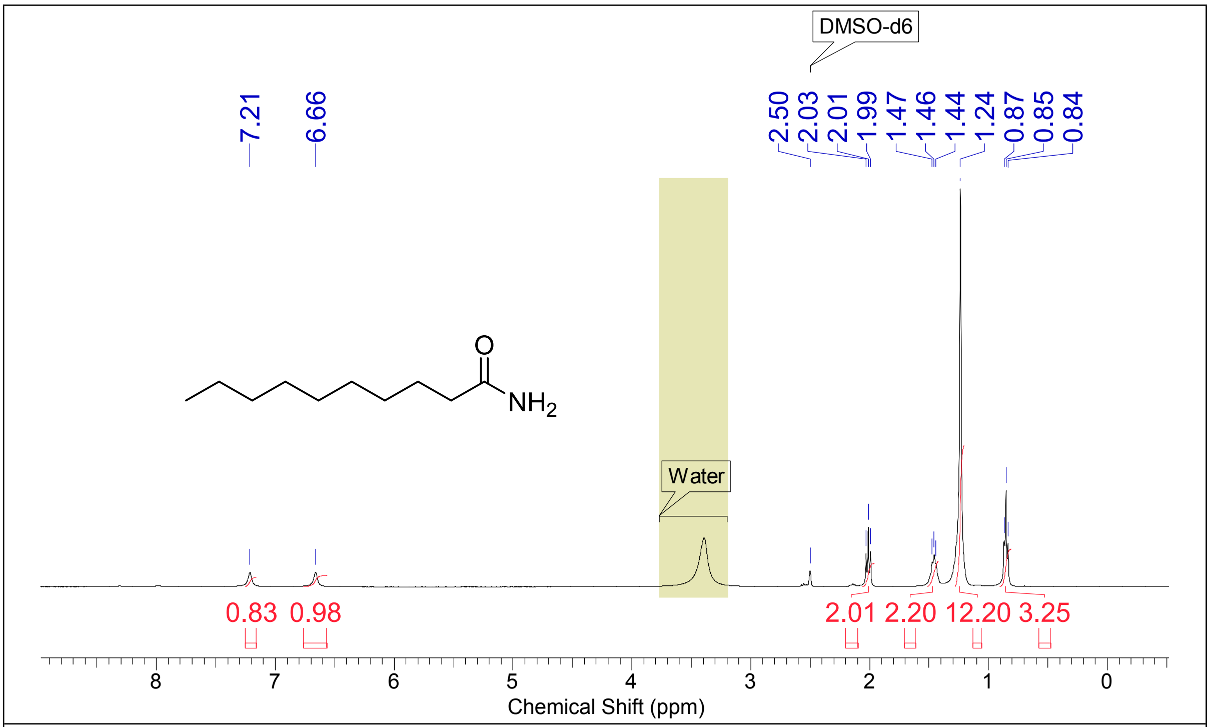

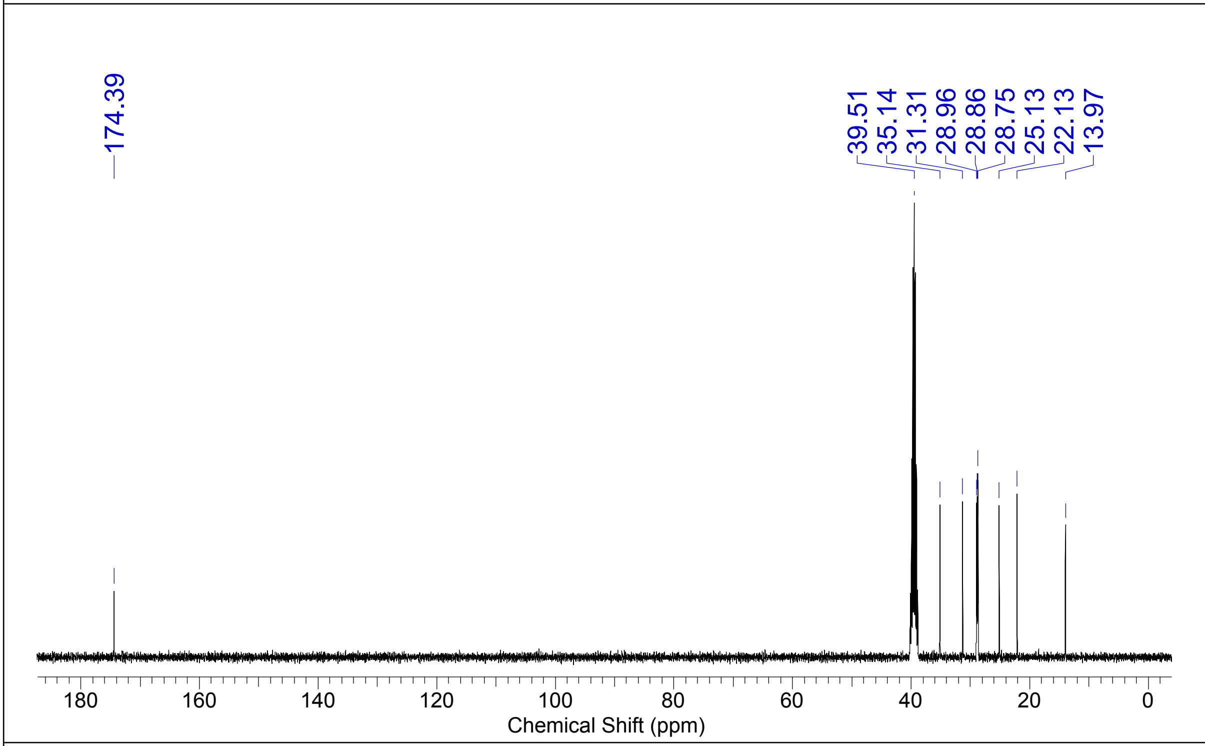

decanamide(3ad)

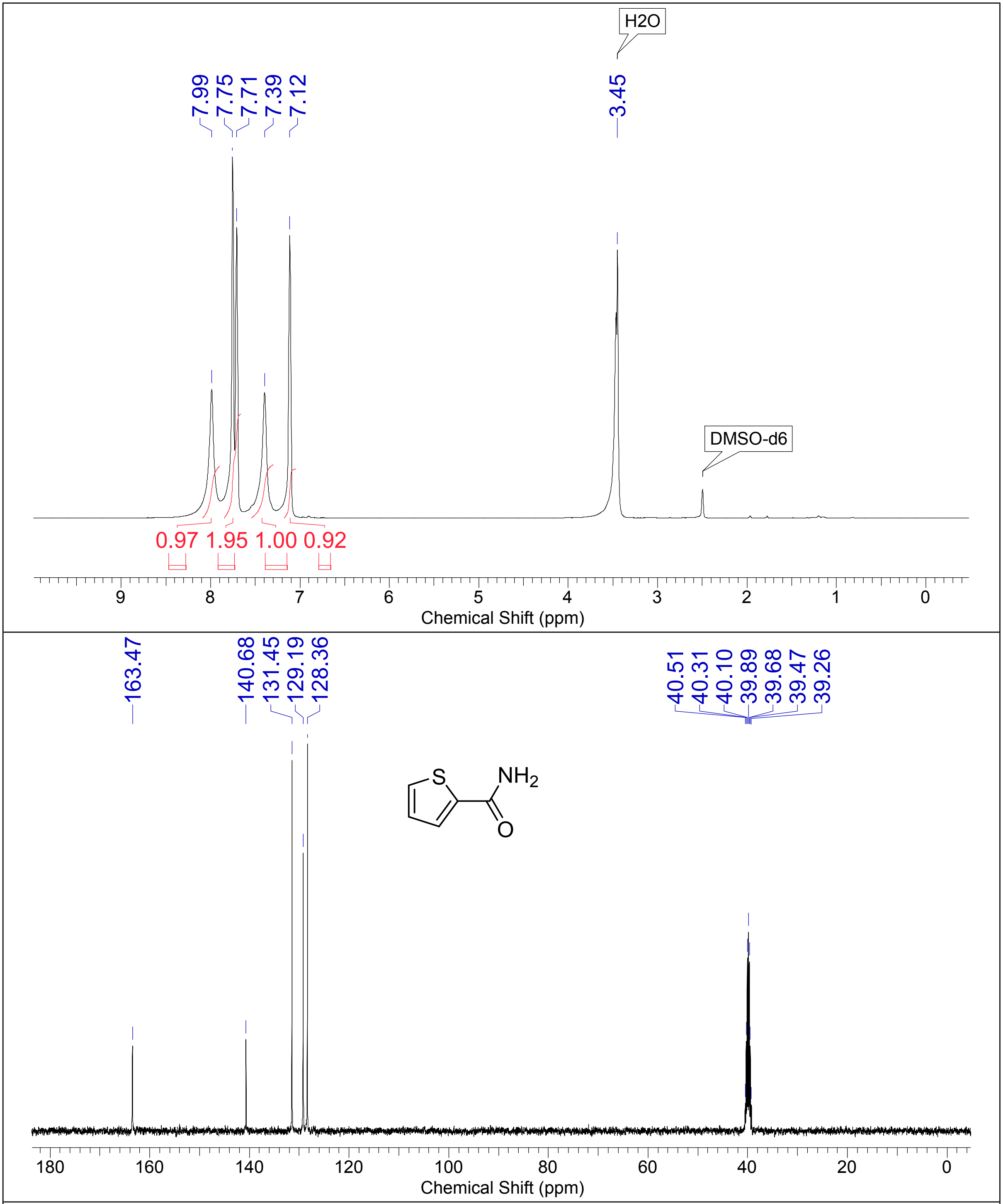

thiophene-2-carboxamide(3ae)

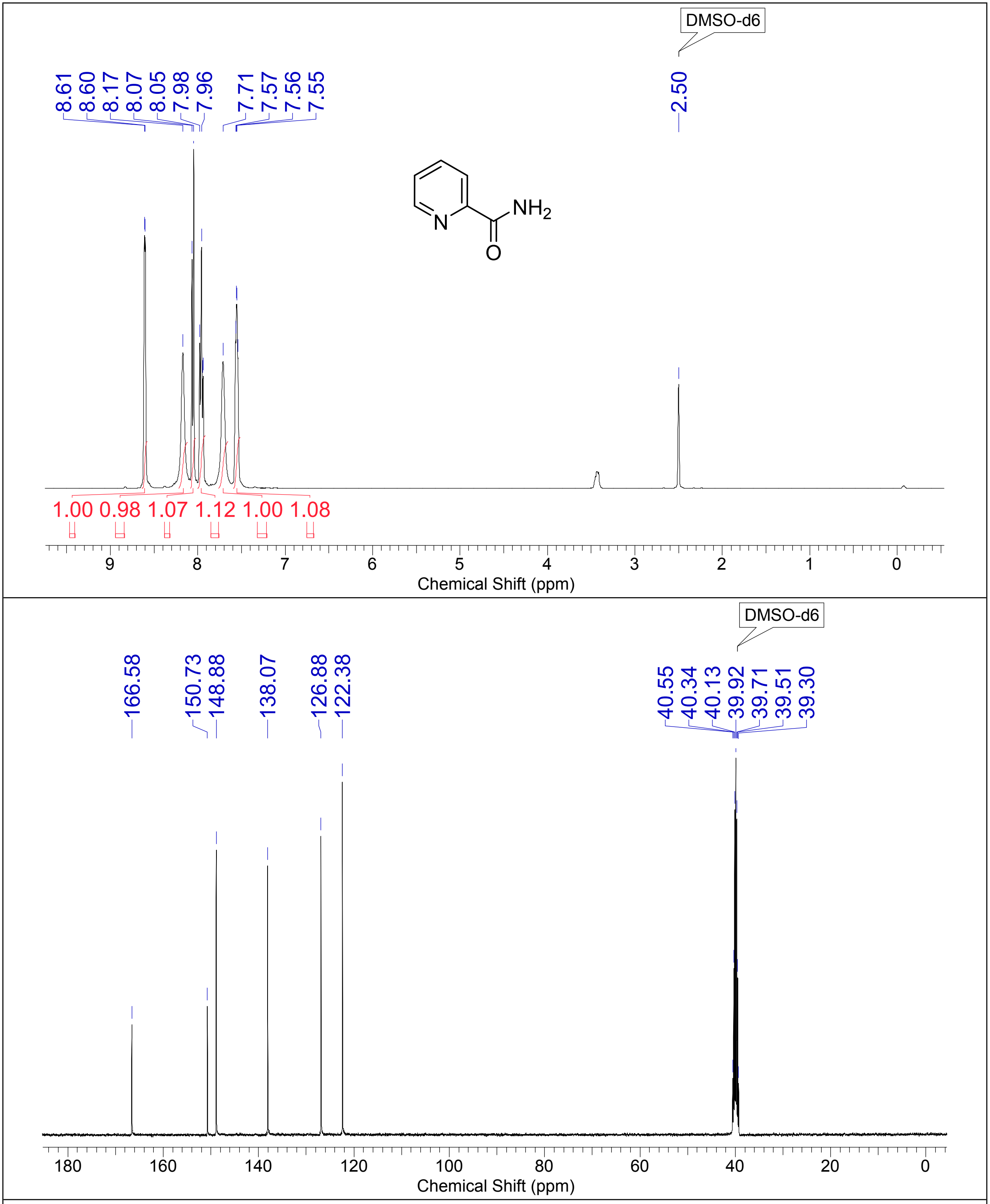

2-picolinamide(3af)

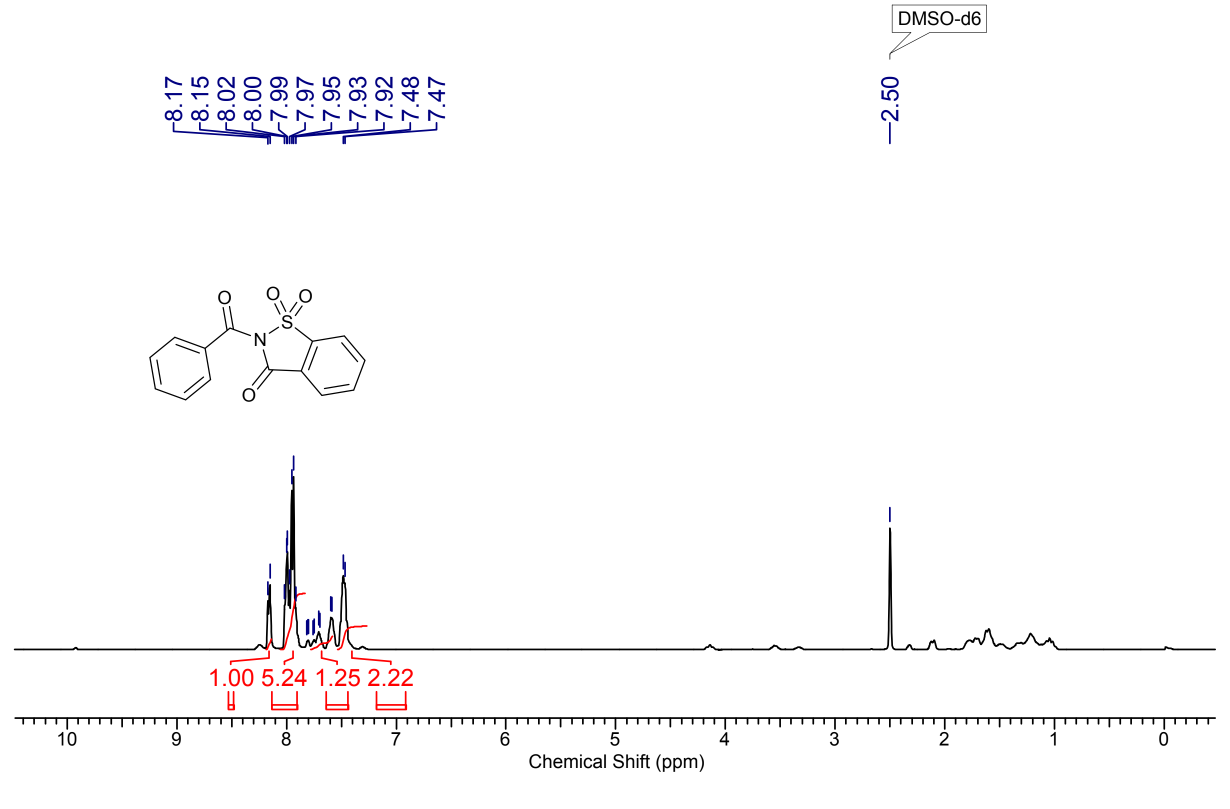

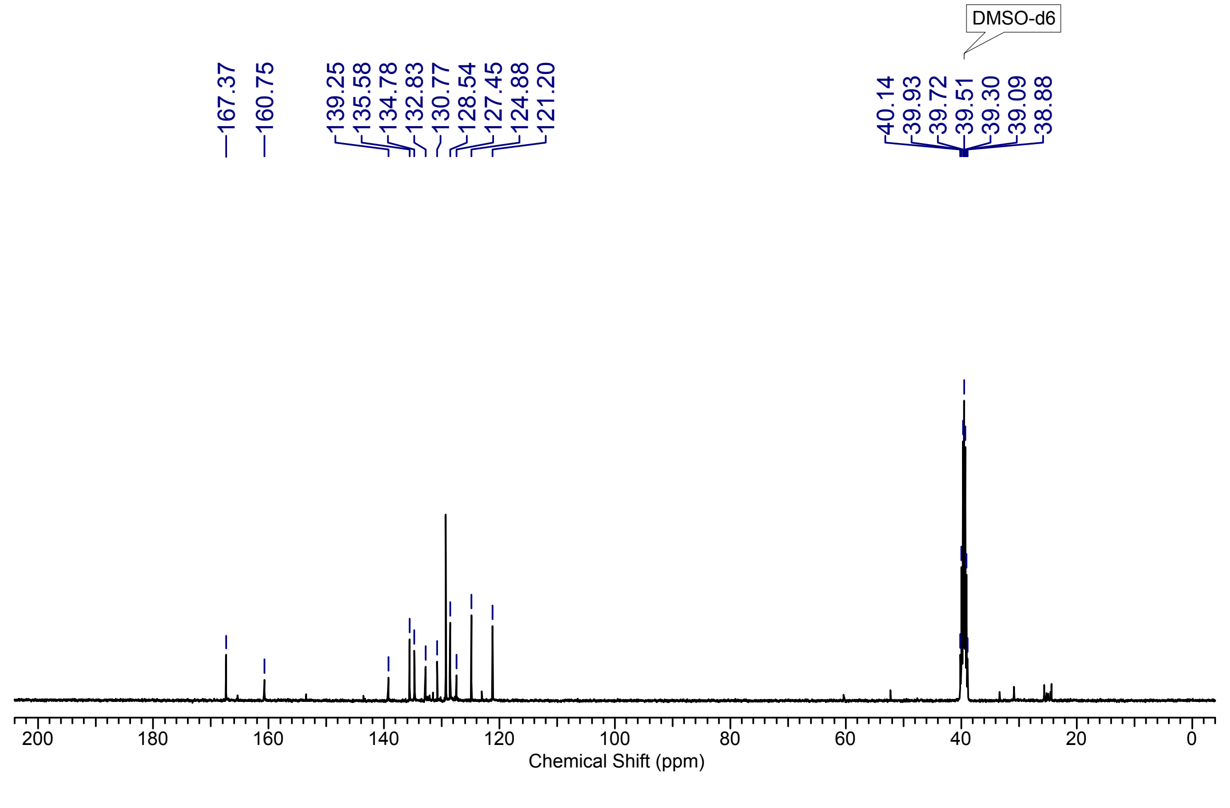

2-benzoylbenzo[d]isothiazol-3(2H)-one 1,1-dioxide (8)

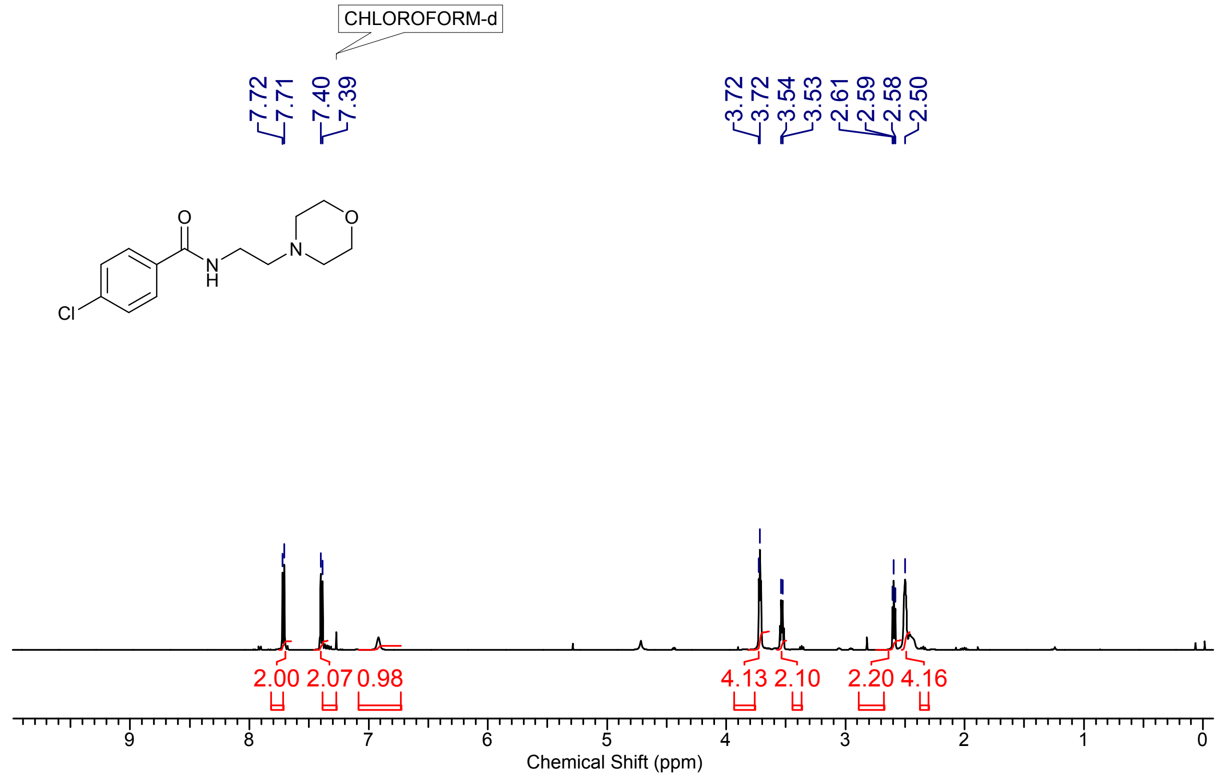

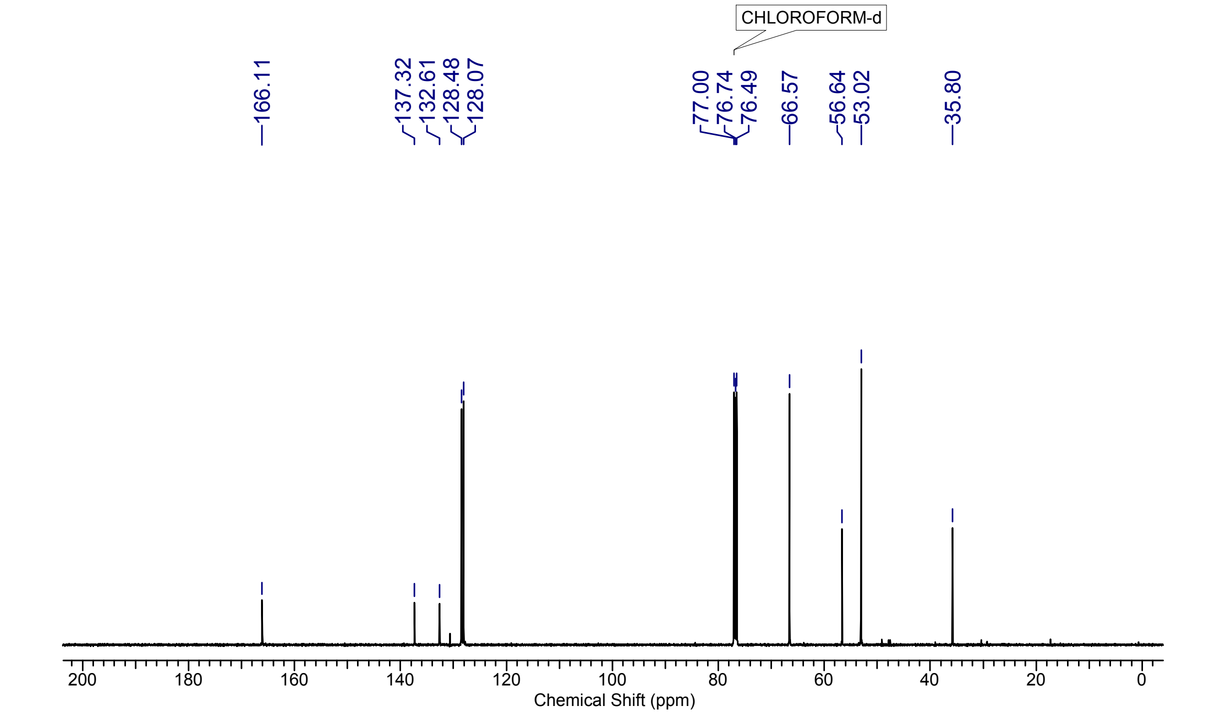

Moclobemide (7)

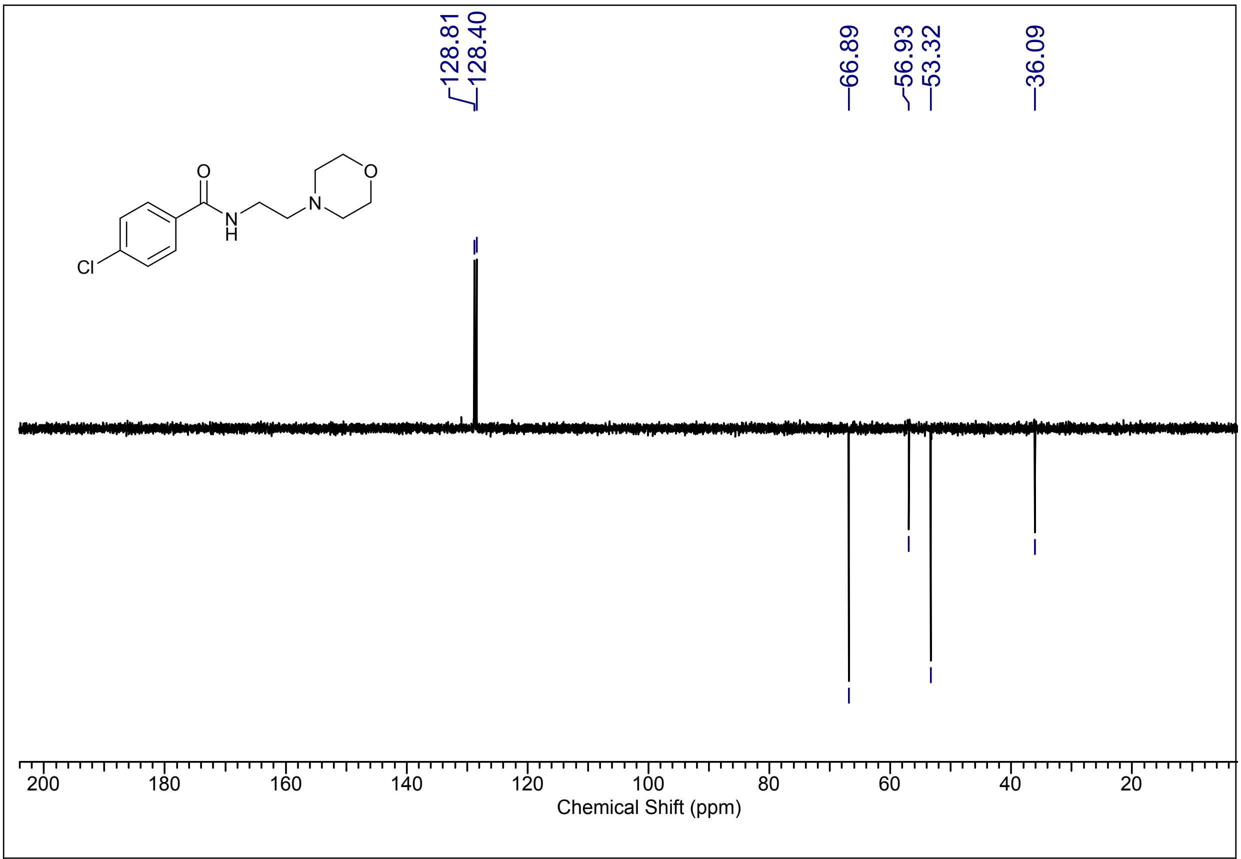

DEPT of Moclobemide (7)

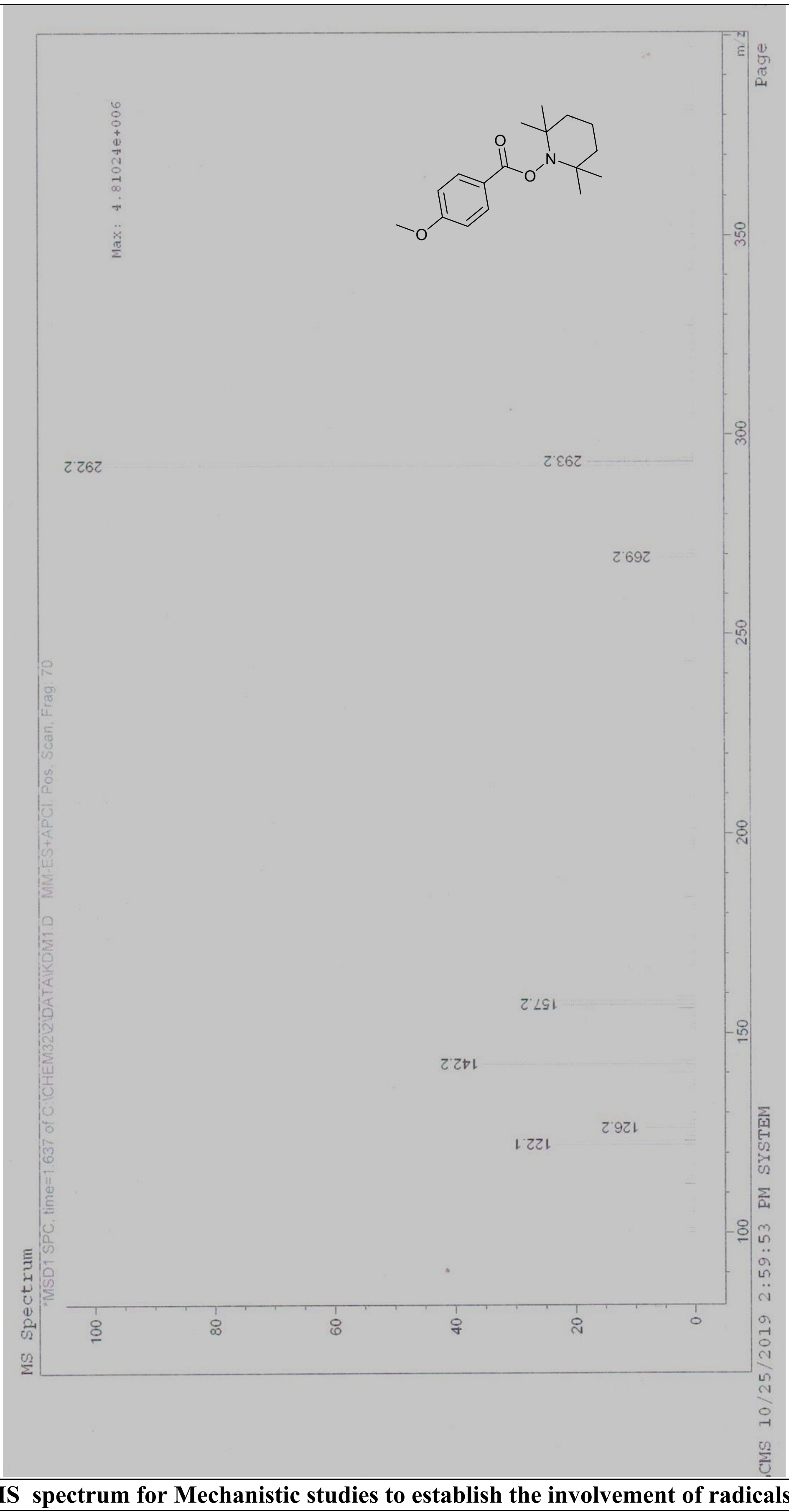

LC-MS spectrum for Mechanistic studies to establish the involvement of radicals (10)

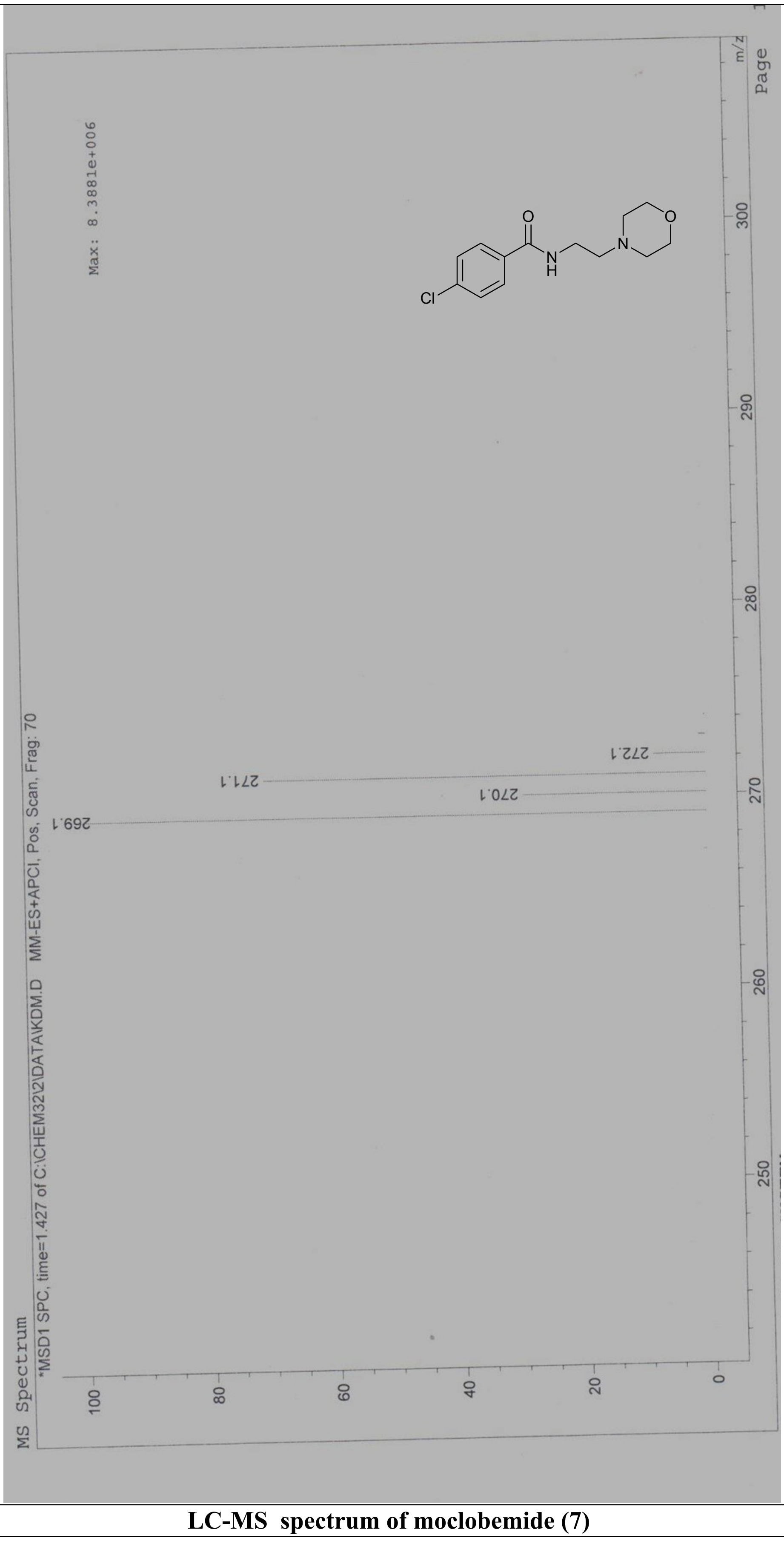

LC-MS spectrum of moclobemide (7)

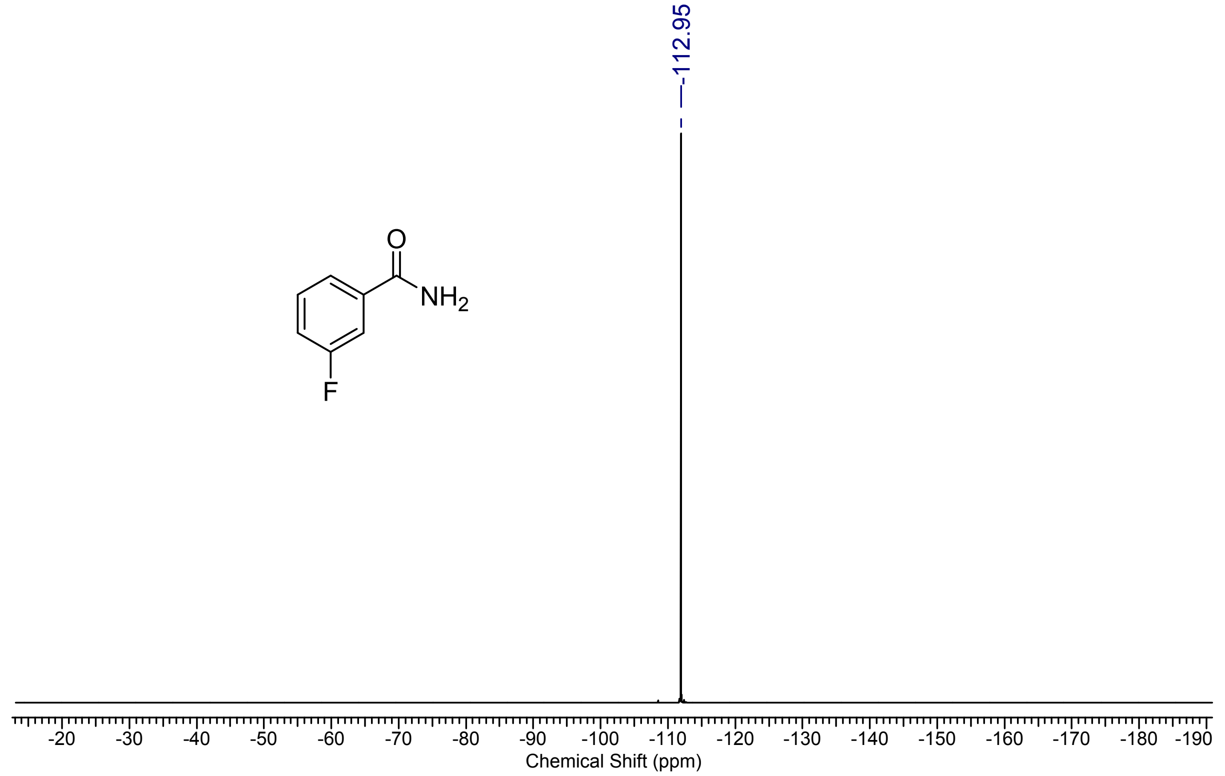

3-fluorobenzamide(3k)

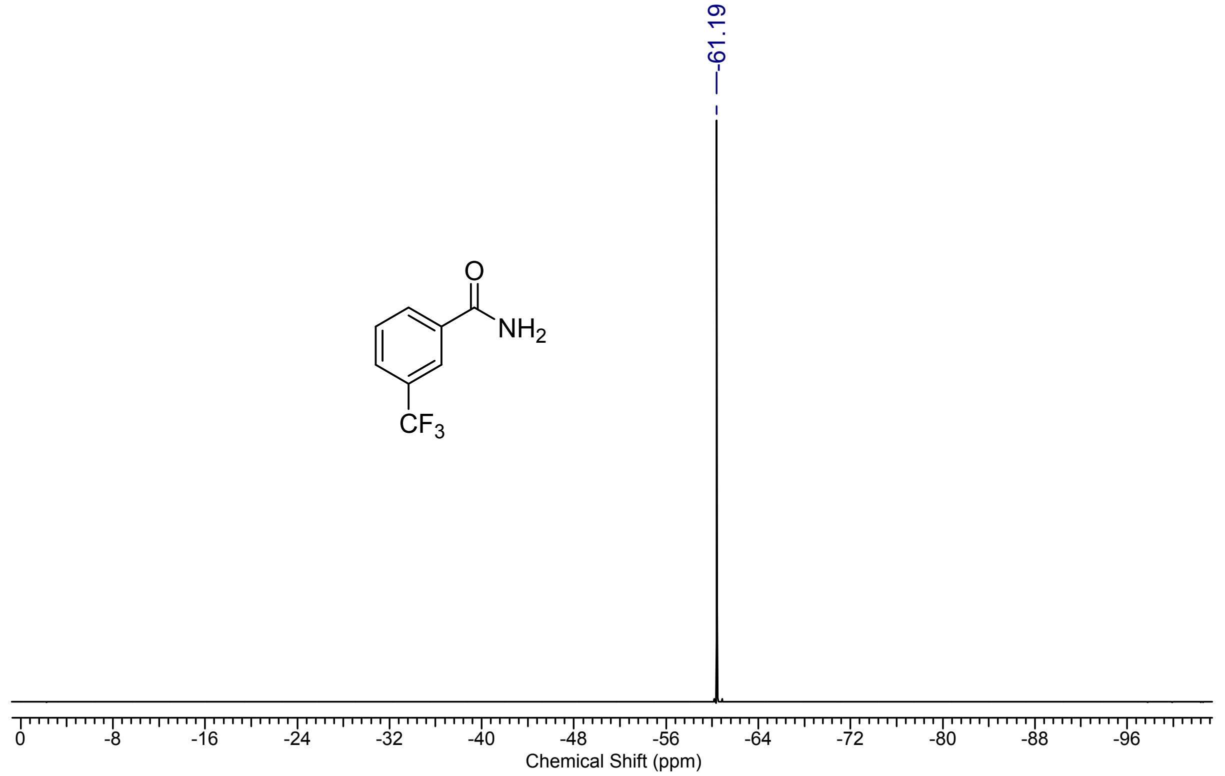

3-(trifluoromethyl)benzamide(3l)

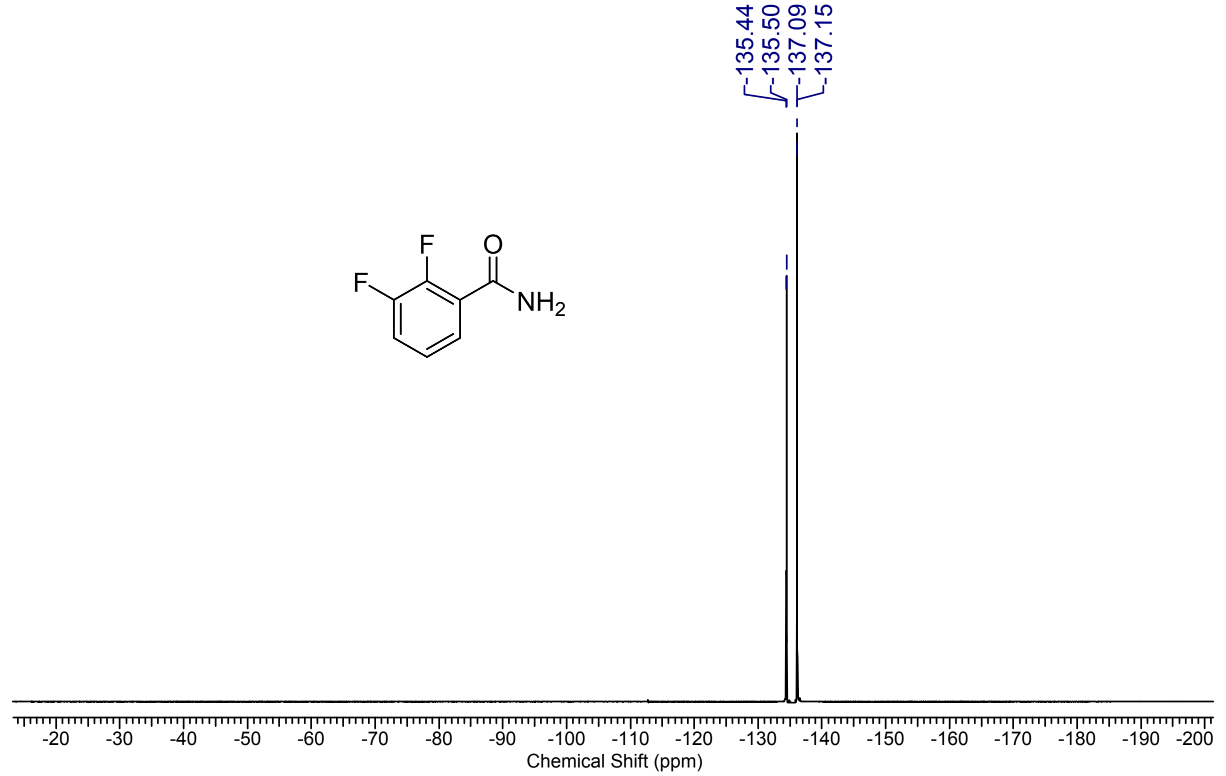

2,3-difluorobenzamide(3v)

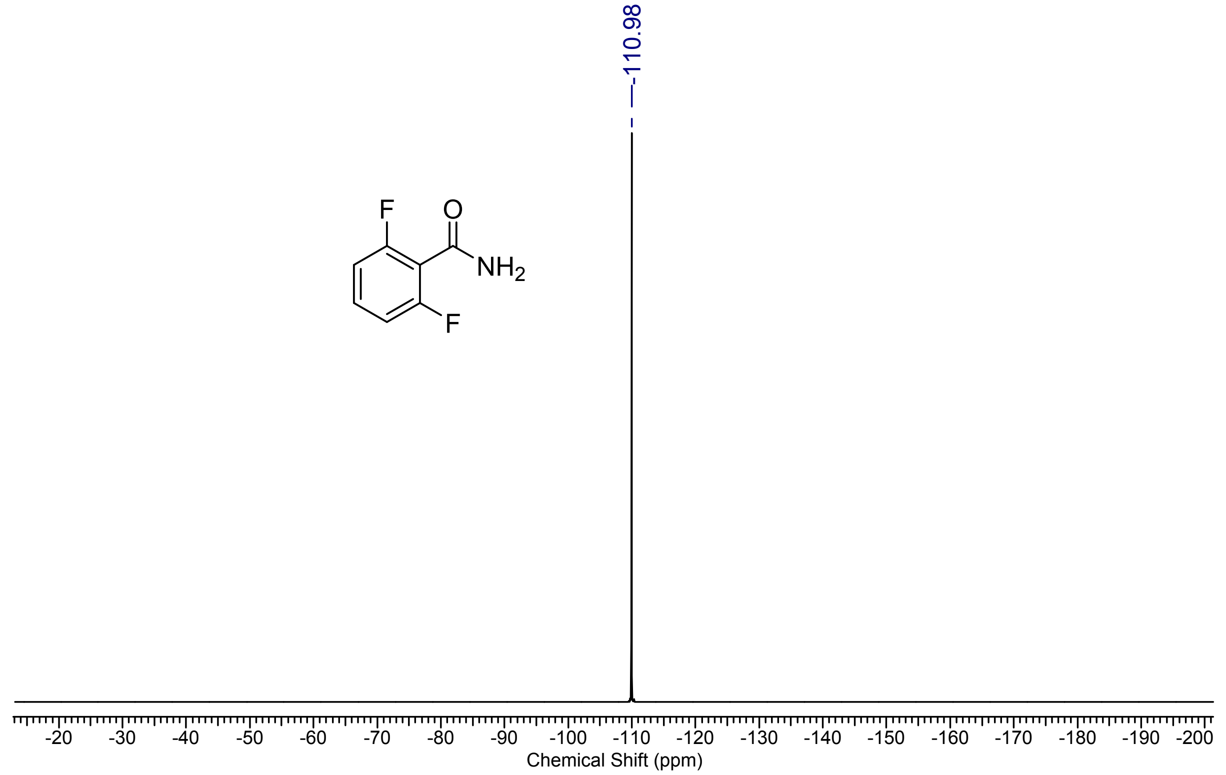

2,6-difluorobenzamide(3w)
